# Exploring Structure-Activity Relationship in Tacrine-Squaramide Derivatives as Potent Cholinesterase Inhibitors

**DOI:** 10.3390/biom9080379

**Published:** 2019-08-19

**Authors:** Barbora Svobodova, Eva Mezeiova, Vendula Hepnarova, Martina Hrabinova, Lubica Muckova, Tereza Kobrlova, Daniel Jun, Ondrej Soukup, María Luisa Jimeno, José Marco-Contelles, Jan Korabecny

**Affiliations:** 1 Department of Toxicology and Military Pharmacy, Faculty of Military Health Sciences, Trebesska 1575, 500 01 Hradec Kralove, Czech Republic; 2 Biomedical Research Centre, University Hospital Hradec Kralove, Sokolska 581, 500 05 Hradec Kralove, Czech Republic; 3 Laboratory of Medicinal Chemistry, Institute of General Organic Chemistry, Juan de la Cierva 3, 28006-Madrid, Spain; 4 Centro de Química Orgánica “Lora-Tamayo” (CSIC), C/Juan de la Cierva 3, 28006-Madrid, Spain

**Keywords:** tacrine, bis(7)-tacrine, 6-chlorotacrine, 7-methoxytacrine, squaramides, Alzheimer’s disease, cholinesterases, in vitro, in silico

## Abstract

Tacrine was the first drug to be approved for Alzheimer’s disease (AD) treatment, acting as a cholinesterase inhibitor. The neuropathological hallmarks of AD are amyloid-rich senile plaques, neurofibrillary tangles, and neuronal degeneration. The portfolio of currently approved drugs for AD includes acetylcholinesterase inhibitors (AChEIs) and *N*-methyl-d-aspartate (NMDA) receptor antagonist. Squaric acid is a versatile structural scaffold capable to be easily transformed into amide-bearing compounds that feature both hydrogen bond donor and acceptor groups with the possibility to create multiple interactions with complementary sites. Considering the relatively simple synthesis approach and other interesting properties (rigidity, aromatic character, H-bond formation) of squaramide motif, we combined this scaffold with different tacrine-based derivatives. In this study, we developed 21 novel dimers amalgamating squaric acid with either tacrine, 6-chlorotacrine or 7-methoxytacrine representing various AChEIs. All new derivatives were evaluated for their anti-cholinesterase activities, cytotoxicity using HepG2 cell line and screened to predict their ability to cross the blood-brain barrier. In this contribution, we also report in silico studies of the most potent AChE and BChE inhibitors in the active site of these enzymes.

## 1. Introduction

Alzheimer’s disease (AD), the most common form of dementia [1], is symptomatically treated with inhibitors of acetylcholinesterase (AChE, 3.1.1.7). This type of serine protease catalyzes the hydrolysis of the neurotransmitter acetylcholine (ACh) to choline and acetic acid [2]. Low levels of ACh lead to cognitive impairment and dementia [3], thus the inactivation of AChE increases the pool of ACh and ameliorates the cognitive dysfunction associated with AD [4]. Three AChE inhibitors (AChEIs), namely, donepezil, galantamine, and rivastigmine, are the representatives of so-called “cholinergic hypothesis” used currently as the main drugs for AD treatment [5]. The etiology of AD is complex, and several factors are considered as causative. Indeed, AD has been recognized by deposition of two types of proteins, extracellularly accumulated β-amyloid (Aβ) protein due to abnormal processing of amyloid precursor protein (APP), and intracellular neurofibrillary tangles composed from hyperphosphorylated tau protein [6]. Furthermore, various other neurochemical abnormalities contribute to the progression of AD, such as oxidative stress [7], excitotoxicity [8], imbalance of biometals [9], or neuro-inflammation [10]. Memantine, an antagonist of *N*-methyl-d-aspartate (NMDA) receptor, is also approved for AD treatment [11].

**Figure 1:**
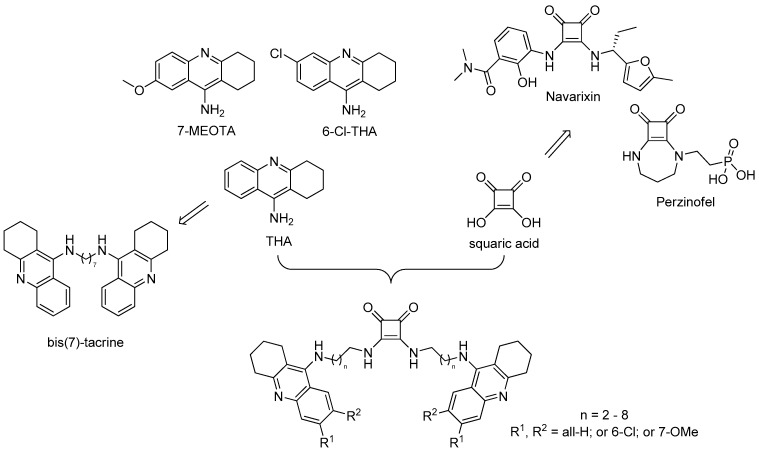
Schematic drawing for the design of tacrine-squaramide homodimers.

Tacrine (THA, 9-amino-1,2,3,4-tetrahydroacridine, Figure 1) is the centrally acting reversible AChEI that launched the market in 1993 as the first drug to alleviate symptoms of AD [12]. THA is a non-selective cholinesterase agent, inhibiting also butyrylcholinesterase (BChE, E.C. 3.1.1.8). Moreover, it affects several other biological systems, which contributes to its complex action against AD [13]. THA has been reported to also modulate muscarinic and nicotinic receptors [14] and the amyloidogenic pathway [15,16]. The clinical benefits of THA were limited by its poor oral bioavailability and considerable side effects mainly defined as hepatotoxicity. This fact caused THA withdrawal from the market [17,18]. On the other hand, 1,2,3,4-tetrahydroaminoacridine scaffold still stands at the forefront of the scientific research given by its relatively simple structure that can be easily modified to find novel, more potent, and less toxic analogues. Indeed, these endeavors yielded, for instance, 7-methoxytacrine (7-MEOTA) [14], 6-chlorotacrine (6-Cl-THA) [19], or homodimer bis(7)-tacrine [20] (Figure 1). 7-MEOTA possesses suppressed toxicity profile compared to THA due to distinct metabolic pathway preserving anti-ChE potency [14]. The chlorine atom at position 6- of the THA core strongly enhances the inhibitory activity against AChE. 6-Cl-THA has two orders of magnitude higher AChE inhibition potency than THA [21]. Bis-tacrine homodimers were developed as multifunctional analogues capable to simultaneously contact both the catalytic site (CAS) and the peripheral anionic site (PAS) of AChE [20,22]. The most active derivative is connected via seven methylene units (bis(7)-tacrine), being almost 400-fold more potent than THA in inhibiting human AChE [23]. 

Squaramides (SQ) are derivatives of squaric acid (3,4-dihydroxycyclobut-3-ene-1,2-dione) that are widely used in a variety fields of expertise (Figure 1). They mostly dominate asymmetric synthesis as chiral ligands and H-bonding catalysts [24,25,26,27], but are also valuable building blocks in medicinal chemistry. Examples of small molecules with incorporated squaramide scaffold, that reached the various stage of clinical trials, are Perzinfotel (NMDA receptor antagonist) [28], and Navarixin (chemokine receptors 1 and 2 antagonist) [29] (Figure 1). Squaramide can be considered as a motif of interest enabling formation of H-bonds bioisosteric to functionalities like carboxylic and amino acids, urea, guanidine, cyanoguanidine, and different phosphate groups [30]. More recently, mono- and bis-squaramide-based derivatives were investigated as treatment for insect-transmitted parasite diseases such as leishmaniasis [31], malaria [32], and Chagas disease [33]. 

Considering the simple synthesis and other properties (aromaticity, rigidity, H-bond formation) suitable for binding of squaramide motif, we envisaged to combine this scaffold with different tacrine-based derivatives following the successful story of bis(7)-tacrine. Bis(7)-tacrine has a broad and complex pharmacological profile. Besides highly selective inhibition potency towards AChE, it emerged as a potent inhibitor of other enzymes like β-secretase 1 (BACE-1), nitric-oxide synthase and receptors including NMDA, serotonin 5-HT_3_, gamma-aminobutyric acid GABA_A_, nicotinic and others, all of them being involved in the pathophysiology of neurodegenerative disorders [23,34,35,36]. Bis(7)-tacrine is able to inhibit self-aggregation and also AChE-induced aggregation of Aβ protein [37]. Moreover, bis(7)-tacrine has been shown to provide multiple neuroprotective effects in vitro and in vivo [38,39,40,41,42,43,44]. Bis(7)-tacrine has spurred the development of so-called multi-target directed ligands (MTDLs) targeting several pathological mechanisms pathways in AD. In this study, we developed 21 novel dimers amalgamating squaric acid scaffold with THA, 6-Cl-THA, and 7-MEOTA into so-called tacrine-squaramide homodimers (Figure 1). All new derivatives were evaluated for their anti-ChEs properties, cytotoxicity using the HepG2 cell line, and selected compounds were screened to predict their ability to cross the blood-brain barrier (BBB). In this article, we also report in silico studies of the most potent AChE and BChE inhibitors in the active site of respective enzyme. 

## 2. Results and Discussion

### 2.1. Chemistry

**Scheme 1:**
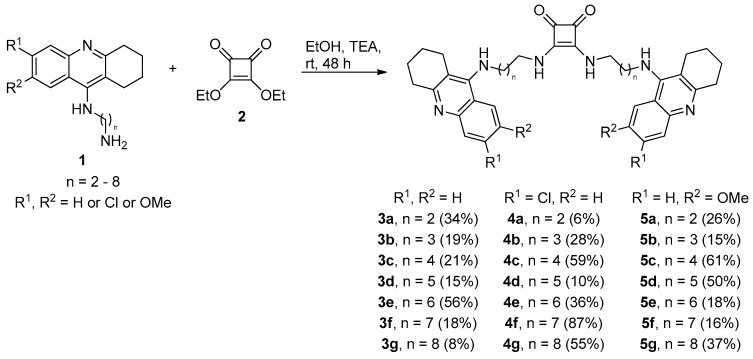
Synthetic procedure for tacrine-squaramide derivatives **3a**–**3g**, **4a**–**4g** and **5a**–**5g**.

Symmetrical squaramides are generally prepared by the reaction of dialkyloxysquarate derivatives with an excess of primary or secondary amines [30]. Aliphatic amine condensation of corresponding tacrine derivatives **1** with diethoxysquarate (**2**) in ethanol in the presence of TEA afforded desired products with 6–87% yields (THA family—**3a**–**3g**; 6-Cl-THA family—**4a**–**4g** and 7-MEOTA family—**5a**–**5g**; Scheme 1). Intermediates of general structure **1** varying in the length of alkyl chains were prepared according to previously published methods [45,46]. During the synthesis, we also observed the formation of monomeric analogues of squaramides (not shown in the Scheme 1), but these were chemically unstable when isolated and thus were excluded from further biological studies. All final compounds were characterized by ^1^H, ^13^C-NMR spectra and HRMS analysis. LC analysis confirmed their purity >95%.

### 2.2. Evaluation of Cholinesterase Inhibitory Activity

Three series of tacrine-squaramide homodimers were tested for their inhibitory potential against human AChE (*h*AChE) and human BChE (*h*BChE) enzymes using modified spectrophotometric method of Ellman et al. [47,48,49,50]. THA, 6-Cl-THA and 7-MEOTA were used as reference compounds. The IC_50_ values of all tested compounds and their selectivity index (SI) for *h*AChE are summarized in Table 1. All tested derivatives showed good to excellent inhibitory potencies for both cholinesterases with IC_50_ values ranging from micromolar to the single digit nanomolar scale. 

All homodimers bearing THA (**3a**–**g**) and 6-Cl-THA (**4a**–**g**) units displayed IC_50_ values in the nanomolar range for *h*AChE (2–72 nM). As expected, derivatives of 7-MEOTA (**5a**–**g**) were less potent, displaying IC_50_ values in the sub-micromolar to micromolar range (0.1–4.5 µM). According to *h*AChE inhibition potency, the top ranked derivative from all the derivatives under the study was **4b** (IC_50_ = 2.0 nM) being only 2.5-times less active compared to bis(7)-tacrine (IC_50_ = 0.8 nM) [51], but still one order of magnitude more potent *h*AChEI than the parent 6-Cl-THA. From THA and 7-MEOTA families, the most pronounced derivatives in terms of *h*AChE inhibition were **3a** and **5d** with IC_50_ values 3.8 and 120 nM, being 84- and 83-times more potent than references THA and 7-MEOTA, respectively. The length of the spacer between two tacrine sub-units significantly affected AChE inhibition potency. THA- and 6-Cl-THA-based dimers (compounds **3f**, **3g**, and **4f**, **4g**) with the longest chains were the least active AChEIs. Surprisingly, for the 7-MEOTA-dimers, negligible inhibitory potency was associated with derivatives bearing shorter linkers, especially to **5b** and **5c**.

Almost all homodimers were also good inhibitors of *h*BChE with IC_50_ values in the micromolar to nanomolar range. The most active *h*BChEIs were THA-based derivatives (IC_50_ = 21–75 nM) followed by 6-Cl-THA and 7-MEOTA subsets. THA-derivative **3e** exerted the highest *h*BChE inhibition potency (IC_50_ = 21 nM).

The selective/non-selective inhibition profile for ChEs in drugs potentially useful in AD treatment is largely discussed [52]. Currently used ChEIs are AChE-selective (donepezil) or possess more or less non-selective pattern of inhibition (rivastigmine, galantamine). It has been proved that levels of BChE in the brain of AD patients are elevated with age while levels of AChE are decreased. This finding underlies the importance of selective-BChEIs in the treatment of moderate-to-severe stages of AD [53]. Given this fact, only **3g** revealed small preference for *h*BChE inhibition (SI = 0.7), while most of the tacrine-squaramides were AChE-selective, highlighting **4g** from the 6-Cl-THA family with remarkable SI ˃ 10,000. In the THA-based subset, SI strongly correlated with the length of alkyl chain where the four-methylene tethered analogue (**3c**) displayed the most pronounced AChE selectivity.

### 2.3. Kinetic Study of AChE and BChE Inhibition

**Figure 2:**
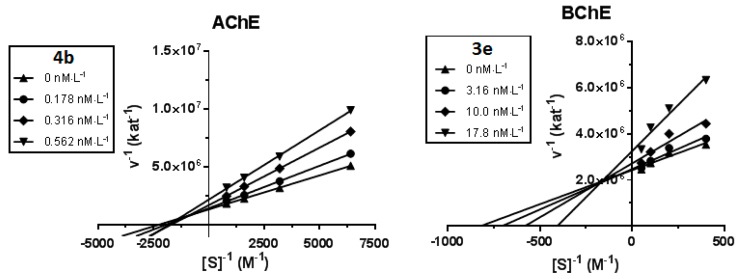
Steady state inhibition of cholinesterase substrate hydrolysis by compound **4b** (*h*AChE) and **3e** (*h*BChE) at different concentrations. Lineweaver−Burk plots of initial velocity at increasing substrate concentrations (AChE: 0.1563–1.250 mM; BChE: 2.5–20.0 mM) are presented. Lines were derived from a linear regression of the data points.

Kinetic study was performed in order to describe the interactions of the compounds **4b** and **3e** with *h*AChE and *h*BChE, respectively. Inhibition kinetics were elucidated from velocity curves that were measured at several concentrations of the corresponding substrate (AChE—acetylthiocholine, BChE—butyrylthiocholine) and tested compounds. The type of enzyme inhibition and corresponding kinetic parameters (*K*_i_ and *K*_i’_) were determined by nonlinear regression analysis. Results for each type model of inhibition (competitive, non-competitive, uncompetitive and mixed) were compared by sum-of-squares *F-*test. Statistical analysis showed mixed type of inhibition (*p* ˂ 0.05), which is in line with the Lineweaver–Burk plot, used for visualization of obtained data (Figure 2).

The intersection of lines is located above the *x*-axis for both measured inhibitors, which means reversible binding mode to both free enzyme and enzyme-substrate complex, with higher affinity to the free enzyme (*K*_i_ < *K*_i’_), interacting with its allosteric peripheral anionic site (PAS). This interaction causes conformational changes of the cholinesterase, resulting also in changes of its active site. *K*_m_ was slightly increased and *V*_max_ was reduced at higher concentration of both inhibitors. A *K*_i_ value of 0.4 ± 0.2 nM and *K*_i’_ of 0.5 ± 0.2 nM were measured for **4b** on AChE and a *K*_i_ value of 8.1 ± 1.9 nM and *K*_i’_ of 100 ± 28 nM for **3e** on BChE, respectively.

### 2.4. Cytotoxicity

The MTT (3-(4,5-dimethylthiazol-2-yl)-2,5-diphenyltetrazolium bromide) colorimetric assay performed on the human hepatocyte carcinoma (HepG2) cell line was applied to investigate the preliminary toxicity profile of all the developed derivatives [56]. The results are summarized in Table 2. The lowest cytotoxic effect displayed analogues with the shortest alkyl chains from all three series (**3a**, **4a**, and **5a**). These compounds were nontoxic at the highest tested concentrations (256 µM for **3a** and **5a**, and 128 µM for **4a**). The linker elongation negatively affected cytotoxicity in THA- and 7-MEOTA-squaramides. In other words, the most toxic hybrids were those having the longest spacers, i.e., compounds **3g** and **5g**, with IC_50_ values 6.6 and 3.5 µM, respectively. Derivatives of 6-Cl-THA were nontoxic even at the highest tested concentration of 64 µM and, intriguingly, were also less toxic than reference 6-Cl-THA (IC_50_ = 43 µM). These data have to be considered carefully since MTT cytotoxicity screening is performed on isolated cell lines, thus these results provide only preliminary toxicity insight and do not reflect the toxicity profile under in vivo conditions. For comparative purposes, we also included cytotoxicity data for other AChEIs like donepezil, galantamine and rivastigmine. Interestingly, donepezil toxicity lies in the same range as some of the least toxic tacrine-squaramides (**3c**, **4a** and **5c**), whereas rivastigmine and galantamine exerted a one to three orders lower toxic profile.

### 2.5. In Vitro BBB Permeation

An important feature for drugs targeting the brain is their ability to cross the BBB. The parallel artificial membrane permeability assay (PAMPA) is a high-throughput screening tool applicable for prediction of passive transport across BBB [57]. The results of selected homodimers and positive (THA, donepezil, ibuprofen) and negative (furosemide, chlorothiazide, ranitidine) controls are outlined in Table 3. The studied compound **4e** is likely endowed with a high probability to cross the BBB. Derivatives **3a** and **5d** with lower values of *Pe* cannot presumably cross the BBB; other tested derivatives have uncertain BBB permeation (**3e**, **4b**, and **5a**). It also has to be bear in mind, that in its current form using lipid porcine, the PAMPA-BBB assay possesses several limitations. These include mainly (i) the different composition of the human phospholipid bilayer may cause deviation in predicted permeability, (ii) omitting the absorption of compounds that are actively transported by influx transporters and pumped-out by various efflux mechanisms with the glycoprotein P at the forefront or (iii) transport through the paracellular route which is mostly exploited by small hydrophobic molecules [58]. Even considering these drawbacks, PAMPA-BBB is conceived as very simple but powerful tool for BBB prediction.

### 2.6. In Silico Studies

Molecular docking studies were carried out with the *h*AChE and *h*BChE enzymes to analyze the binding data of the developed compounds. We employed the crystal structures of *h*AChE (PDB entry: 4EY7) bound to dual anionic site ligand donepezil and *h*BChE (PDB entry: 4BDS) complexed with THA [59,60]. Both of them possess satisfactory resolution at 2.35 Å and 2.10 Å, respectively, ensuring reliable outputs. In line with the results from in vitro and following enzyme kinetic analysis, we selected **4b** and **3e**, two most pronounced *h*AChE and *h*BChE inhibitors from 6-Cl-THA and THA families, respectively. Docking analyses were carried out using Autodock Vina software (v. 1.1.2) [61].

**Figure 3:**
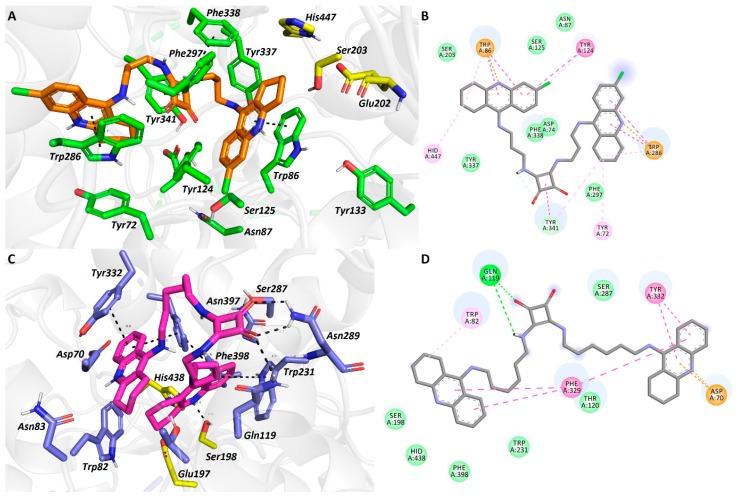
Docking results for the tacrine-squaramide hybrids **4b** and **3e** within *h*AChE (**A**,**B**) and *h*BChE (**C**,**D**) active sites, respectively (PDB IDs: 4EY7, 4BDS). A and C—Superimposed analogues **4b** and **3e** (orange and purple carbon atoms, respectively) as 3D figures; B and D—2D figures **4b** and **3e**, respectively. Generally to A and C—important amino acid residues involved in the ligand-enzyme interactions are displayed as green and blue carbon atoms, respectively; catalytic triad residues are shown in yellow, the rest of the enzyme is represented as a light-grey cartoon. Figures A and C were generated with PyMol 1.5.0.4 (The PyMOL Molecular Graphics System, Version 1.5.0.4 Schrödinger, LLC, Mannheim, Germany), Figures B and D were created with Discovery Studio 2016 Client software.

Tacrine-squaramide derivative **4b** spans the cavity gorge of *h*AChE from the bottom to the entrance (Figure 3A,B). Proximal 6-Cl-THA unit occupies CAS region by showing typical parallel π-π/cation-π interaction with Trp86 (3.9 Å; distance measured from center-to-center of aromatic rings). Chlorine atom is directed towards hydrophobic groove of the enzyme contacting Tyr124, Ser125 and Asn87. This topology orientation of 6-Cl-THA moiety has been observed more valuable in improving the inhibition potency compared to 7-MEOTA derivatives. The latter generally displays unfavorable 180° rotation of core tacrine scaffold yielding lower enzyme affinity [14,62]. Catalytic triad residues (Glu202, Ser203 and His447) are also involved in ligand stabilization revealing alkyl-π (His447) or van der Waals contacts (Glu202 and Ser203). Connecting linker is anchored via a complex web of hydrophobic interactions in the mid-gorge enzyme region. These also involve distorted π-π contacts between central squaramide scaffold with Tyr341 (4.5 Å), Tyr124 (4.5 Å), Phe297 (4.2 Å) and Phe338 (5.1 Å). The most strikingly aromatic character of squaramide and its interaction with aromatic residues in the mid-gorge region can be denoted as culprits for extraordinary inhibition potency of **4b**. At the rim of the gorge, the distal 6-Cl-THA subunit faces towards key amino acid residue from PAS region, i.e., Trp286 (parallel π-π stacking, 3.7 Å). In general, **4b**-*h*AChE complex mimics structural features in crystallographically refined bis(7)-tacrine in *Torpedo californica* AChE [63].

In the **3e**-*h*BChE complex, the ligand revealed atypical U-shaped conformation (Figure 3C,D). This can be nicely explained by the structural differences between AChE/BChE enzymes in the PAS region, where most of the aromatic amino acid residues in BChE are substituted by aliphatic ones enabling entrance of more bulky substrates [64]. From this point of view, the structural properties of BChE enabled lodging of both THA subunits of **3e** deep into cavity of *h*BChE. In this case, catalytic triad residues form either hydrophobic (His438 and Glu197) or hydrogen-bond contacts (2.5 Å). The ligand orientation is mainly orchestrated by Phe329 stabilizing both tacrine-subunits and allow ligand U-shaped conformation. Interestingly, the squaramide moiety exerted a complex web of hydrogen bonds with Asn289 (2.3 and 2.3 Å) and Gln119 (2.7 Å) at the mouth of the enzyme.

## 3. Conclusions

The never-ending quest to find effective therapy for AD is on one hand a challenging task and on the other hand, a daunting process. Enormous efforts are made to find disease-modifying agents that would improve quality of life for the AD sufferers. A wealth of scientific studies currently still indicate that ChEIs can serve as a useful tool in drug development of potential therapeutics to tackle symptomatology of AD as well their beneficial effects on pathophysiological pathways associated with the progression of the disease. Building on them, so-called MTDLs flourished as an emerging direction [65,66]. These findings, given by the drug evolution, can renew the interest in ChEIs but only if rationally designed [67]. In this study, we described the design, synthesis, and in vitro evaluation of squaramide-containing tacrine homodimers represented by THA, 6-Cl-THA, and 7-MEOTA scaffolds. All newly developed compounds were good to excellent inhibitors of both ChEs, with IC_50_ in the micromolar to single-digit nanomolar range. Compounds **4b** and **3e** were found to demonstrate the highest inhibition potencies against *h*AChE and *h*BChE, respectively. For **4b** and **3e**, the kinetic analysis revealed their mixed-type inhibition which is indicative of concurrent interaction with both anionic sites of the enzymes. The lowest cytotoxic effect on HepG2 cell line displayed analogues with the shortest alkyl chains from all three series (**3a**, **4a**, and **5a**). Increasing the number of methylene units in the linker increases the cytotoxicity. Interestingly, this finding does not apply to the 6-Cl-THA family. However, from the six selected squaramide-tacrine homodimers, only **4e** is predicted to be BBB permeable. In silico studies predicted orientation of **4b** in *h*AChE active site correlating well with previously published crystallographic structure of bis(7)-tacrine in AChE. Compound **3e** displayed rather atypical U-shaped conformation in the *h*BChE active site, given presumably by the topological properties of cholinesterase. With this in mind, we believe that squaramides can be further recognized as interesting building blocks in the drug discovery with particular emphasis on AD.

## 4. Experimental Section

### 4.1. General Chemistry Methods

All chemical solvents and reagents were used in the highest available purity without further purification and they were purchased from Sigma-Aldrich (Prague, Czech Republic). The reactions were monitored by thin layer chromatography (TLC) on silica gel plates (60 F254, Merck, Prague, Czech Republic) and the spots were visualized by ultraviolet light (254 nm). Purification of crude products was carried out using columns of silica gel (silica gel 100, 0.063–0.200 mm, 70–230 mesh ASTM, Fluka, Prague, Czech Republic). NMR spectra were recorded in deuterated chloroform (CDCl_3_), deuterated methanol (CD_3_OD) and deuterated dimethyl sulfoxide (DMSO-*d*_6_) on a Varian S500 spectrometer. Chemical shifts (*δ*) are reported in parts per millions (ppm) and spin multiplicities are given as bold singlet (bs), doublet (d), doublet of doublet (dd), triplet (t), quartet (q), pentet (p), or multiplet (m). Coupling constants (*J*) are reported in Hz. Recorded NMR data are available at Supplementary Information. The synthesized compounds were analyzed by an LC-MS system consisting of UHLPC Dionex Ultimate 3000 couplet with Q Exaxtive Plus mass spectrometer to obtain high resolution mass spectra (Thermo Fisher Scientific, Bremen, Germany). Melting points were measured using an automated melting point recorder M-565 (Büchi, Switzerland). The final compounds were analyzed by LC-MS consisting of UHPLC Dionex Ultimate 3000 RS coupled with Q Exactive Plus orbitrap mass spectrometer (Thermo Fisher Scientific, Bremen, Germany) to obtain high-resolution mass spectra. Gradient LC analysis confirmed > 95% purity.

#### General Procedure for the Preparation of Tacrine-Squaramides (**3a**–**3g**; **4a**–**4g** and **5a**–**5g**)

To the solution of diethoxysquarate 2 (1.0 eq) in EtOH (1 M) were added corresponding tacrine derivative (2.0 eq) and TEA (2.0 eq). The reaction mixture was stirred at room temperature for 48 h. After completion of the reaction, the solvent was evaporated and crude product was purified by column chromatography using a mixture of eluents (DCM:MeOH:NH_3_ = 20:1:0.1).

*Bis({2-[(1,2,3,4-tetrahydroacridin-9-yl)amino]ethyl}amino)cyclobut-3-ene-1,2-dione* (**3a**). Yield 34%; yellow solid; Mp = 159–160 °C; ^1^H-NMR (500 MHz, CD_3_OD): *δ* 8.06–7.99 (m, 2H), 7.74–7.69 (m, 2H), 7.54–7.47 (m, 2H), 7.36–7.28 (m, 2H), 3.79–3.60 (m, 8H), 2.95–2.88 (m, 4H), 2.72–2.61 (m, 4H), 1.88–1.75 (m, 8H); ^13^C-NMR (126 MHz, CD_3_OD): *δ* 183.6 (CO), 158.7, 153.2, 146.8, 137.8, 130.3, 127.2, 125.3, 124.2, 121.1, 117.6, 46.1, 33.6, 26.1, 23.8, 23.4; HRMS [M + H]^+^: 561.2972; (calculated for [C_34_H_36_N_6_O_2_]^+^: 561.2973).

*Bis({3-[(1,2,3,4-tetrahydroacridin-9-yl)amino]propyl}amino)cyclobut-3-ene-1,2-dione* (**3b**). Yield 19%; yellow solid; Mp = 196–198 °C; ^1^H-NMR (500 MHz, DMSO-*d*_6_): *δ* 8.20–8.10 (m, 2H), 7.74–7.66 (m, 2H), 7.60–7.50 (m, 2H), 7.39–7.30 (m, 2H), 3.65–3.44 (m, 8H), 2.92–2.85 (m, 4H), 2.72–2.62 (m, 4H), 1.90–1.56 (m, 12H); ^13^C-NMR (126 MHz, DMSO-*d*_6_): *δ* 182.5, 159.2, 151.6, 147.3, 132.8, 126.5, 125.5, 123.7, 118.5, 116.1, 48.8, 44.9, 41.1, 33.5, 32.3, 25.2, 22.7, 22.3; HRMS [M + H]^+^: 589.3218 (calculated for [C_36_H_40_N_6_O_2_]^+^: 589.3286).

*Bis({4-[(1,2,3,4-tetrahydroacridin-9-yl)amino]butyl}amino)cyclobut-3-ene-1,2-dione* (**3c**). Yield 21%; yellow solid; Mp = 47–49 °C; ^1^H-NMR (500 MHz, CD_3_OD): *δ* 8.11–8.06 (m, 2H), 7.76–7.71 (m, 2H), 7.57–7.51 (m, 2H), 7.38–7.33 (m, 2H), 3.61–3.55 (m, 4H), 2.98–2.92 (m, 4H), 2.74–2.67 (m, 4H), 1.87 (dd, *J* = 7.3, 4.4 Hz, 8H), 1.69–1.63 (m, 8H), 1.31–1.24 (m, 4H); ^13^C-NMR (126 MHz, CD_3_OD): *δ* 183.5, 169.3, 158.5, 153.4, 147.0, 130.2, 127.2, 125.0, 124.5, 120.9, 116.7, 79.5, 60.2, 44.8, 33.7, 29.7, 28.9, 26.1, 23.9, 23.5, 8.5; HRMS [M + H]^+^: 617.3603 (calculated for [C_38_H_44_N_6_O_2_]^+^: 617.3599).

*Bis({5-[(1,2,3,4-tetrahydroacridin-9-yl)amino]pentyl}amino)cyclobut-3-ene-1,2-dione* (**3d**). Yield 15%; brown solid; Mp = 76–77 °C; ^1^H-NMR (500 MHz, CDCl_3_): *δ* 7.98–7.92 (m, 2H), 7.86–7.81 (m, 2H), 7.54–7.47 (m, 2H), 7.34–7.28 (m, 2H), 4.44–4.37 (m, 2H), 3.81 (bs, 5H), 3.63 (t, *J* = 7.0 Hz, 4H), 3.45 (q, *J* = 6.8 Hz, 4H), 3.03–2.96 (m, 4H), 2.66–2.60 (m, 4H), 1.89–1.83 (m, 8H), 1.67–1.60 (m, 8H), 1.47–1.36 (m, 4H); ^13^C-NMR (126 MHz, CDCl_3_): *δ* 182.2, 168.1, 157.3, 151.3, 146.1, 128.7, 126.9, 123.6, 123.0, 119.5, 115.2, 50.0, 48.8, 43.9, 32.9, 30.8, 30.6, 24.5, 23.5, 22.7, 22.3; HRMS [M + H]^+^: 645.3903 (calculated for [C_40_H_48_N_6_O_2_]^+^: 645.3912).

*Bis({6-[(1,2,3,4-tetrahydroacridin-9-yl)amino]hexyl}amino)cyclobut-3-ene-1,2-dione* (**3e**). Yield 56%; yellow solid; Mp = 93–95 °C; ^1^H-NMR (500 MHz, CDCl_3_): *δ* 7.90 (dd, *J* = 8.6, 1.4 Hz, 2H), 7.81 (dd, *J* = 8.5, 1.2 Hz, 2H), 7.66 (bs, 2H), 7.51–7.44 (m, 2H), 7.32–7.25 (m, 2H), 4.14 (t, *J* = 6.0 Hz, 2H), 3.62–3.53 (m, 4H), 3.39 (q, *J* = 6.7 Hz, 4H), 3.10 (bs, 2H), 3.02–2.91 (m, 4H), 2.67–2.55 (m, 4H), 1.91–1.78 (m, 8H), 1.60–1.46 (m, 8H), 1.34–1.19 (m, 8H); ^13^C-NMR (126 MHz, CDCl_3_): *δ* 182.4, 168.1, 157.9, 151.1, 146.9, 128.5, 127.8, 123.6, 122.9, 119.8, 115.5, 50.4, 49.0, 44.1, 33.6, 31.4, 30.9, 26.3, 25.9, 24.7, 22.9, 22.6; HRMS [M + H]^+^: 673.4218 (calculated for [C_42_H_52_N_6_O_2_]^+^: 673.4225).

*Bis({7-[(1,2,3,4-tetrahydroacridin-9-yl)amino]heptyl}amino)cyclobut-3-ene-1,2-dione* (**3f**). Yield 18%; yellow solid; Mp = 64–66 °C; ^1^H-NMR (500 MHz, CD_3_OD): *δ* 8.11 (d, *J* = 8.6 Hz, 2H), 7.75–7.70 (m, 2H), 7.61–7.55 (m, 2H), 7.40–7.34 (m, 2H), 3.61–3.53 (m, 8H), 3.00–2.92 (m, 4H), 2.72–2.66 (m, 4H), 1.94–1.84 (m, 8H), 1.68–1.61 (m, 4H), 1.58–1.51 (m, 4H), 1.38–1.29 (m, 12H); ^13^C-NMR (126 MHz, CD_3_OD): *δ* 183.5, 169.4, 157.4, 154.2, 146.1, 130.7, 126.2, 125.0, 124.9, 120.3, 115.8, 45.1, 33.1, 32.1, 32.0, 29.8, 27.7, 27.2, 25.9, 23.8, 23.3; HRMS [M + H]^+^: 701.4534 (calculated for [C_44_H_56_N_6_O_2_]^+^: 701.4538).

*Bis({8-[(1,2,3,4-tetrahydroacridin-9-yl)amino]octyl}amino)cyclobut-3-ene-1,2-dione* (**3g**). Yield 8%; yellow solid; Mp = 64–65 °C; ^1^H-NMR (500 MHz, CDCl_3_): *δ* 7.97 (d, *J* = 8.5 Hz, 2H), 7.91–7.78 (m, 4H), 7.51 (t, *J* = 7.6 Hz, 2H), 7.32 (t, *J* = 7.6 Hz, 2H), 4.34 (t, *J* = 6.0 Hz, 2H), 3.73–3.35 (m, 12H), 3.03–2.95 (m, 4H), 2.72–2.61 (m, 4H), 1.96–1.78 (m, 8H), 1.63–1.47 (m, 8H), 1.35–1.09 (m, 12H); ^13^C-NMR (126 MHz, CDCl_3_): *δ* 182.4, 168.2, 157.5, 151.6, 146.5, 128.8, 127.3, 123.7, 123.2, 119.6, 115.2, 50.4, 48.9, 44.3, 33.3, 31.5, 30.9, 28.8, 26.4, 26.1, 24.6, 22.8, 22.5; HRMS [M + H]^+^: 729.4848 (calculated for [C_46_H_60_N_6_O_2_]^+^: 729.4851).

*Bis({2-[(6-chloro-1,2,3,4-tetrahydroacridin-9-yl)amino]ethyl}amino)cyclobut-3-ene-1,2-dione* (**4a**). Yield 6%; yellow solid; Mp = 113–114 °C; ^1^H-NMR (500 MHz, DMSO-*d*_6_): *δ* 8.10 (d, *J* = 9.1 Hz, 2H), 7.72–7.66 (m, 2H), 7.35–7.24 (m, 2H), 3.73–3.43 (m, 8H), 2.91–2.75 (m, 4H), 2.70–2.59 (m, 4H), 1.80–1.67 (m, 8H); ^13^C-NMR (126 MHz, DMSO-*d*_6_): *δ* 182.6, 159.4, 150.5, 147.4, 132.7, 126.6, 125.5, 123.8, 118.8, 116.8, 69.9, 48.8, 44.3, 33.5, 25.1, 22.7, 22.3; HRMS [M + H]^+^: 629.2179 (calculated for [C_34_H_34_Cl_2_N_6_O_2_]^+^: 629.2194).

*Bis({3-[(6-chloro-1,2,3,4-tetrahydroacridin-9-yl)amino]propyl}amino)cyclobut-3-ene-1,2-dione* (**4b**). Yield 28%; Mp = 86–88 °C; ^1^H-NMR (500 MHz, DMSO-*d*_6_): *δ* 8.11 (d, *J* = 9.1 Hz, 2H), 7.70–7.65 (m, 2H), 7.42 (bs, 2H), 7.32–7.25 (m, 2H), 3.52 (bs, 4H), 3.49–3.41 (m, 8H), 2.90–2.81 (m, 4H), 2.70–2.60 (m, 4H), 1.86–1.69 (m, 12H); ^13^C-NMR (126 MHz, DMSO-*d*_6_): *δ* 182.5, 159.2, 150.6, 147.3, 132.8, 126.5, 125.5, 123.67, 118.5, 116.1, 48.8, 44.9, 41.1, 33.5, 32.3, 25.2, 22.7, 22.3; HRMS [M + H]^+^: 657.2513 (calculated for [C_36_H_38_Cl_2_N_6_O_2_]^+^: 657.2507).

*Bis({4-[(6-chloro-1,2,3,4-tetrahydroacridin-9-yl)amino]butyl}amino)cyclobut-3-ene-1,2-dione* (**4c**). Yield 59%; yellow solid; Mp = 99–100 °C; ^1^H-NMR (500 MHz, DMSO-*d*_6_): *δ* 8.13 (d, *J* = 9.1 Hz, 2H), 7.69 (d, *J* = 2.2 Hz, 2H), 7.42 (bs, 2H), 7.32 (dd, *J* = 9.0, 2.3 Hz, 2H), 3.64–3.27 (m, 8H), 2.90–2.82 (m, 4H), 2.68–2.60 (m, 4H), 1.84–1.66 (m, 8H), 1.60–1.46 (m, 8H); ^13^C-NMR (126 MHz, DMSO-*d*_6_): *δ* 182.4, 167.9, 158.8, 150.9, 146.9, 133.0, 126.1, 125.7, 123.8, 118.4, 115.9, 47.6, 43.1, 33.2, 28.3, 27.6, 25.1, 22.6, 22.2; HRMS [M + H]^+^: 685.2819 (calculated for [C_38_H_42_Cl_2_N_6_O_2_]^+^: 685.2820).

*Bis({5-[(6-chloro-1,2,3,4-tetrahydroacridin-9-yl)amino]pentyl}amino)cyclobut-3-ene-1,2-dione* (**4d**). Yield 10%; yellow solid; Mp = 85–86 °C; ^1^H-NMR (500 MHz, DMSO-*d*_6_): *δ* 8.11 (d, *J* = 9.1 Hz, 2H), 7.69 (d, *J* = 2.3 Hz, 2H), 7.31 (dd, *J* = 9.1, 2.3 Hz, 2H), 3.42–3.24 (m, 8H), 2.92–2.81 (m, 4H), 2.71–2.60 (m, 4H), 1.84–1.69 (m, 8H), 1.62–1.52 (m, 4H), 1.51–1.40 (m, 4H), 1.35–1.23 (m, 4H); ^13^C-NMR (126 MHz, DMSO-*d*_6_): *δ* 182.4, 167.9, 159.4, 150.7, 147.6, 132.6, 126.7, 125.6, 123.6, 118.7, 116.1, 47.9, 43.3, 33.6, 30.6, 30.3, 25.2, 23.4, 22.7, 22.4; HRMS [M + H]^+^: 713.3129 (calculated for [C_40_H_46_Cl_2_N_6_O_2_]^+^: 713.3133).

*Bis({6-[(6-chloro-1,2,3,4-tetrahydroacridin-9-yl)amino]hexyl}amino)cyclobut-3-ene-1,2-dione* (**4e**). Yield 36%; yellow solid; Mp = 87–88 °C; ^1^H-NMR (500 MHz, DMSO-*d*_6_): *δ* 8.11 (d, *J* = 9.1 Hz, 2H), 7.69 (d, *J* = 2.3 Hz, 2H), 7.31 (dd, *J* = 9.1, 2.3 Hz, 2H), 5.55 (t, *J* = 6.3 Hz, 2H), 4.10 (bs, 2H), 3.44–3.35 (m, 8H), 2.86 (t, *J* = 6.2 Hz, 4H), 2.65 (t, *J* = 6.0 Hz, 4H), 1.83–1.71 (m, 8H), 1.57–1.49 (m, 4H), 1.48–1.40 (m, 4H), 1.31–1.20 (m, 8H); ^13^C-NMR (126 MHz, DMSO-*d*_6_): *δ* 182.4, 167.9, 159.3, 150.7, 147.5, 132.7, 126.7, 125.6, 123.6, 118.7, 115.9, 48.0, 43.3, 33.6, 30.9, 30.6, 26.1, 25.7, 25.2, 22.7, 22.4; HRMS [M + H]^+^: 741.3434 (calculated for [C_42_H_50_Cl_2_N_6_O_2_]^+^: 741.3446).

*Bis({7-[(6-chloro-1,2,3,4-tetrahydroacridin-9-yl)amino]heptyl}amino)cyclobut-3-ene-1,2-dione* (**4f**). Yield 87%; yellow solid; Mp = 85–86 °C; ^1^H-NMR (500 MHz, CDCl_3_): *δ* 7.85 (d, *J* = 9.1 Hz, 2H), 7.79 (d, *J* = 2.2 Hz, 2H), 7.54 (bs, 2H), 7.21 (dd, *J* = 9.0, 2.2 Hz, 2H), 4.12 (t, *J* = 6.0 Hz, 2H), 3.59 (q, *J* = 6.7 Hz, 4H), 3.43 (q, *J* = 6.7 Hz, 4H), 3.01–2.90 (m, 4H), 2.66–2.55 (m, 4H), 1.92–1.81 (m, 8H), 1.61–1.43 (m, 8H), 1.31–1.16 (m, 12H); ^13^C-NMR (126 MHz, CDCl_3_): *δ* 182.4, 168.1, 159.3, 151.1, 147.7, 134.1, 126.9, 124.8, 124.1, 118.2, 115.5, 50.6, 49.0, 44.4, 33.8, 31.4, 30.9, 28.6, 26.4, 26.2, 24.5, 22.8, 22.5; HRMS [M + H]^+^: 769.3745 (calculated for [C_44_H_54_Cl_2_N_6_O_2_]^+^: 769.8446).

*Bis({8-[(6-chloro-1,2,3,4-tetrahydroacridin-9-yl)amino]octyl}amino)cyclobut-3-ene-1,2-dione* (**4g**). Yield 55%; brown solid; Mp = 58–60 °C; ^1^H-NMR (500 MHz, CDCl_3_): *δ* 7.87 (d, *J* = 9.0 Hz, 2H), 7.80 (d, *J* = 2.1 Hz, 2H), 7.60 (bs, 2H), 7.23 (dd, *J* = 9.0, 2.2 Hz, 2H), 4.13 (t, *J* = 6.0 Hz, 2H), 3.62 (q, *J* = 6.7 Hz, 4H), 3.47 (q, *J* = 6.7 Hz, 4H), 3.01–2.92 (m, 4H), 2.67–2.59 (m, 4H), 1.93–1.83 (m, 8H), 1.59 (p, *J* = 7.1 Hz, 4H), 1.51 (p, *J* = 7.0 Hz, 4H), 1.29–1.09 (m, 16H); ^13^C-NMR (126 MHz, CDCl_3_): *δ* 190.0, 189.8, 184.6, 184.4, 178.0, 177.4, 174.8, 174.7, 160.2, 160.2, 153.3, 148.5, 135.5, 126.6, 126.6, 126.5, 124.9, 119.4, 116.7, 70.6, 45.5, 45.3, 34.2, 32.2, 32.2, 31.8, 31.5, 30.2, 30.1, 30.1, 29.9, 27.8, 27.7, 27.3, 27.2, 25.9, 25.9, 23.9, 23.6, 16.2, 16.1; HRMS [M + H]^+^: 797.4061 (calculated for [C_44_H_54_Cl_2_N_6_O_2_]^+^: 797.4072).

*Bis({2-[(7-methoxy-1,2,3,4-tetrahydroacridin-9-yl)amino]ethyl}amino)cyclobut-3-ene-1,2-dione* (**5a**). Yield 26%; yellow solid; Mp = 106–107 °C; ^1^H-NMR (500 MHz, DMSO-*d*_6_): *δ* 7.63 (d, *J* = 9.1 Hz, 2H), 7.51 (bs, 2H), 7.41–7.35 (m, 2H), 7.18 (dd, *J* = 9.1, 2.7 Hz, 2H), 5.36 (bs, 2H), 3.84 (s, 6H), 3.74–3.57 (m, 4H), 3.54–3.40 (m, 4H), 2.90–2.80 (m, 4H), 2.77–2.66 (m, 4H), 1.86–1.64 (m, 8H); ^13^C-NMR (126 MHz, DMSO-*d*_6_): *δ* 182.6, 168.1, 155.8, 155.6, 149.3, 142.4, 129.7, 121.5, 120.3, 117.9, 101.6, 55.5, 48.8, 48.4, 44.2, 33.2, 25.4, 22.9, 22.6; HRMS [M + H]^+^: 621.3178 (calculated for [C_36_H_40_N_6_O_4_]^+^: 621.3184).

*Bis({3-[(7-methoxy-1,2,3,4-tetrahydroacridin-9-yl)amino]propyl}amino)cyclobut-3-ene-1,2-dione* (**5b**). Yield 15%; yellow solid; Mp = 64–65 °C; ^1^H-NMR (500 MHz, DMSO-*d*_6_): *δ* 7.63 (d, *J* = 9.1 Hz, 2H), 7.42 (d, *J* = 2.6 Hz, 2H), 7.19 (dd, *J* = 9.1, 2.6 Hz, 2H), 5.48 (bs, 2H), 3.85 (s, 6H), 3.55 (bs, 4H), 3.42–3.31 (m, 8H), 2.86 (t, *J* = 6.5 Hz, 4H), 2.70 (t, *J* = 6.3 Hz, 4H), 1.85–1.69 (m, 12H); ^13^C-NMR (126 MHz, DMSO-*d*_6_): *δ* 182.5, 167.9, 155.8, 155.5, 149.6, 142.3, 130.2, 129.6, 123.6, 121.3, 120.3, 118.8, 117.2, 101.6, 55.6, 44.5, 41.2, 33.1, 32.4, 25.6, 22.9, 22.6; HRMS [M + H]^+^: 649.3488 (calculated for [C_38_H_44_N_6_O_4_]^+^: 649.3497).

*Bis({4-[(7-methoxy-1,2,3,4-tetrahydroacridin-9-yl)amino]butyl}amino)cyclobut-3-ene-1,2-dione* (**5c**). Yield 61%; yellow solid; Mp = 91–92 °C; ^1^H-NMR (500 MHz, CDCl_3_): *δ* 7.84 (bs, 2H), 7.71 (d, *J* = 9.1 Hz, 2H), 7.18 (d, *J* = 2.7 Hz, 2H), 7.15 (d, *J* = 2.6 Hz, 1H), 7.14 (d, *J* = 2.6 Hz, 1H), 4.23–4.12 (m, 2H), 3.81 (s, 6H), 3.59–3.54 (m, 4H), 3.40–3.23 (m, 4H), 2.96–2.88 (m, 4H), 2.65–2.55 (m, 4H), 1.86–1.73 (m, 8H), 1.69–1.55 (m, 8H); ^13^C-NMR (126 MHz, CD_3_OD): *δ* 183.5, 169.3, 157.7, 156.4, 152.4, 142.8, 128.9, 122.2, 118.0, 102.7, 56.1, 44.9, 33.6, 29.8, 28.9, 26.3, 24.0, 23.6; HRMS [M + H]^+^: 677.3799 (calculated for [C_40_H_48_N_6_O_4_]^+^: 677.3810).

*Bis({5-[(7-methoxy-1,2,3,4-tetrahydroacridin-9-yl)amino]pentyl}amino)cyclobut-3-ene-1,2-dione* (**5d**). Yield 50%; yellow solid; Mp = 70–72 °C; ^1^H-NMR (500 MHz, CDCl_3_): *δ* 7.85 (s, 2H), 7.73 (d, *J* = 9.1 Hz, 2H), 7.21 (d, *J* = 2.7 Hz, 2H), 7.17 (dd, *J* = 9.1, 2.6 Hz, 2H), 4.29–4.13 (m, 2H), 3.84 (s, 6H), 3.64–3.59 (m, 4H), 3.35 (q, *J* = 6.8 Hz, 4H), 2.94 (m, *J* = 6.8 Hz, 4H), 2.66–2.61 (m, 4H), 1.85–1.81 (m, 8H), 1.62–1.56 (m, 8H), 1.44–1.35 (m, 4H); ^13^C-NMR (126 MHz, CDCl_3_): *δ* 182.4, 168.2, 156.0, 155.3, 150.6, 141.8, 128.5, 120.8, 120.8, 116.6, 101.9, 55.6, 48.4, 44.0, 32.9, 31.0, 30.7, 24.7, 23.7, 22.8, 22.5; HRMS [M + H]^+^: 705.4119 (calculated for [C_42_H_52_N_6_O_4_]^+^: 705.4123).

*Bis({6-[(7-methoxy-1,2,3,4-tetrahydroacridin-9-yl)amino]hexyl}amino)cyclobut-3-ene-1,2-dione* (**5e**). Yield 18%; yellow solid; Mp = 157–159 °C; ^1^H-NMR (500 MHz, CDCl_3_): *δ* 8.20 (bs, 2H), 7.75 (d, *J* = 9.2 Hz, 2H), 7.32 (d, *J* = 2.7 Hz, 2H), 7.18 (dd, *J* = 9.2, 2.6 Hz, 2H), 4.83 (bs, 2H), 4.20–3.93 (m, 4H), 3.87 (s, 6H), 3.67–3.60 (m, 4H), 2.99–2.94 (m, 4H), 2.70–2.65 (m, 4H), 1.88–1.81 (m, 8H), 1.69–1.55 (m, 10H), 1.47–1.32 (m, 10H); ^13^C-NMR (126 MHz, CDCl_3_): *δ* 182.2, 168.3, 156.2, 154.1, 151.5, 140.2, 127.1, 121.4, 120.1, 115.4, 102.1, 55.7, 50.5, 48.1, 44.0, 32.0, 31.1, 30.7, 26.1, 25.8, 24.8, 22.7, 22.1; HRMS [M + H]^+^: 733.4434 (calculated for [C_44_H_56_N_6_O_4_]^+^: 733.4436).

*Bis({7-[(7-methoxy-1,2,3,4-tetrahydroacridin-9-yl)amino]heptyl}amino)cyclobut-3-ene-1,2-dione* (**5f**). Yield 16%; yellow solid; Mp = 132–134 °C; ^1^H-NMR (500 MHz, CDCl_3_): *δ* 8.32 (bs, 2H), 7.81 (dd, *J* = 9.5, 4.7 Hz, 2H), 7.36 (d, *J* = 2.6 Hz, 2H), 7.26–7.17 (m, 2H), 3.88 (s, 4H), 3.68–3.58 (m, 4H), 3.58–3.48 (m, 4H), 3.05–2.95 (m, 4H), 2.75–2.62 (m, 4H), 1.92–1.80 (m, 8H), 1.72–1.50 (m, 8H), 1.38–1.29 (m, 12H); ^13^C-NMR (126 MHz, CDCl_3_): *δ* 182.2, 168.3, 156.2, 153.5, 152.0, 139.5, 126.5, 121.7, 119.8, 114.9, 102.4, 55.7, 53.4, 48.2, 44.2, 31.6, 31.1, 30.7, 28.5, 26.4, 25.9, 24.7, 22.7, 21.9; HRMS [M + H]^+^: 761.4736 (calculated for [C_46_H_60_N_6_O_4_]^+^: 761.4749).

*Bis({8-[(7-methoxy-1,2,3,4-tetrahydroacridin-9-yl)amino]octyl}amino)cyclobut-3-ene-1,2-dione* (**5g**). Yield 37%; yellow solid; Mp = 87–89 °C; ^1^H-NMR (50 MHz, CDCl_3_): *δ* 7.78–7.72 (m, 2H), 7.65 (bs, 2H), 7.25–7.19 (m, 4H), 3.95 (t, *J* = 6.4 Hz, 2H), 3.88 (s, 6H), 3.61 (q, *J* = 6.5 Hz, 4H), 3.43 (q, *J* = 6.7 Hz, 4H), 3.03–2.91 (m, 4H), 2.73–2.67 (m, 4H), 2.61 (bs, 2H), 1.93–1.83 (m, 8H), 1.58 (p, *J* = 7.0 Hz, 4H), 1.47 (m, 4H), 1.27–1.06 (m, 16H); ^13^C-NMR (126 MHz, CDCl_3_): *δ* 182.6, 168.2, 155.9, 155.9, 150.6, 142.9, 129.3, 120.9, 120.6, 116.8, 101.9, 55.5, 50.5, 48.4, 44.3, 33.5, 31.8, 30.7, 28.8, 28.8, 26.4, 25.9, 24.7, 22.9, 22.7; HRMS [M + H]^+^: 789.5064 (calculated for [C_48_H_64_N_6_O_4_]^+^: 789.5062).

### 4.2. In Vitro Anti-ChE Assay

The AChE/BChE inhibitory activity of the tested drugs was determined using Ellman’s method [47,48,49,50] and is expressed as IC_50_, the i.e., concentration that reduces the cholinesterase activity by 50%. Human plasmatic butyrylcholinesterase (BChE; EC 3.1.1.8) and recombinant acetylcholinesterase (AChE; 3.1.1.7) were prepared at the Department of Toxicology and Military Pharmacy. 5,5′-dithiobis(2-nitrobenzoic acid) (Ellman’s reagent, DTNB), phosphate buffer (PB, pH 7.4), acetylthiocholine (ATC) and butyrylthiocholine (BTC), were purchased from Sigma-Aldrich, Prague, Czech Republic. For measuring purposes – polystyrene Nunc 96-well microplates with flat bottom shape (ThermoFisher Scientific, USA) were utilized.

All the assays were carried out in 0.1 M KH_2_PO_4_/K_2_HPO_4_ buffer, pH 7.4. Enzyme solutions were prepared at 2.0 units/mL in 2 mL aliquots. The assay medium (100 µL) consisted of 40 µL of 0.1 M phosphate buffer (pH 7.4), 20 µL of 0.01 M DTNB, 10 µL of the enzyme, and 20 µL of 0.01 M substrate (ATC/BTC iodide solution).

Inhibitor solutions in concentration range 10^−3^–10^−11^ M were prepared and IC_50_ values were calculated. Tested compounds were preincubated for 5 min. The reaction was started by immediate addition of 20 µL of the substrate. The activity was determined by measuring the increase in absorbance at 412 for AChE/BChE at 37 °C at 2 min intervals - using a Multi-mode microplate reader Synergy 2 (Winooski, VT, USA). Each concentration was assayed in triplicate. Software GraphPad Prizm 5 (San Diego, CA, USA) was used for the statistical data evaluation.

### 4.3. Kinetic Study of AChE and BChE Inhibition

The kinetic study of AChE and BChE was performed by using the above-mentioned modified Ellman’s method. The values of *V*_max_ and *K*_m_ of the Michaelis-Menten kinetics as well as the values of *K*_i_ and *K*_i’_ were calculated by nonlinear regression from the substrate velocity curves. Linear regression was used for calculation of Lineweaver-Burk plots. All calculations were performed using GraphPad Prism software version 6.07 for Windows (San Diego, CA, USA).

### 4.4. Evaluation of Cytotoxicity by MTT Assay

The human origin cell line HepG2 (ATCC, Mannassas, VA, USA) isolated from liver hepatocellular carcinoma was used to evaluate cytotoxicity of tested compounds. The cells were cultivated in Dublecco’s modified Eagle’s medium (DMEM; Biosera, Nuaille, France) supplemented with 10% fetal bovine serum (Biosera) and 1% penicillin-streptomycin antibiotic solution (Sigma-Aldrich, St. Louis, MO, USA), incubated at 37 °C in a CO_2_ incubator (Binder CO_2_ incubator BC 160, Tuttlingen, Germany) and routinely passaged by trypsinization at 75–85% confluence.

The MTT (3-(4,5-dimethylthiazol-2-yl)-2,5-diphenyl-tetraziolium bromide (Sigma–Aldrich) reduction assay was used for measurement of compounds’ cytotoxicity according to [68]. MTT is a water soluble tetrazolium salt and it is converted to purple formazan by succinate dehydrogenase in mitochondria of viable cells [69,70]. Cell viability was detected after 24-h incubation with the tested substances. For the assay, HepG2 cells were seeded into 96-well plates in 100 µL volume and density of 15 × 10^3^ per well. Cells were allowed to attach overnight before the treatment. The stock solutions of tested compounds were prepared in dimethyl sulfoxide (DMSO; Sigma-Aldrich), which were further serially diluted in DMEM and added to the cells in 96-well culture plates. The final concentration of DMSO was less than 0.25% per well.

After 24-h incubation, the medium containing serially diluted substances was aspirated from each well and replaced by 100 µL of fresh medium containing MTT (0.5 mg/mL). Plates were subsequently incubated at 37 °C in CO_2_ incubator for 45 min. The medium containing MTT was then aspirated and formazan dissolved in 100 μL of DMSO. The optical density of each well was measured using Synergy 2 Multi-Mode Microplate Reader (BioTek Instruments, Inc., Winooski, VT, USA) at 570 nm. The cell viability was expressed as the percentage of untreated control. Each experiment was performed in triplicate and repeated three independent times.

The IC_50_ values were calculated using four parametric nonlinear regression by statistic GraphPad Prism software (version 5.04; GraphPad Software Inc., San Diego, CA) from the logarithmic dose–response curve. The IC_50_ values were expressed as a mean ± standard error of the mean (SEM).

### 4.5. Determination of in Vitro BBB Permeation

PAMPA (the parallel artificial membrane permeability assay) is a high-throughput screening tool applicable for prediction of the passive transport of potential drugs across the blood-brain barrier (BBB) [57]. In this study, it has been used as a non-cell-based in vitro assay carried out in a coated 96-well membrane filter. The filter membrane of the donor plate was coated with PBL (Polar Brain Lipid, Avanti, USA) in dodecane (4 µl of 20 mg/mL PBL in dodecane) and the acceptor well was filled with 300 µl of phosphate buffer saline, (PBS pH 7.4; ***V_A_***). The tested compounds were dissolved first in DMSO and then diluted with PBS pH 7.4 to reach the final concentrations 50–500 µM in the donor well. The final concentration of DMSO did not exceed 0.5% (*v*/*v*) in the donor solution; 300 µl of the donor solution (***V_D_***) was added to the donor wells and the donor filter plate was carefully put on the acceptor plate so that the coated membrane was “in touch” with both donor solution and acceptor buffer. In principle, test compound diffuse from the donor well through the polar brain lipid membrane (area = 0.28 cm^2^) to the acceptor well. The concentration of the tested compound in both donor and the acceptor wells were assessed after 3, 4, 5 and 6 h of incubation in quadruplicate using the UV plate reader Synergy HT (Biotek, USA) at the maximum absorption wavelength of each compound (*n* = 3). Besides that, the solution of theoretical compound concentration, simulating the equilibrium state, established if the membrane was ideally permeable was prepared and assessed as well. The concentration of the compounds in the donor and acceptor well and equilibrium concentration were calculated from the standard curve and expressed as the permeability (***Pe***) according the equation [57]

(1) $$\log Pe=\log\left\{C\times -\ln\left(1-\frac{{\left[drug\right]}_{acceptor}}{{\left[drug\right]}_{equilibrium}}\right)\right\}$$

where $C=\left(\frac{V_{D}\times V_{A}}{\left(V_{D}+V_{A}\right)\times Area\times Time}\right)$.

### 4.6. Molecular Modeling Studies

From the online PDB database (www.pdb.org) models of *h*AChE (PDB ID: 4EY7, resolution: 2.35 Å) and *h*BChE (PDB ID: 4BDS, resolution: 2.10 Å) were downloaded and prepared for flexible molecular docking by MGL Tools utilities [59,60]. The preparation of these receptors involved removal of the surplus copies of the enzyme chains, non-bonded inhibitors, addition of polar hydrogens and merging of non-polar ones. Default Gasteiger charges were assigned to all atoms. Flexible parts of the enzymes were determined by a spherical selection of residues (R = 11 Å) approximately around the center of the active site. In the same points, the centers of the grid box of 33 × 33 × 33 Å were positioned. The rotatable bonds in the flexible residues were detected automatically by AutoDock Tools 1.5.4 program. Given the limitation of the program used for flexible molecular docking, water molecules had to be removed from the system. The flexible receptor parts contained 40 residues for *h*AChE and 39 residues for *h*BChE. The following xyz coordinates of the grid box centers were applied: *h*AChE (10.698, −58.115, −23.192); *h*BChE (140.117, 122.247, 38.986). The studied ligands were firstly drawn in HyperChem 8.0, then manually protonated as suggested by MarvinSketch 6.2.0. software (http://www.chemaxon.com), geometrically optimized by semi-empirical quantum-chemistry PM3 method and stored as pdb files. The structures of the ligands were processed for docking in a similar way as abovementioned flexible parts of the receptor by AutoDock Tools 1.5.4 program. Molecular docking was carried out in AutoDock Vina 1.1.2 program utilizing computer resources of the Czech National Grid Infrastructure MetaCentrum. The search algorithm of AutoDock Vina efficiently combines a Markov chain Monte Carlo like method for the global search and a Broyden-Fletcher-Goldfarb-Shano gradient approach for the local search [61]. It is a type of memetic algorithm based on interleaving stochastic and deterministic calculations [70]. Each docking task was repeated 20 times with the exhaustiveness parameter set to 16, employing 16 CPUs in parallel multithreading. From the obtained results, the solutions reaching the minimum predicted Gibbs binding energy were taken as the top-scoring modes. The graphic representations of the docked poses were shown in PyMOL (The PyMOL Molecular Graphics System, Version 1.5.0.4 Schrödinger, LLC.). 2D diagrams were generated using Discovery Studio Visualizer v16.1.0.15350 (Dassault Systèmes Biovia Corp., 2016, San Diego, CA, USA).

## Figures and Tables

**Table 1:** *h*AChE and *h*BChE inhibitory activities of tacrine-squaramide homodimers and reference compounds THA, 6-Cl-THA and 7-MEOTA.

Compound	R^1^	R^2^	n	IC_50_ (nM) ^a^		SI ^b^
				*h*AChE	*h*BChE	
**3a**	H	H	2	3.8	62	16
**3b**	H	H	3	8.4	55	6.5
**3c**	H	H	4	5.5	75	14
**3d**	H	H	5	8.1	69	8.5
**3e**	H	H	6	13	21	1.6
**3f**	H	H	7	32	32	1
**3g**	H	H	8	72	50	0.7
**4a**	Cl	H	2	4.2	150	36
**4b**	Cl	H	3	2.0	110	55
**4c**	Cl	H	4	4.6	170	37
**4d**	Cl	H	5	8.2	310	38
**4e**	Cl	H	6	3.2	540	170
**4f**	Cl	H	7	16	450	28
**4g**	Cl	H	8	10	˃100,000	˃10,000
**5a**	H	OMe	2	170	130	0.7
**5b**	H	OMe	3	1100	8900	8.1
**5c**	H	OMe	4	4500	4700	0.9
**5d**	H	OMe	5	120	680	5.7
**5e**	H	OMe	6	150	1100	7.3
**5f**	H	OMe	7	170	1300	7.6
**5g**	H	OMe	8	490	2900	5.9
THA ^c^	H	H	-	320	80	0.3
6-Cl-THA ^c^	Cl	H	-	20	1800	90
7-MEOTA ^c^	H	OMe	-	10,000	17,000	1.7

^a^ Results are expressed as the mean of at least three experiments; ^b^ SI = selectivity index, selectivity for *h*AChE is determined as ratio IC_50_(*h*BChE)/IC_50_(*h*AChE); ^c^ data taken from Ref. [45,46,54,55].

**Table 2:** Cytotoxicity of tacrine-squaramides evaluated using HepG2 cells measured by colorimetric MTT assay.

Compound	R^1^	R^2^	n	IC_50_ (µM) ^a^
**3a**	H	H	2	>256
**3b**	H	H	3	>32
**3c**	H	H	4	150
**3d**	H	H	5	69
**3e**	H	H	6	76
**3f**	H	H	7	23
**3g**	H	H	8	6.6
**4a**	Cl	H	2	>128
**4b**	Cl	H	3	>64
**4c**	Cl	H	4	>64
**4d**	Cl	H	5	>64
**4e**	Cl	H	6	>64
**4f**	Cl	H	7	>64
**4g**	Cl	H	8	>64
**5a**	H	OMe	2	>256
**5b**	H	OMe	3	>64
**5c**	H	OMe	4	190
**5d**	H	OMe	5	98
**5e**	H	OMe	6	26
**5f**	H	OMe	7	9
**5g**	H	OMe	8	3.5
THA	H	H	-	169
6-Cl-THA	Cl	H	-	43
7-MEOTA	H	OMe	-	44
Donepezil	-	-	-	150
Rivastigmine	-	-	-	3400
Galantamine	-	-	-	4200

^a^ The IC_50_ values are expressed as the mean of at least three independent experiments.

**Table 3:** Prediction of BBB penetration of the studied compounds expressed as *Pe* values.

Compound	R^1^	R^2^	n	*Pe* (× 10^−6^ cm.s^−1^) ^a^	CNS (+/−) ^b^
**3a**	H	H	3	1.1	CNS −
**3e**	H	H	3	2.6	CNS +/−
**4b**	Cl	H	3	2.5	CNS +/−
**4e**	Cl	H	6	17.0	CNS +
**5a**	H	OMe	2	2.3	CNS +/−
**5d**	H	OMe	5	1.3	CNS −
THA	H	H	-	6.0	CNS +
donepezil				21.0	CNS +
ibuprofen				12.0	CNS +
furosemide				0.0	CNS −
chlorothiazide				0.3	CNS −
ranitidine				0.0	CNS −

^a^ Results are expressed as the mean of at least three experiments; ^b^ CNS +: high BBB permeation predicted with *Pe* (10^−6^ cm·s^−1^) > 4.0, CNS −: low BBB permeation predicted with *Pe* (10^−6^ cm·s^−1^) < 2.0, CNS +/−: BBB permeation uncertain with *Pe* (10^−6^ cm·s^−1^) from 4.0 to 2.0.

## Supplementary Materials

The data containing ^1^H and ^13^C NMR spectra are available online at https://www.mdpi.com/2218-273X/9/8/379/s1.
