# Supplementary material for: Exploring Structure-Activity Relationship in Tacrine-Squaramide Derivatives as Potent Cholinesterase Inhibitors
Source: Biomolecules. 2019 Aug 19;9(8):379. doi: 10.3390/biom9080379 (PMC6723352; doi:10.3390/biom9080379)

Exploring Structure-Activity Relationship in Tacrine-Squaramide Derivatives as Potent Cholinesterase Inhibitors

Barbora Svobodova,[1,2,#] Eva Mezeiova,[1,2,3#] Vendula Hepnarova,[1,2] Martina Hrabinova,[1,2] Lubica Muckova,[1,2] Tereza Kobrlova,[1] Tomas Kucera,[1] Daniel Jun,[1,2] Ondrej Soukup,[1,2] María L. Jimeno,[4] José Marco-Contelles,[3,*] and Jan Korabecny[1,2,*]

[1] Department of Toxicology and Military Pharmacy, Faculty of Military Health Sciences, Trebesska 1575, 500 01 Hradec Kralove, Czech Republic
[2] Biomedical Research Centre, University Hospital Hradec Kralove, Sokolska 581, 500 05 Hradec Kralove, Czech Republic
[3] Laboratory of Medicinal Chemistry, Institute of General Organic chemistry, Juan de la Cierva 3, 28006-Madrid, Spain
[4] Centro de Química Orgánica "Lora-Tamayo" (CSIC), C/ Juan de la Cierva 3, 28006-Madrid, Spain

[#]Author contribution: B.S. and E.M. contributed equally.

*Corresponding author: J.K - Phone: +420 973 255 167; e-mail: jan.korabecny@fnhk.cz; E.M. – Phone: +420 973 255 167; e-mail: eva.mezeiova@gmail.com

^{1}H and ^{13}C spectral copies of final products

Bis({2-[(1,2,3,4-tetrahydroacridin-9-yl)amino]ethyl}amino)cyclobut-3-ene-1,2-dione (3a) ^{1}H NMR:

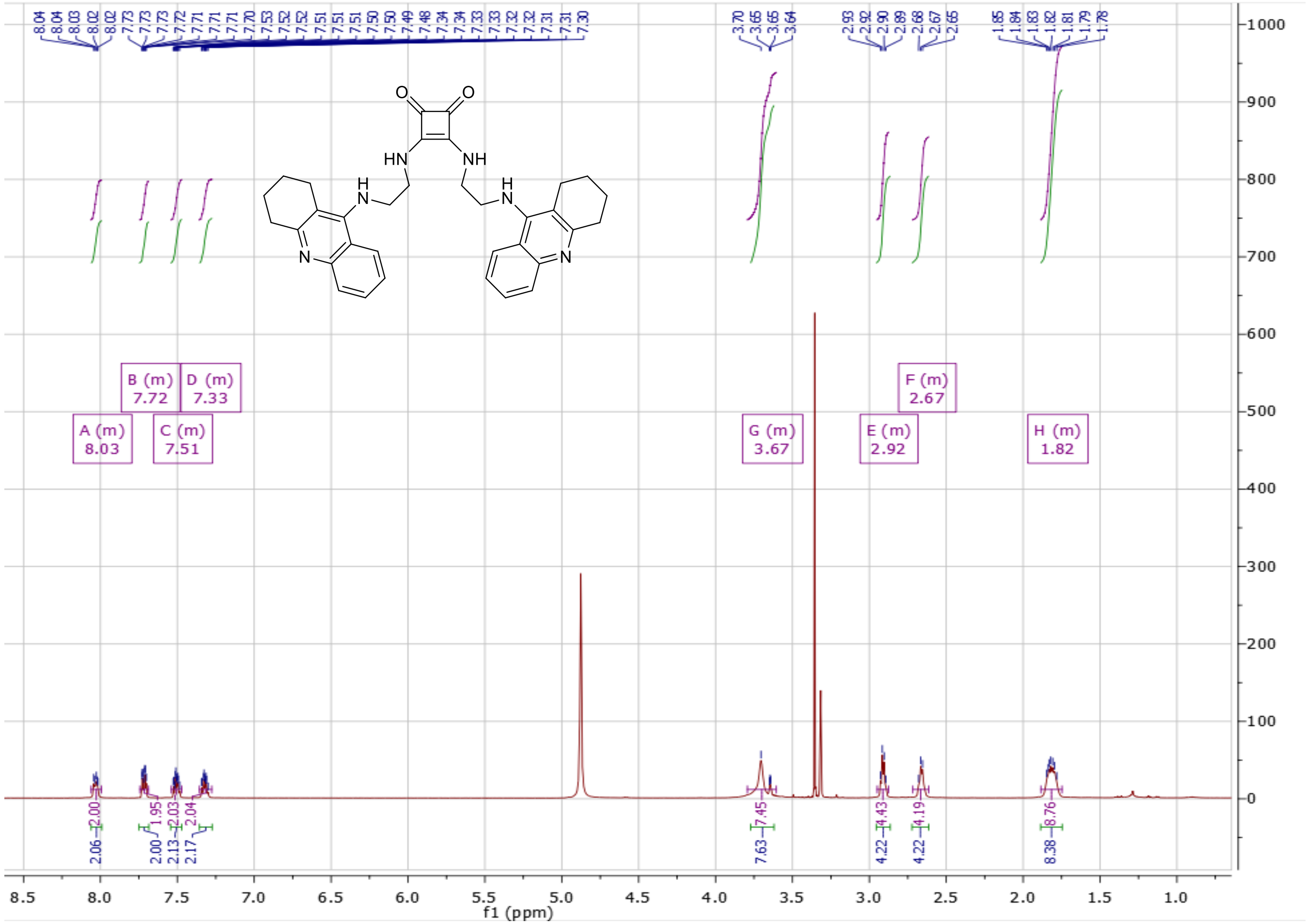

Bis({2-[(1,2,3,4-tetrahydroacridin-9-yl)amino]ethyl}amino)cyclobut-3-ene-1,2-dione (3a) ^{13}C NMR:

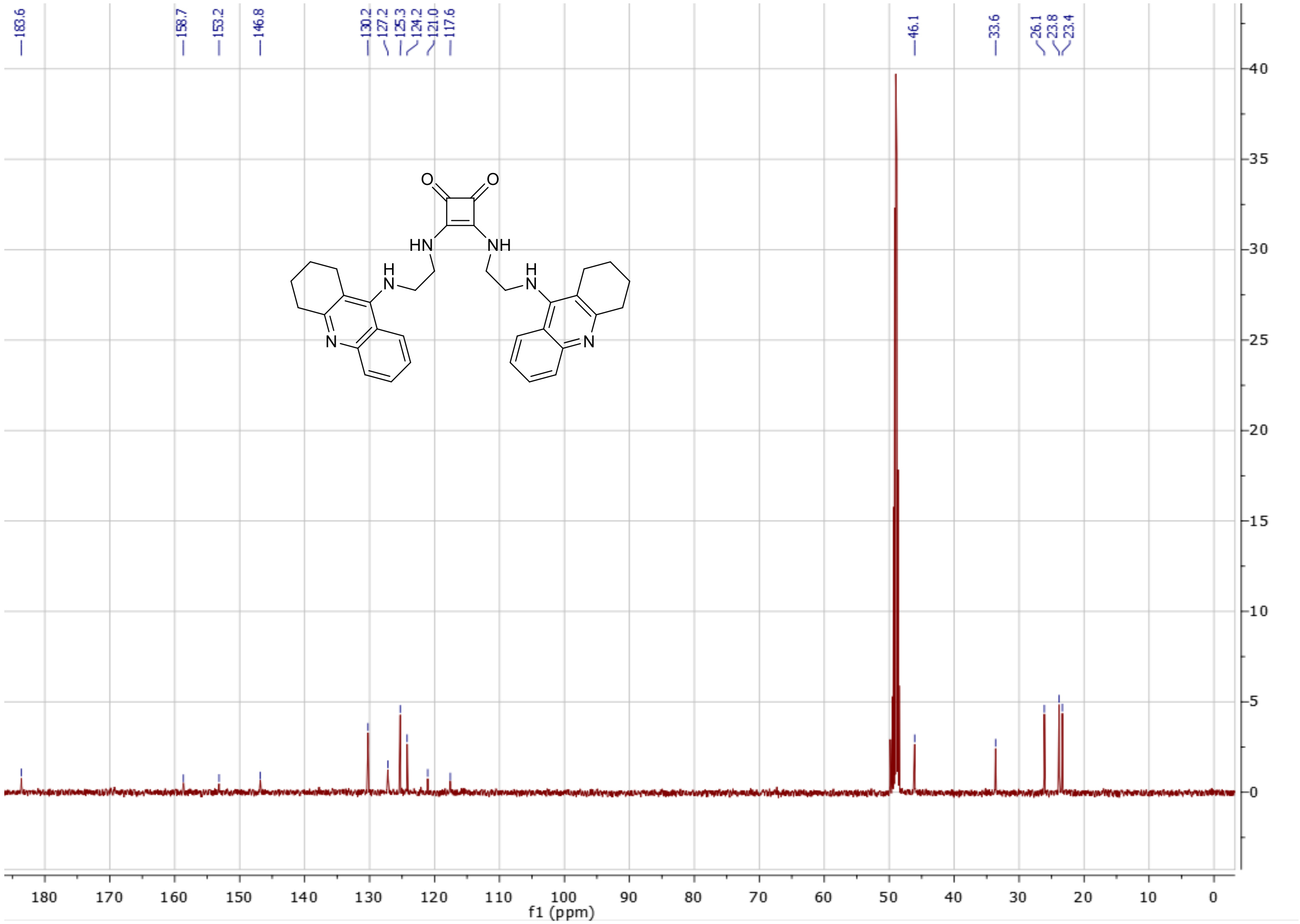

Bis({3-[(1,2,3,4-tetrahydroacridin-9-yl)amino]propyl}amino)cyclobut-3-ene-1,2-dione (3b) ^{1}H NMR:

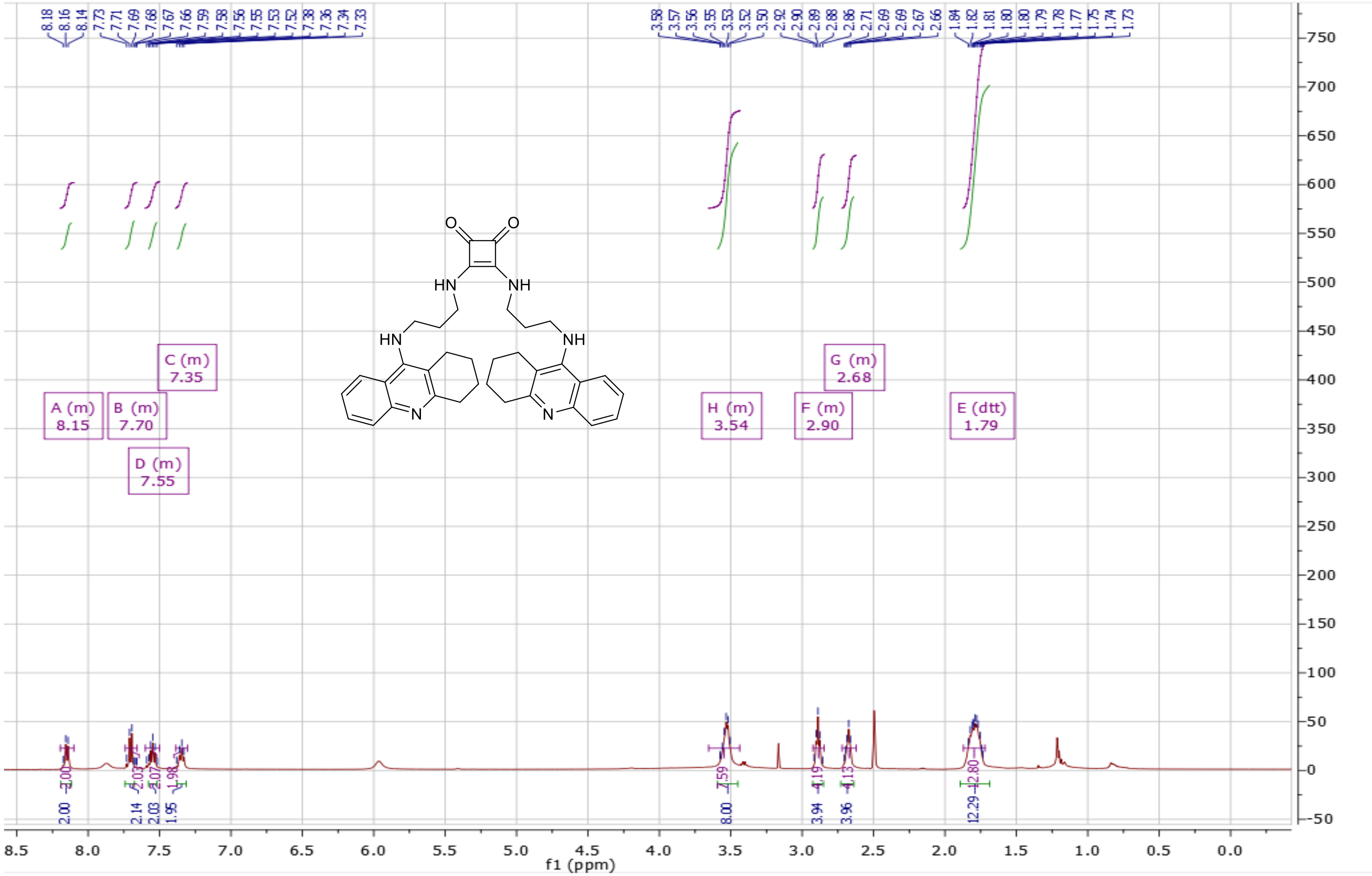

Bis({3-[(1,2,3,4-tetrahydroacridin-9-yl)amino]propyl}amino)cyclobut-3-ene-1,2-dione (3b) ^{13}C NMR:

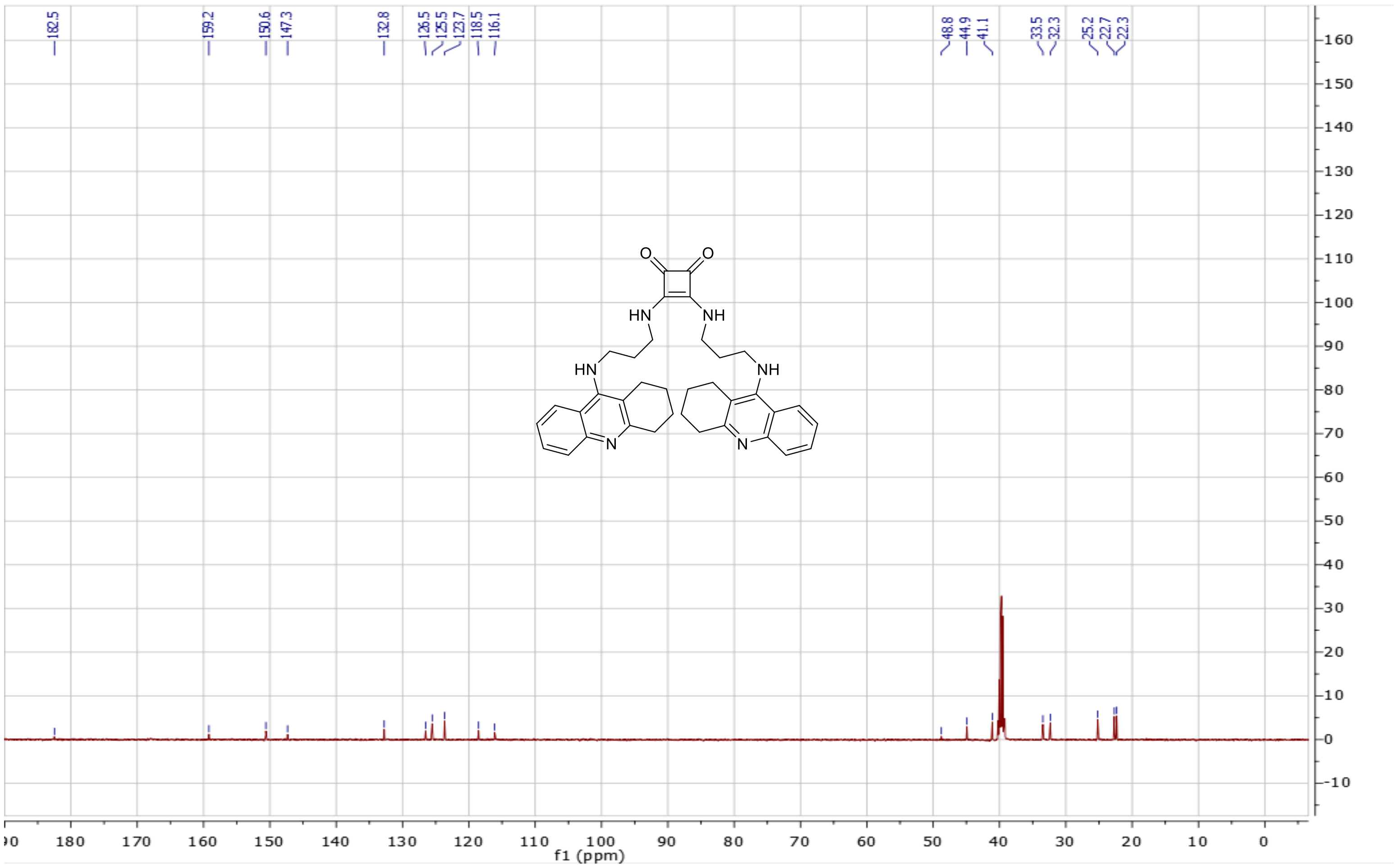

Bis({4-[(1,2,3,4-tetrahydroacridin-9-yl)amino]butyl}amino)cyclobut-3-ene-1,2-dione (3c) ¹H NMR:

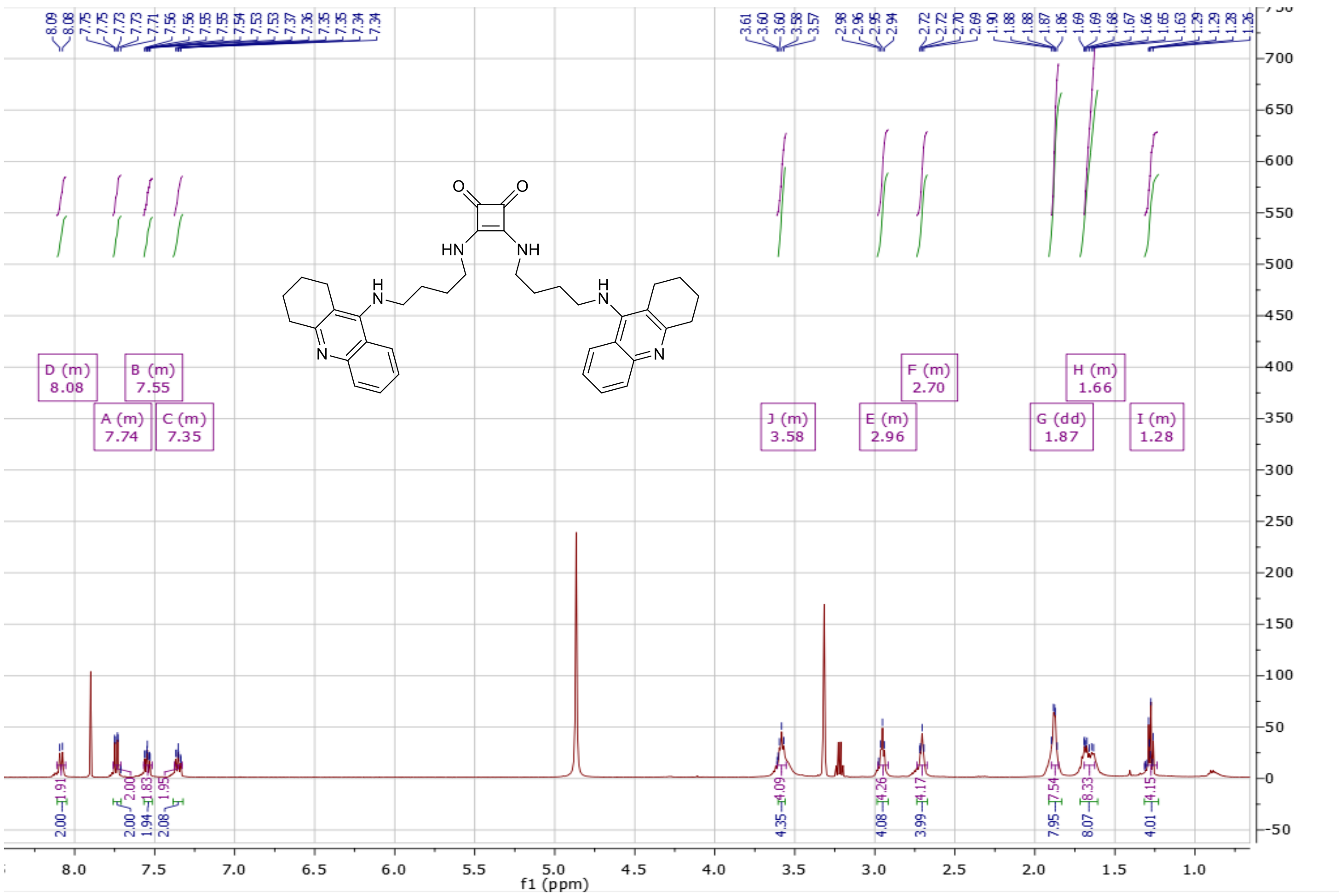

Bis({4-[(1,2,3,4-tetrahydroacridin-9-yl)amino]butyl}amino)cyclobut-3-ene-1,2-dione (3c) ^{13}C NMR:

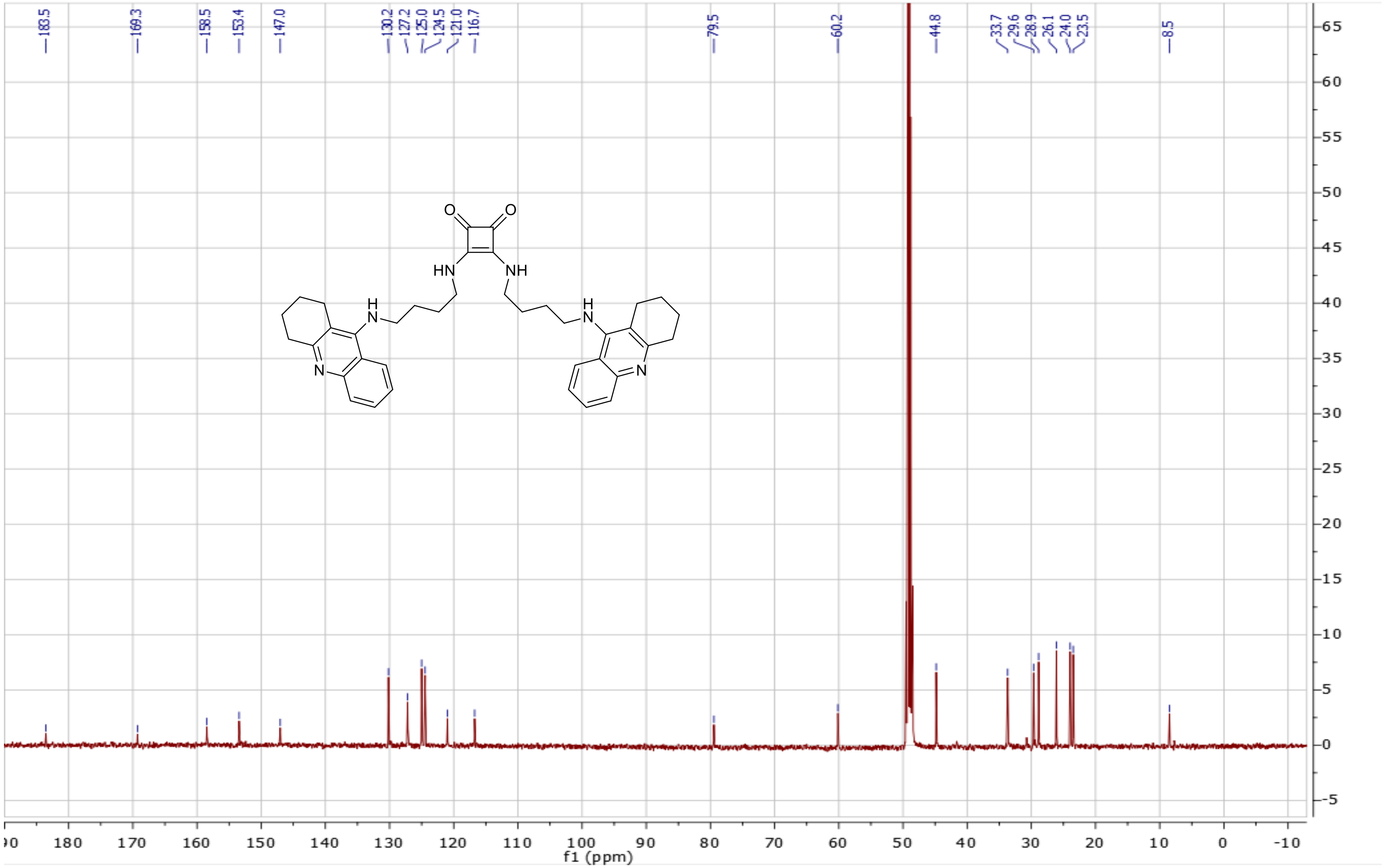

Bis({5-[(1,2,3,4-tetrahydroacridin-9-yl)amino]pentyl}amino)cyclobut-3-ene-1,2-dione (3d) ^{1}H NMR:

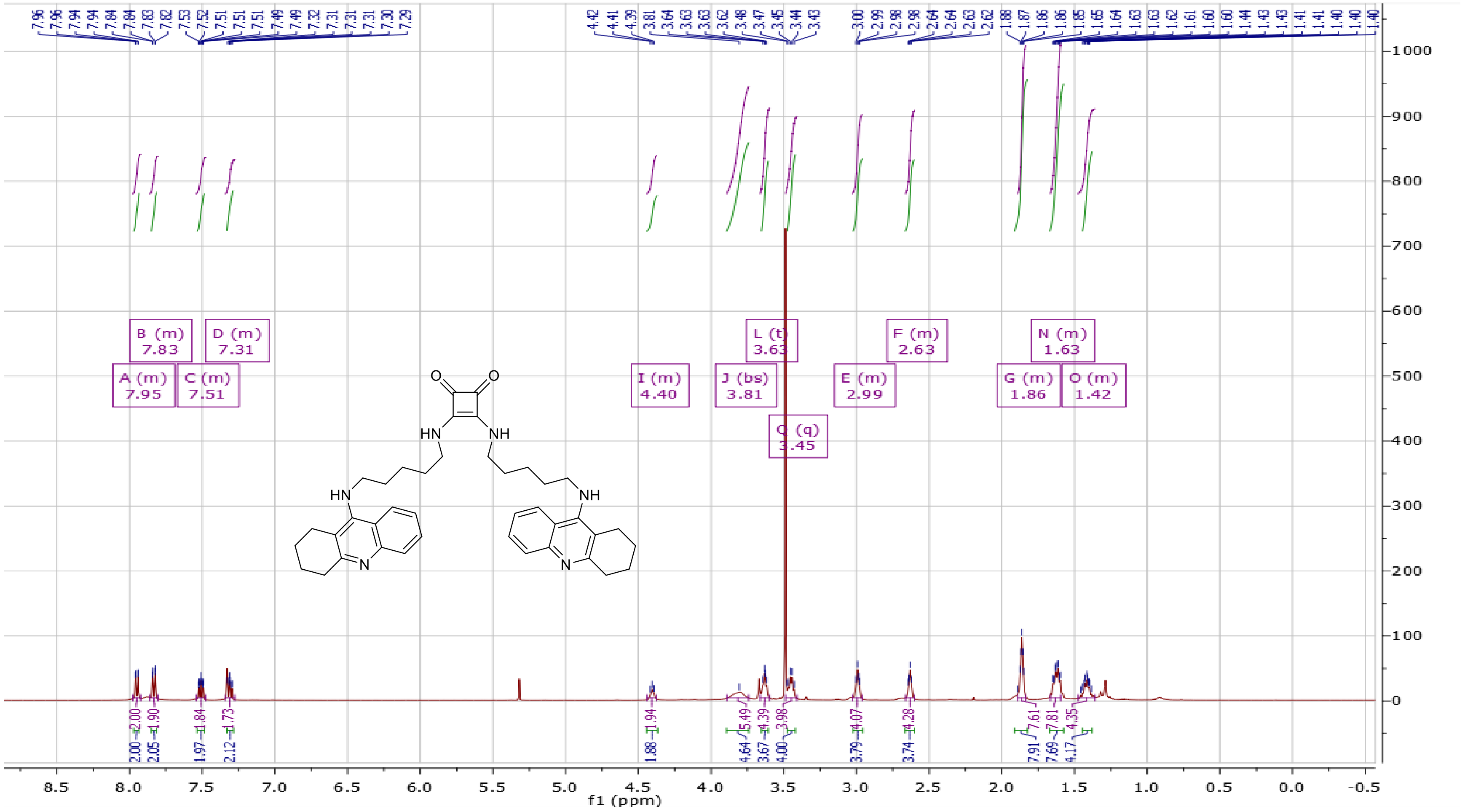

Bis({5-[(1,2,3,4-tetrahydroacridin-9-yl)amino]pentyl}amino)cyclobut-3-ene-1,2-dione (3d) ^{13}C NMR:

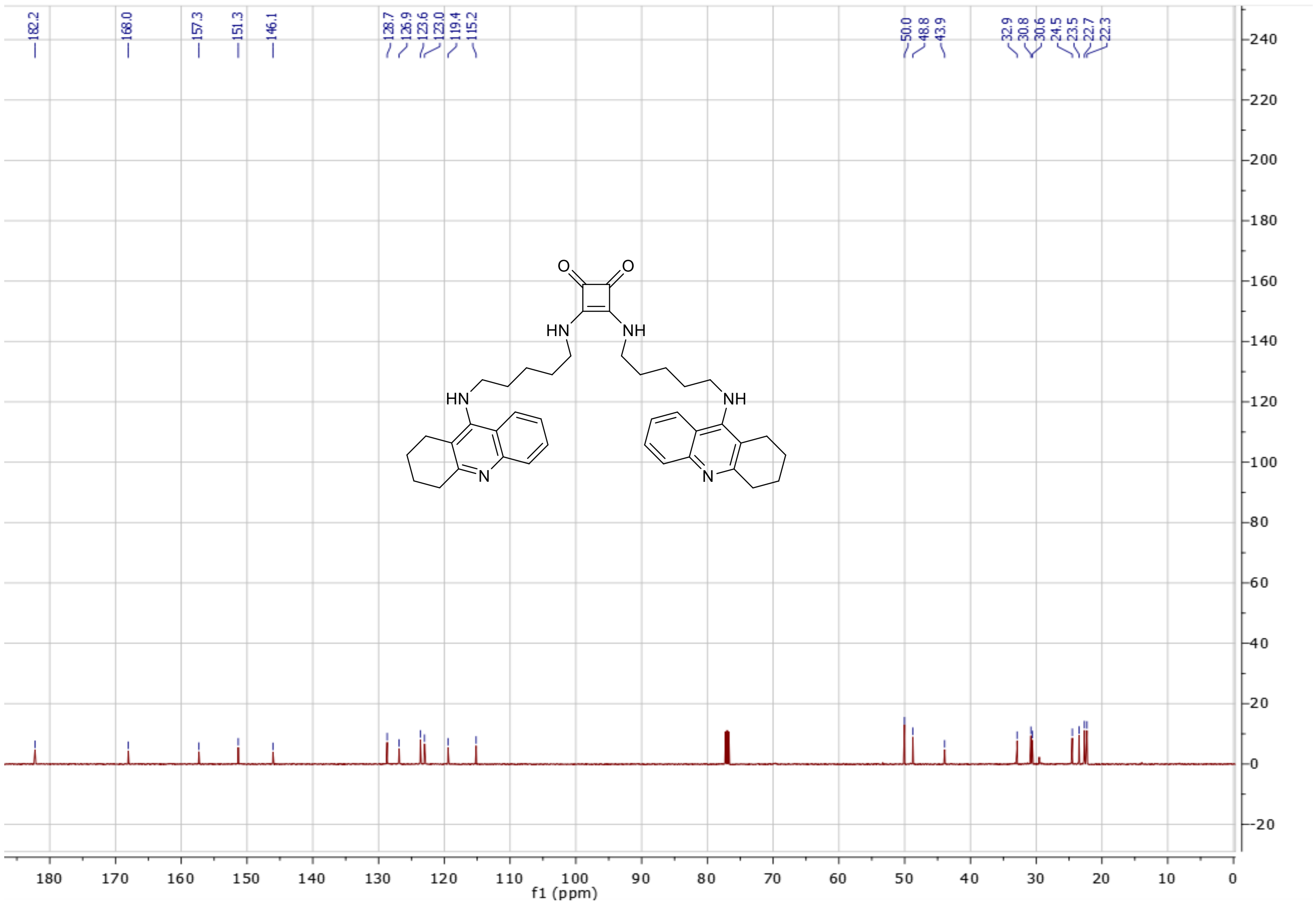

Bis({6-[(1,2,3,4-tetrahydroacridin-9-yl)amino]hexyl}amino)cyclobut-3-ene-1,2-dione (3e) ^{1}H NMR:

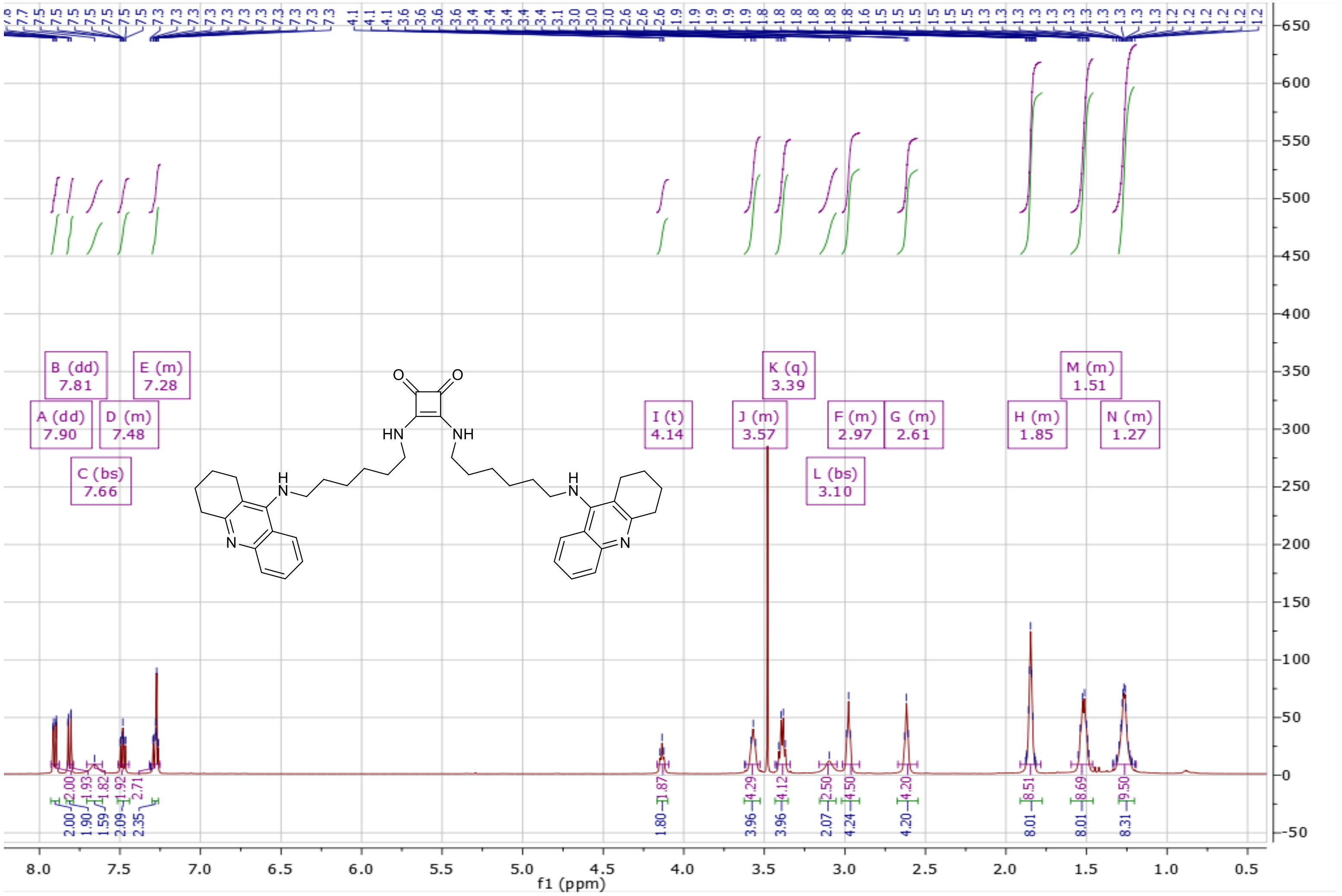

Bis({6-[(1,2,3,4-tetrahydroacridin-9-yl)amino]hexyl}amino)cyclobut-3-ene-1,2-dione (3e) ^{13}C NMR:

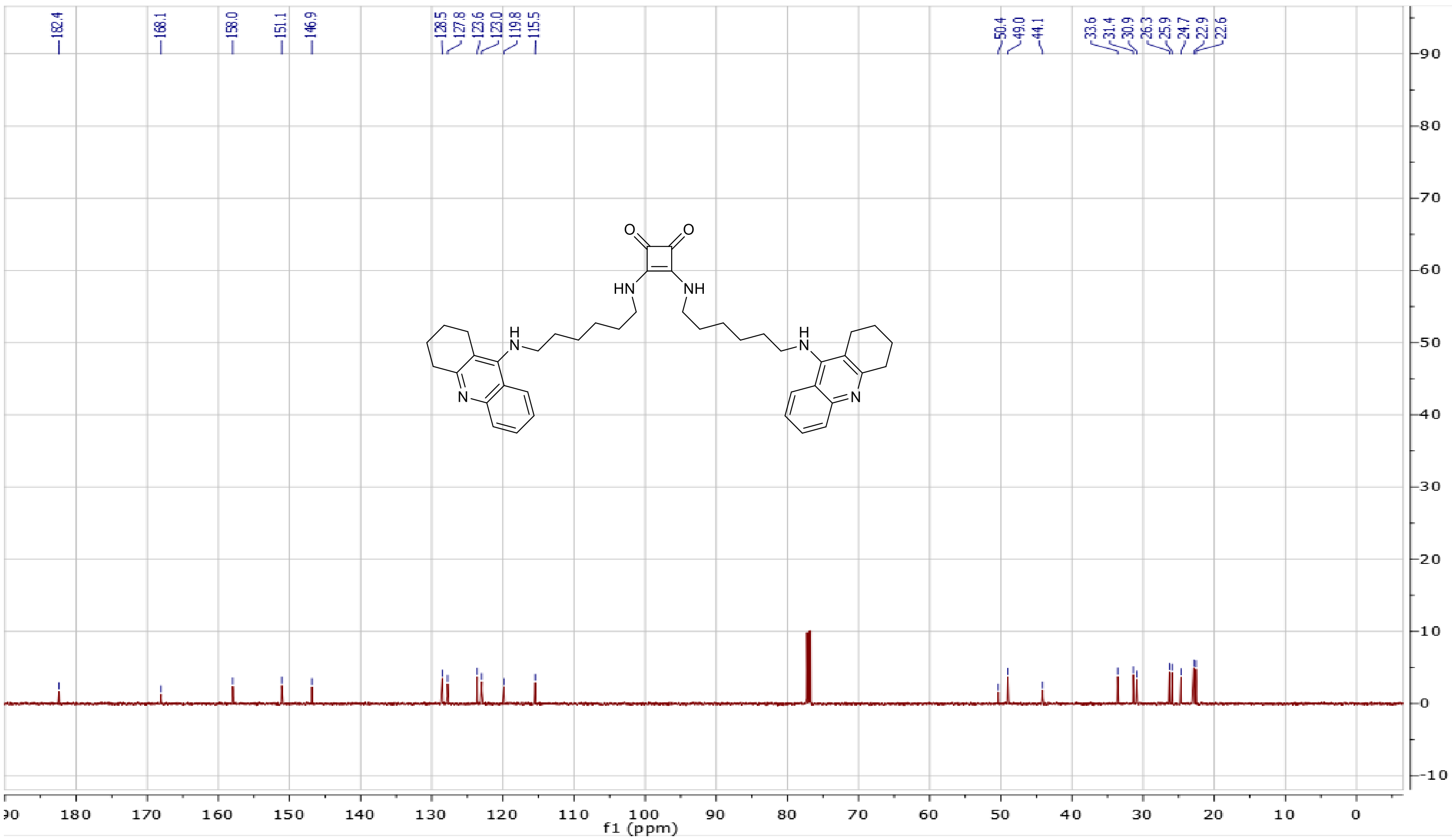

Bis({7-[(1,2,3,4-tetrahydroacridin-9-yl)amino]heptyl}amino)cyclobut-3-ene-1,2-dione (3f) ^{1}H NMR:

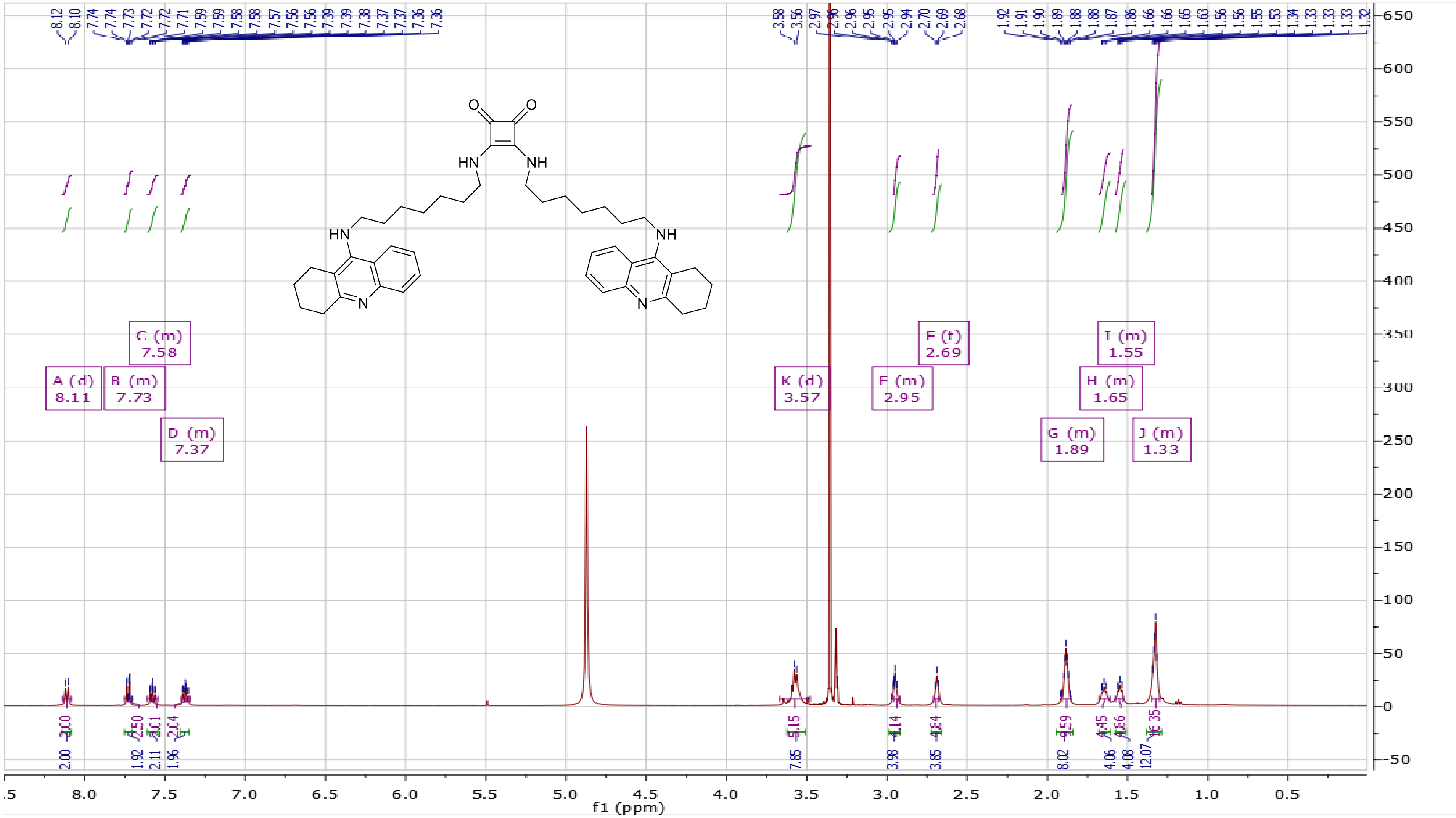

Bis({7-[(1,2,3,4-tetrahydroacridin-9-yl)amino]heptyl}amino)cyclobut-3-ene-1,2-dione (3f) ^{13}C NMR:

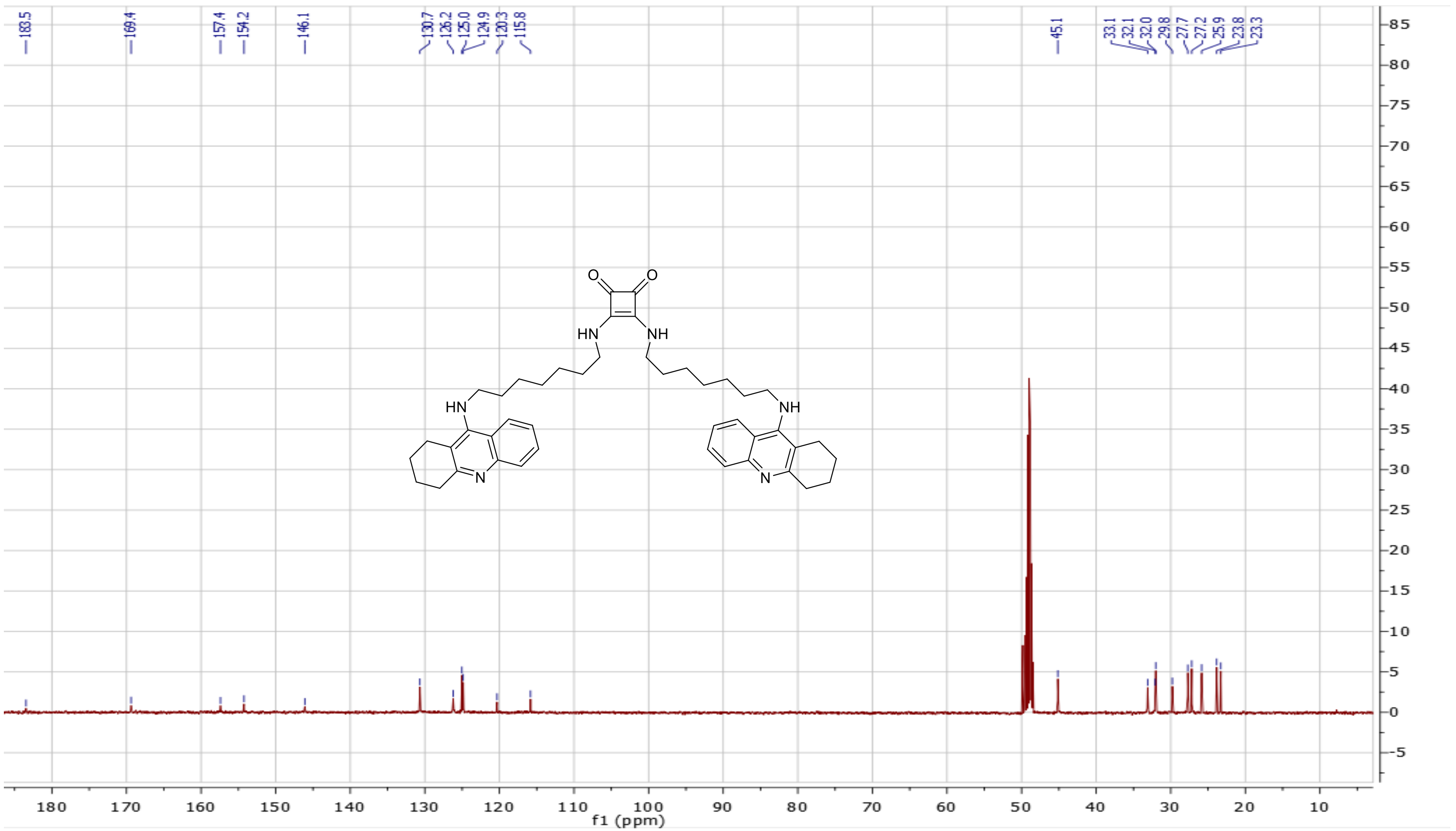

Bis({8-[(1,2,3,4-tetrahydroacridin-9-yl)amino]octyl}amino)cyclobut-3-ene-1,2-dione (3g) ^{1}H NMR:

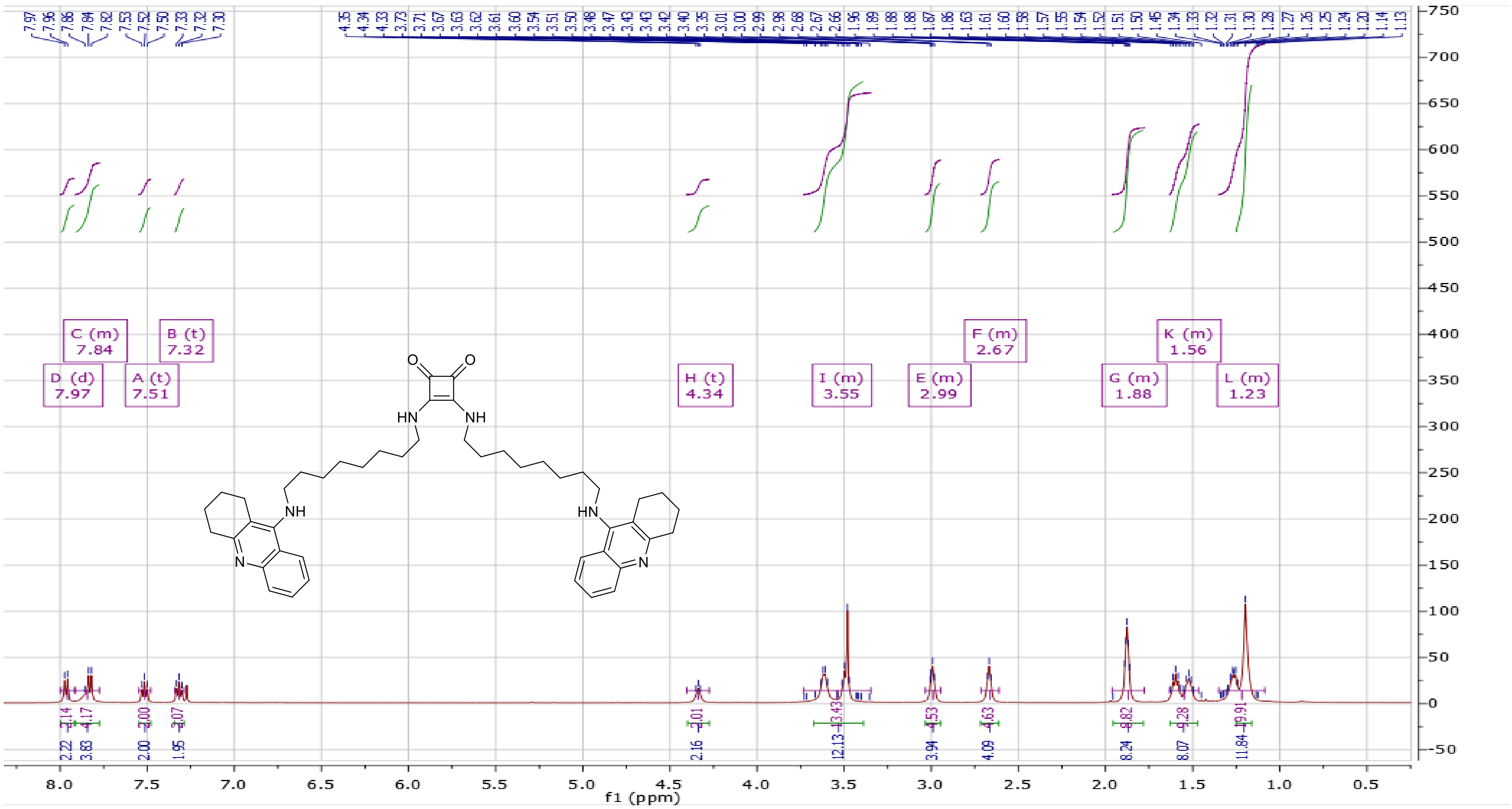

Bis({8-[(1,2,3,4-tetrahydroacridin-9-yl)amino]octyl}amino)cyclobut-3-ene-1,2-dione (3g) ^{13}C NMR:

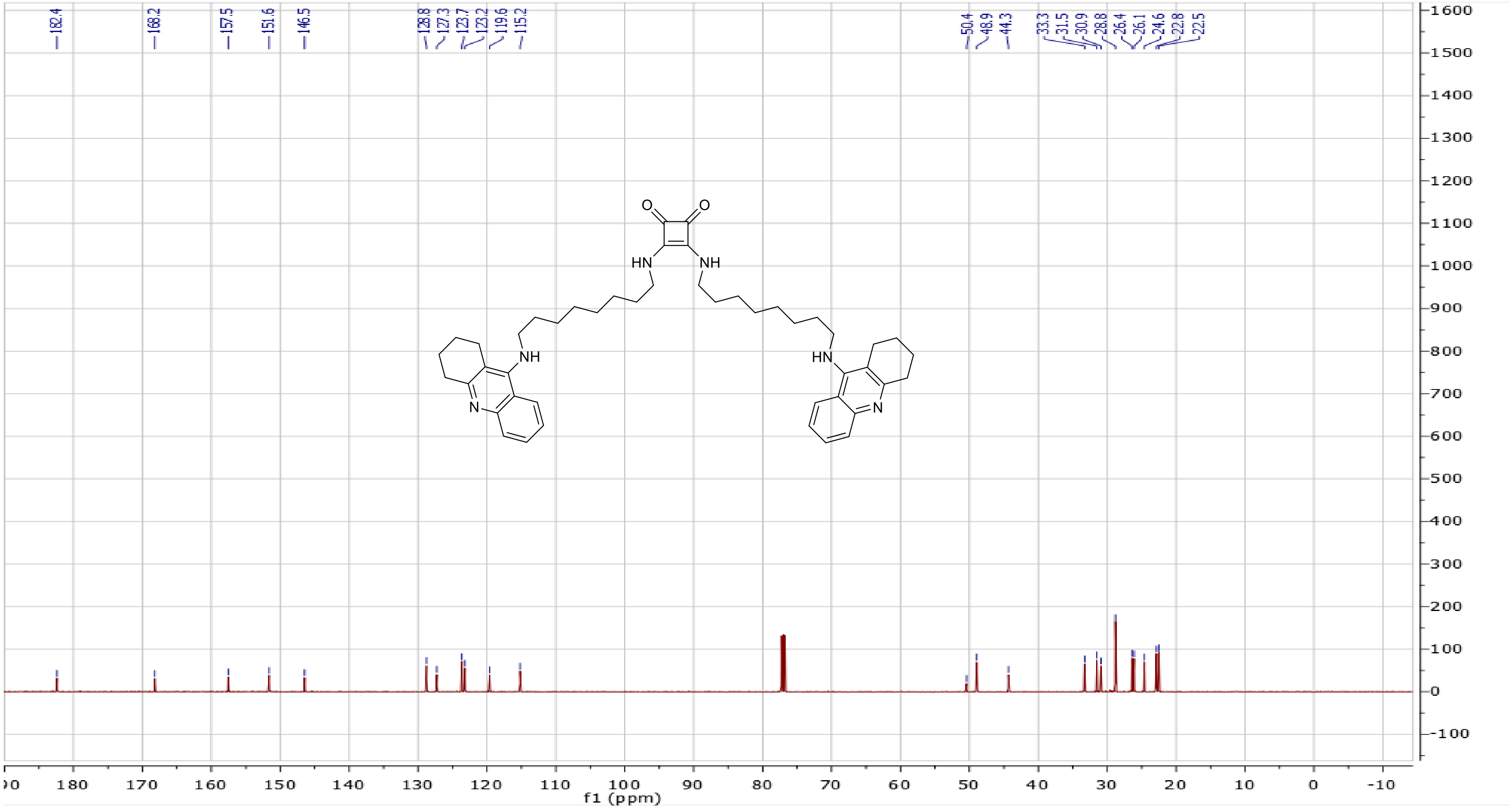

Bis({2-[(1,2,3,4-tetrahydroacridin-9-yl)amino]ethyl}amino)cyclobut-3-ene-1,2-dione (4a) ^{1}H NMR:

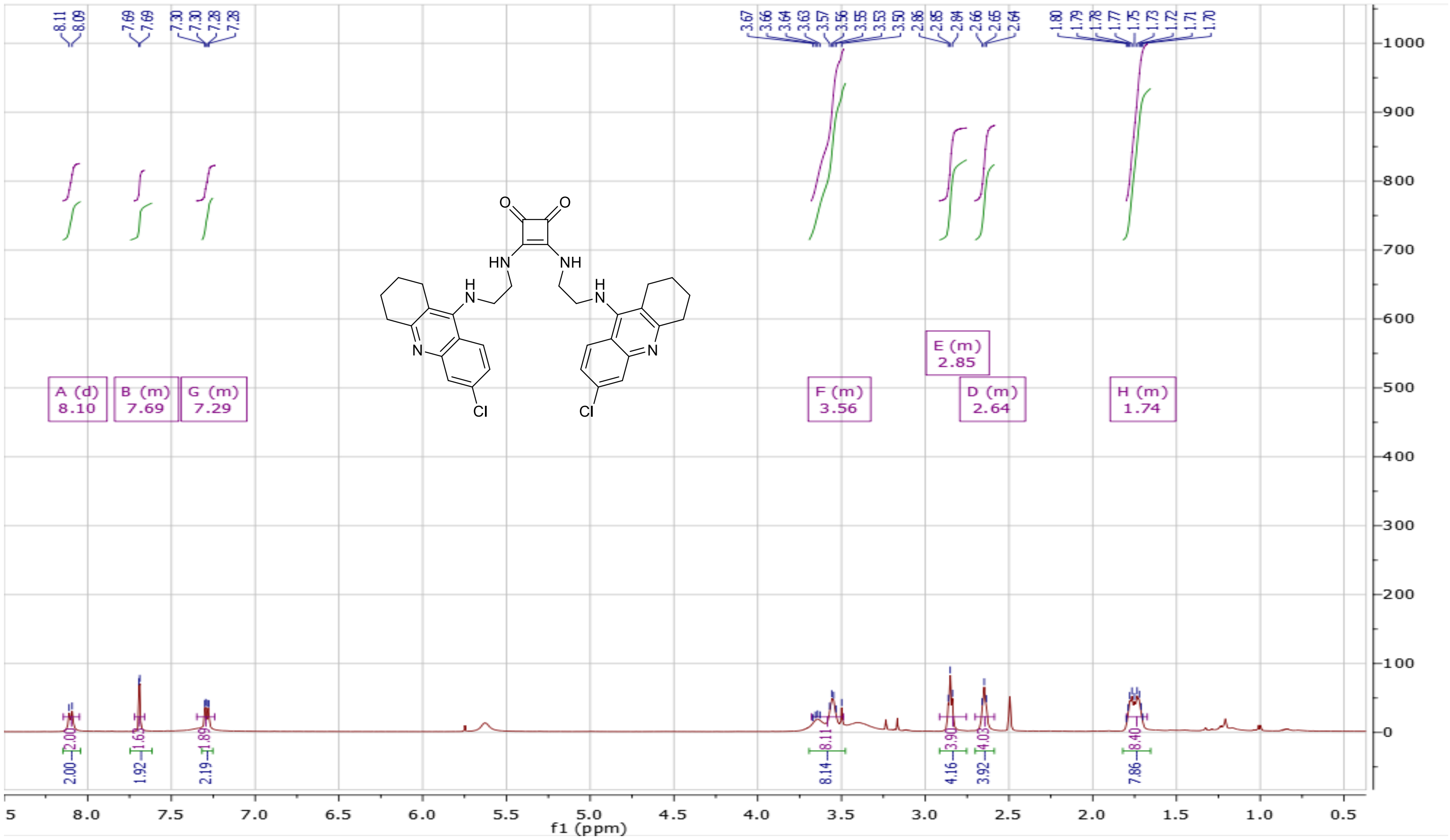

Bis({2-[(1,2,3,4-tetrahydroacridin-9-yl)amino]ethyl}amino)cyclobut-3-ene-1,2-dione (4a) ^{13}C NMR:

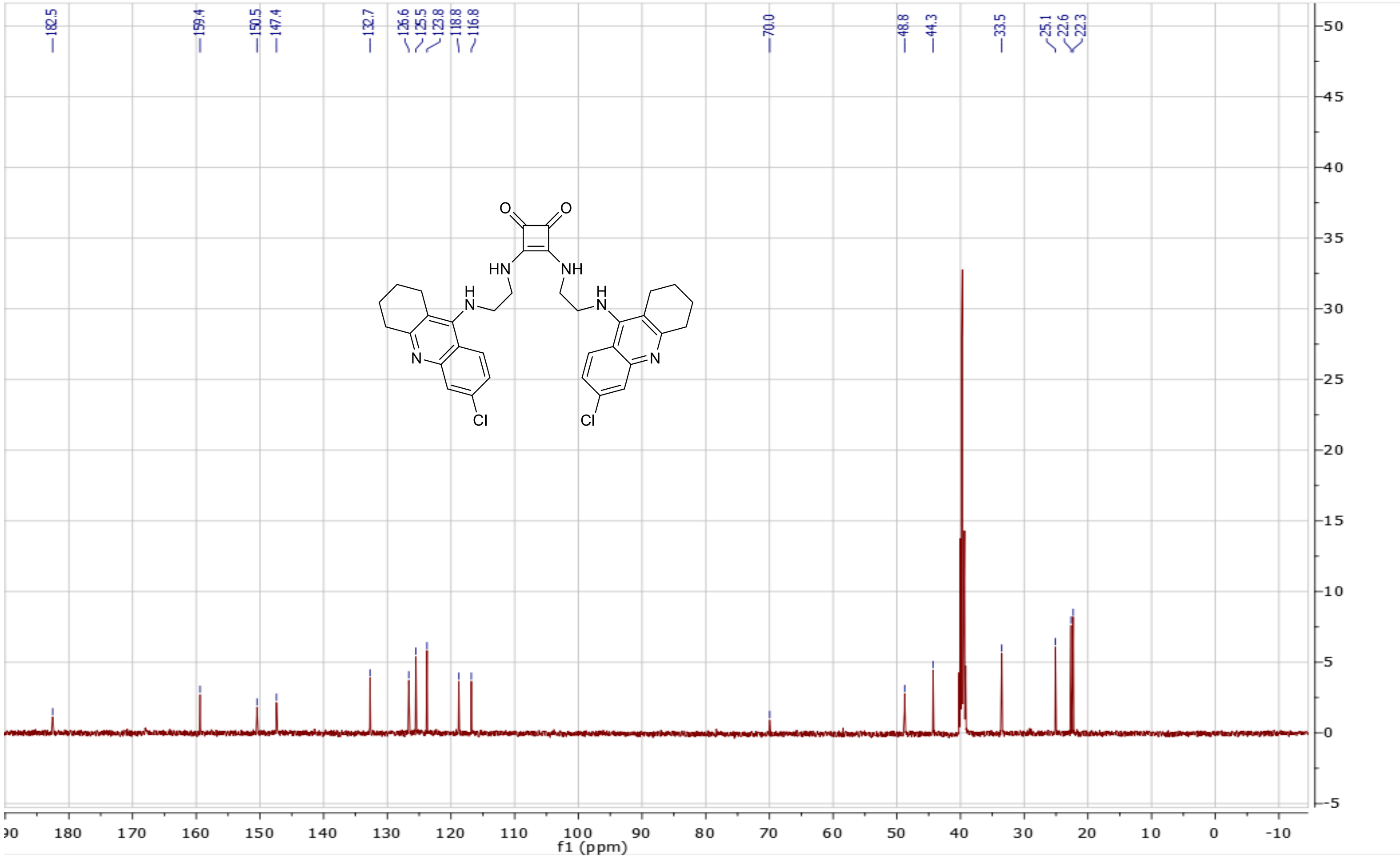

Bis({3-[(6-chloro-1,2,3,4-tetrahydroacridin-9-yl)amino]propyl}amino)cyclobut-3-ene-1,2-dione (4b) ^{1}H NMR:

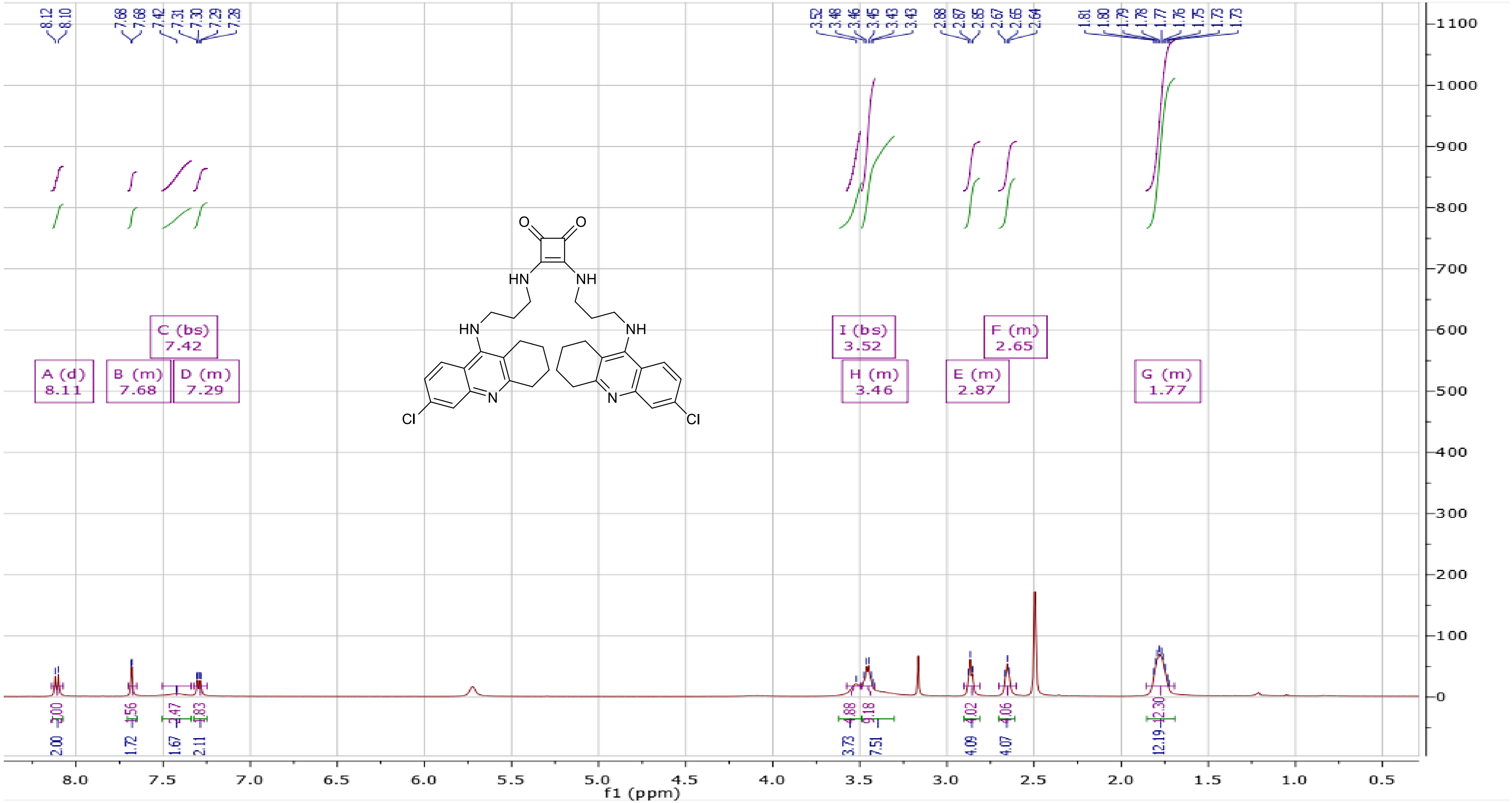

Bis({3-[(6-chloro-1,2,3,4-tetrahydroacridin-9-yl)amino]propyl}amino)cyclobut-3-ene-1,2-dione (4b) ^{13}C NMR:

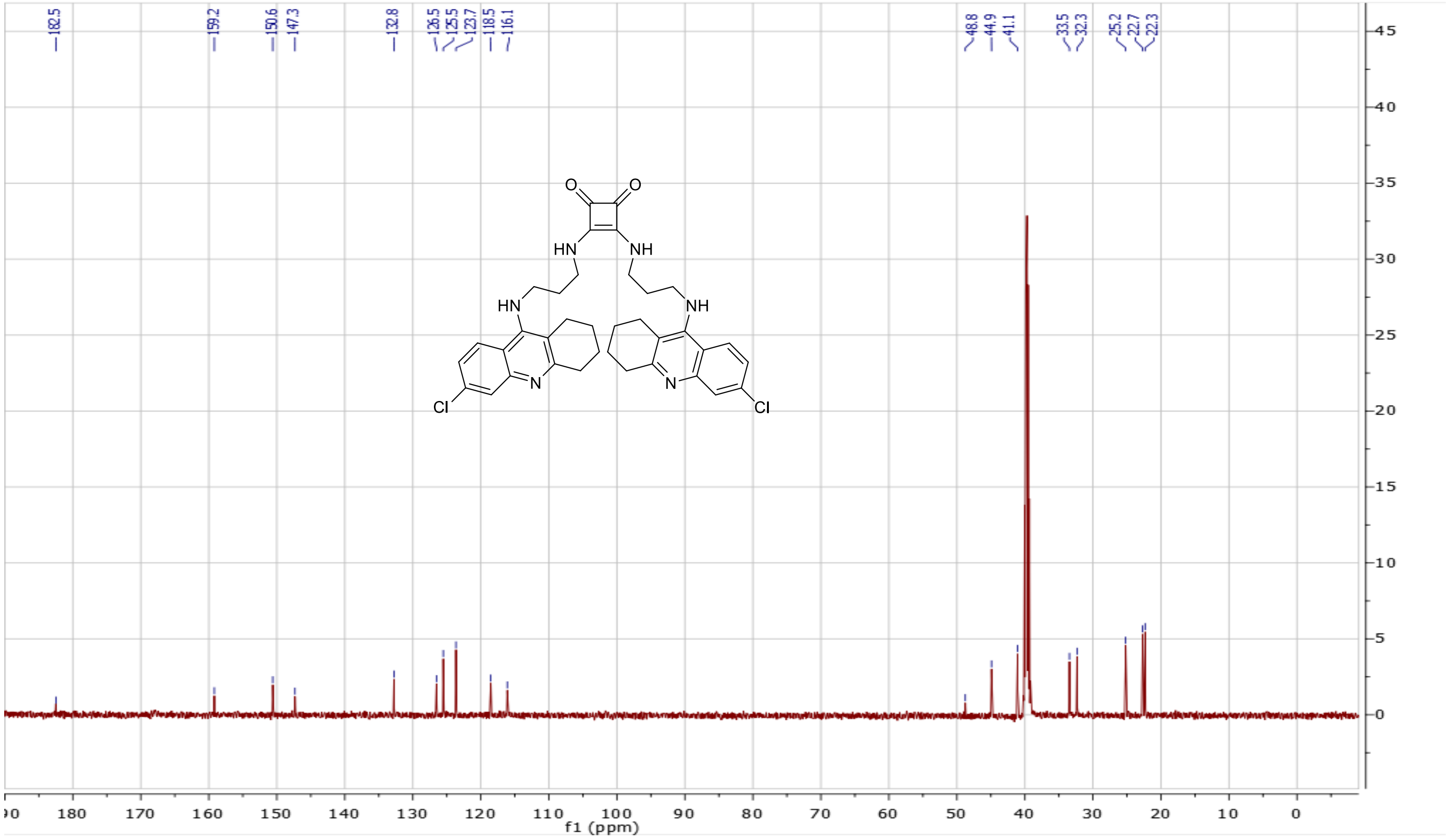

Bis({4-[(6-chloro-1,2,3,4-tetrahydroacridin-9-yl)amino]butyl}amino)cyclobut-3-ene-1,2-dione (4c) ^{1}H NMR:

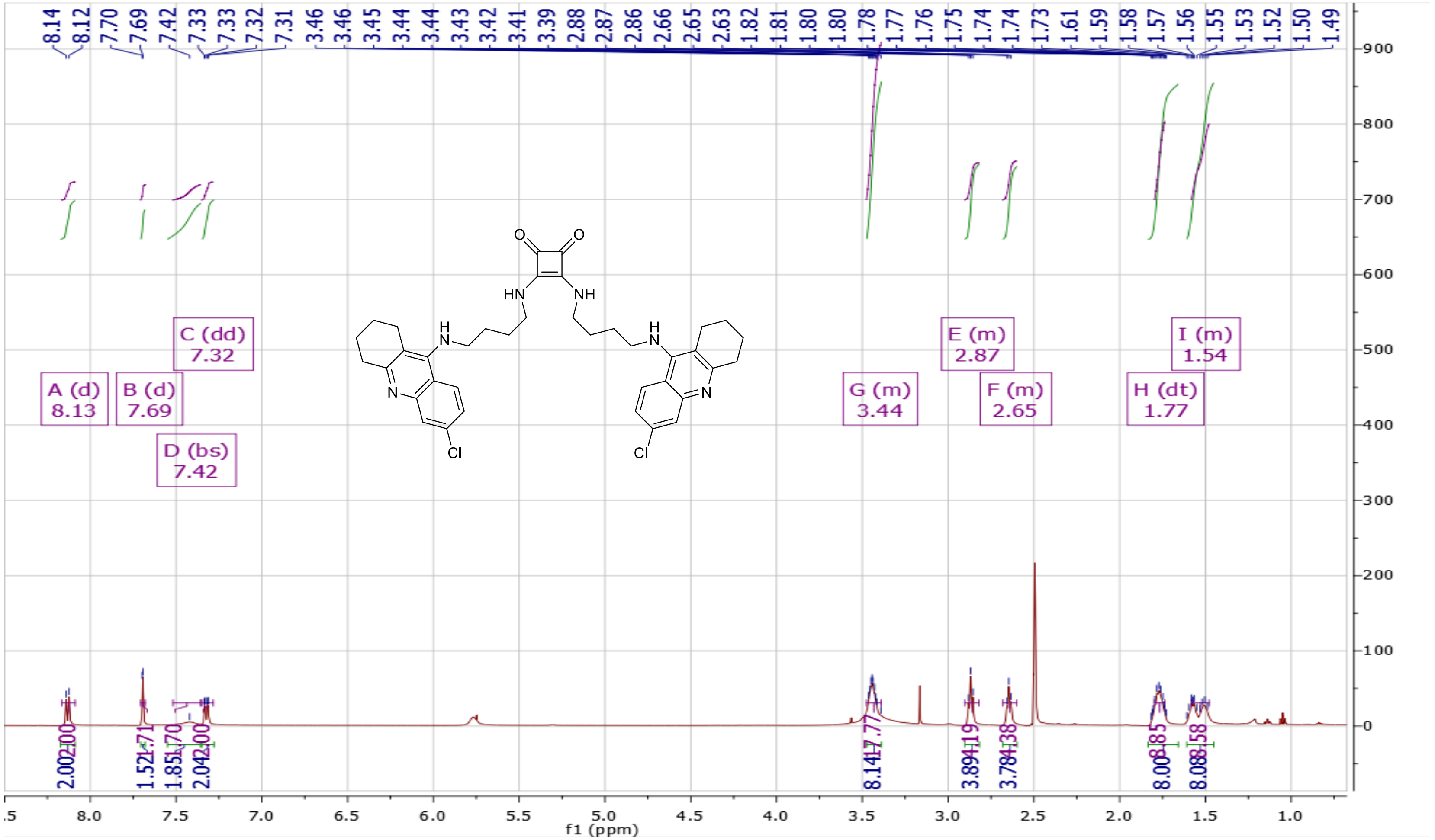

Bis({4-[(6-chloro-1,2,3,4-tetrahydroacridin-9-yl)amino]butyl}amino)cyclobut-3-ene-1,2-dione (4c) ^{13}C NMR:

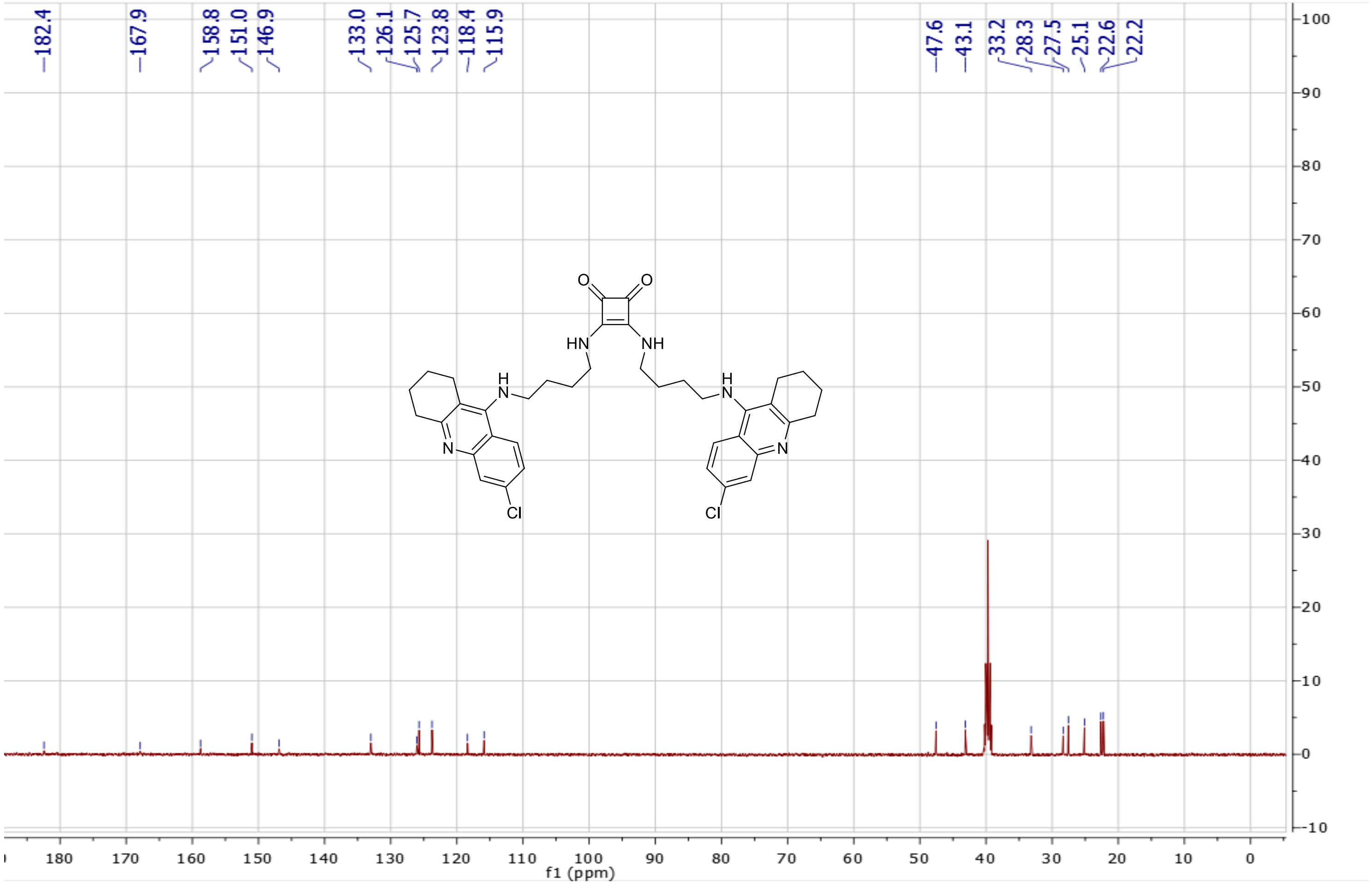

Bis({5-[(6-chloro-1,2,3,4-tetrahydroacridin-9-yl)amino]pentyl}amino)cyclobut-3-ene-1,2-dione (4d) ^{1}H NMR:

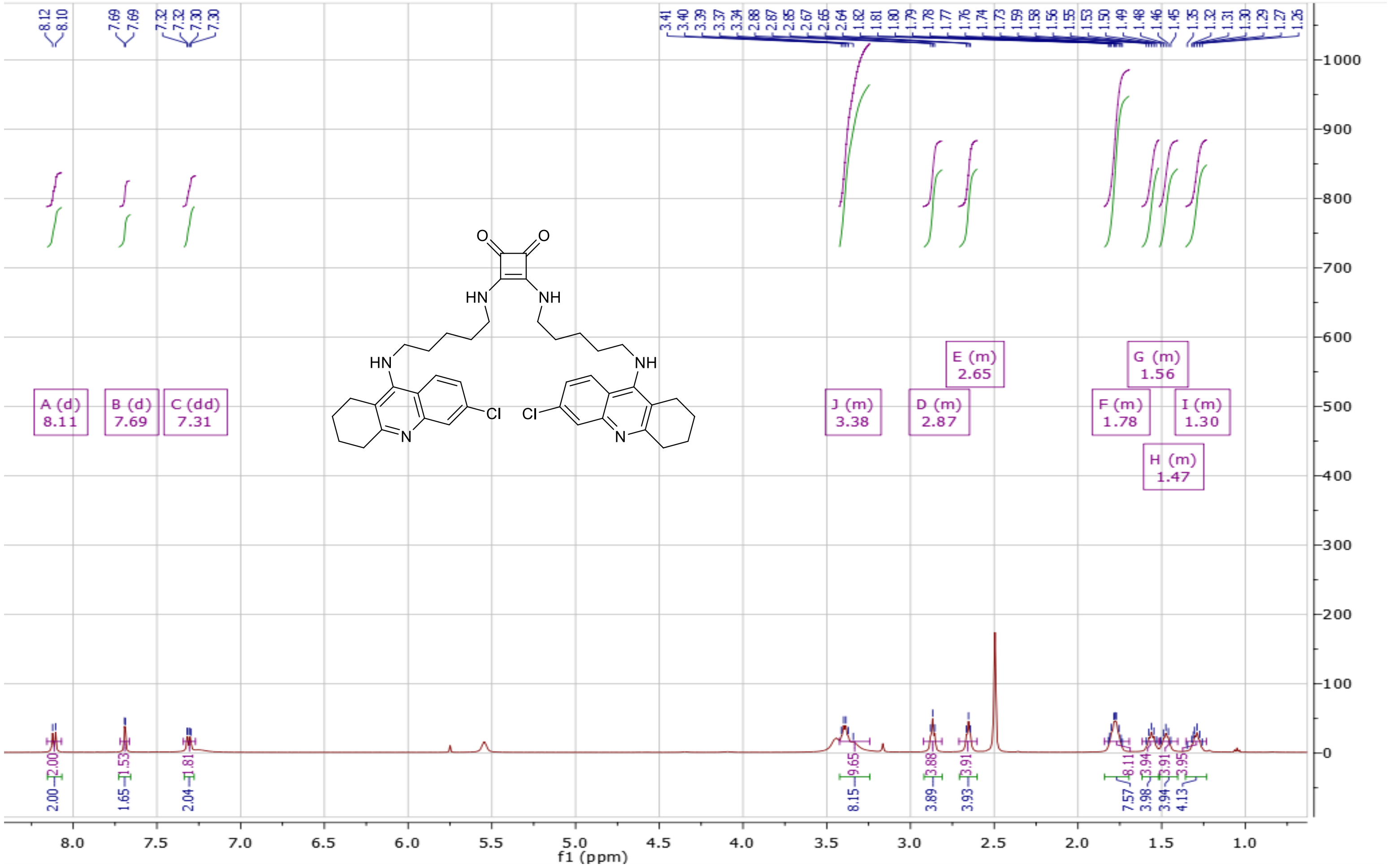

Bis({5-[(6-chloro-1,2,3,4-tetrahydroacridin-9-yl)amino]pentyl}amino)cyclobut-3-ene-1,2-dione (4d) ^{13}C NMR:

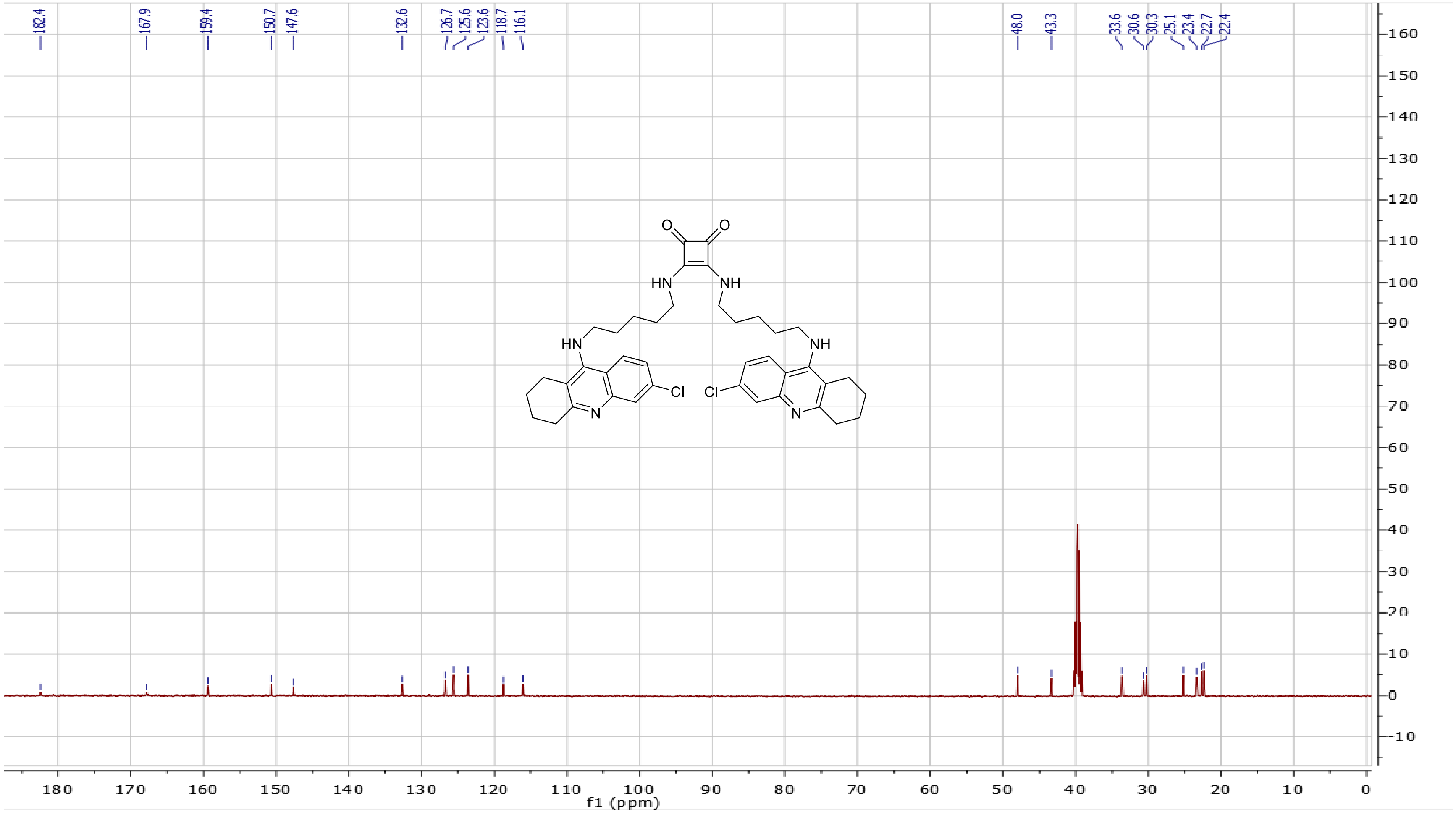

Bis({6-[(6-chloro-1,2,3,4-tetrahydroacridin-9-yl)amino]hexyl}amino)cyclobut-3-ene-1,2-dione (4e) ^{1}H NMR:

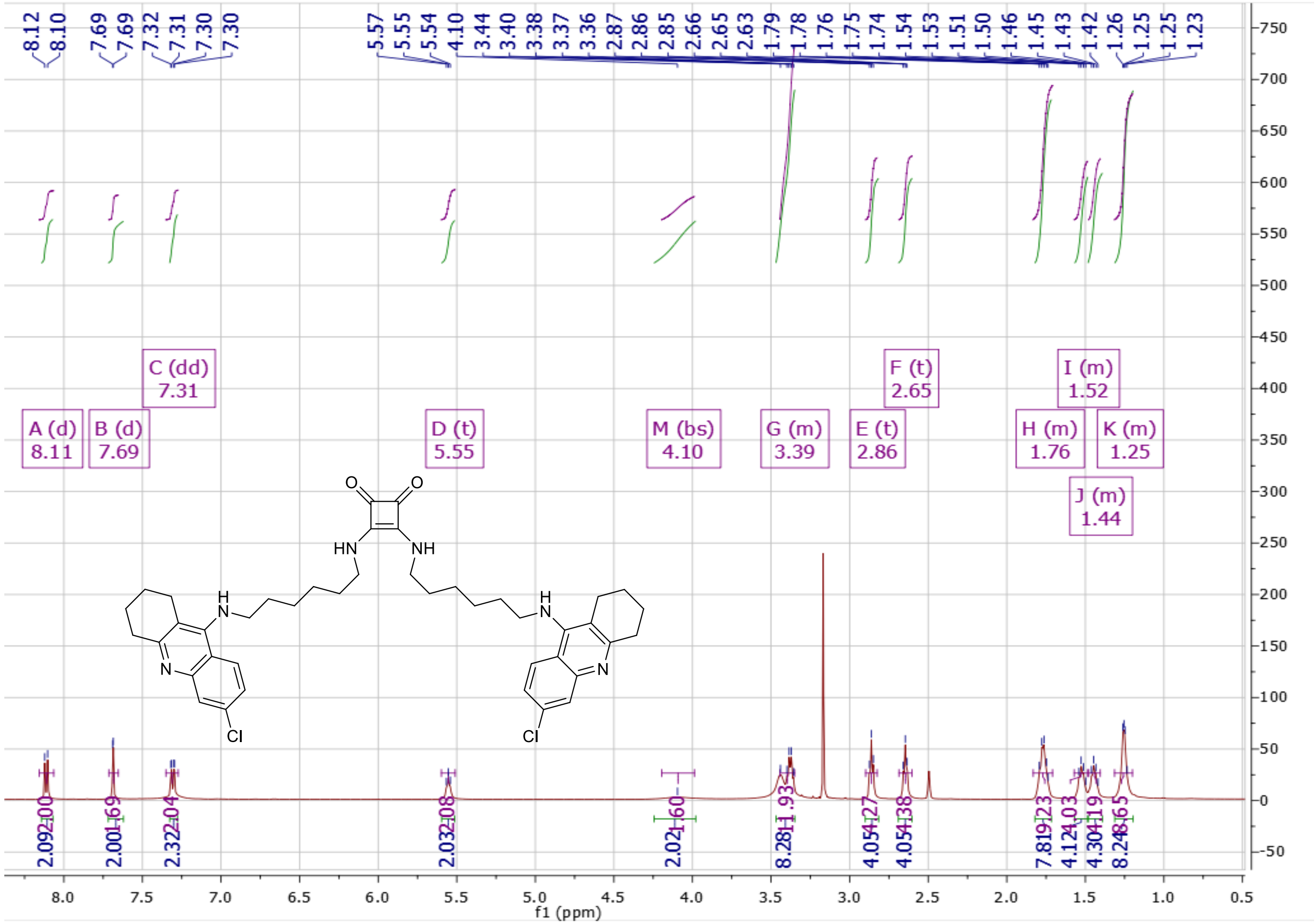

Bis({6-[(6-chloro-1,2,3,4-tetrahydroacridin-9-yl)amino]hexyl}amino)cyclobut-3-ene-1,2-dione (4e) ^{13}C NMR:

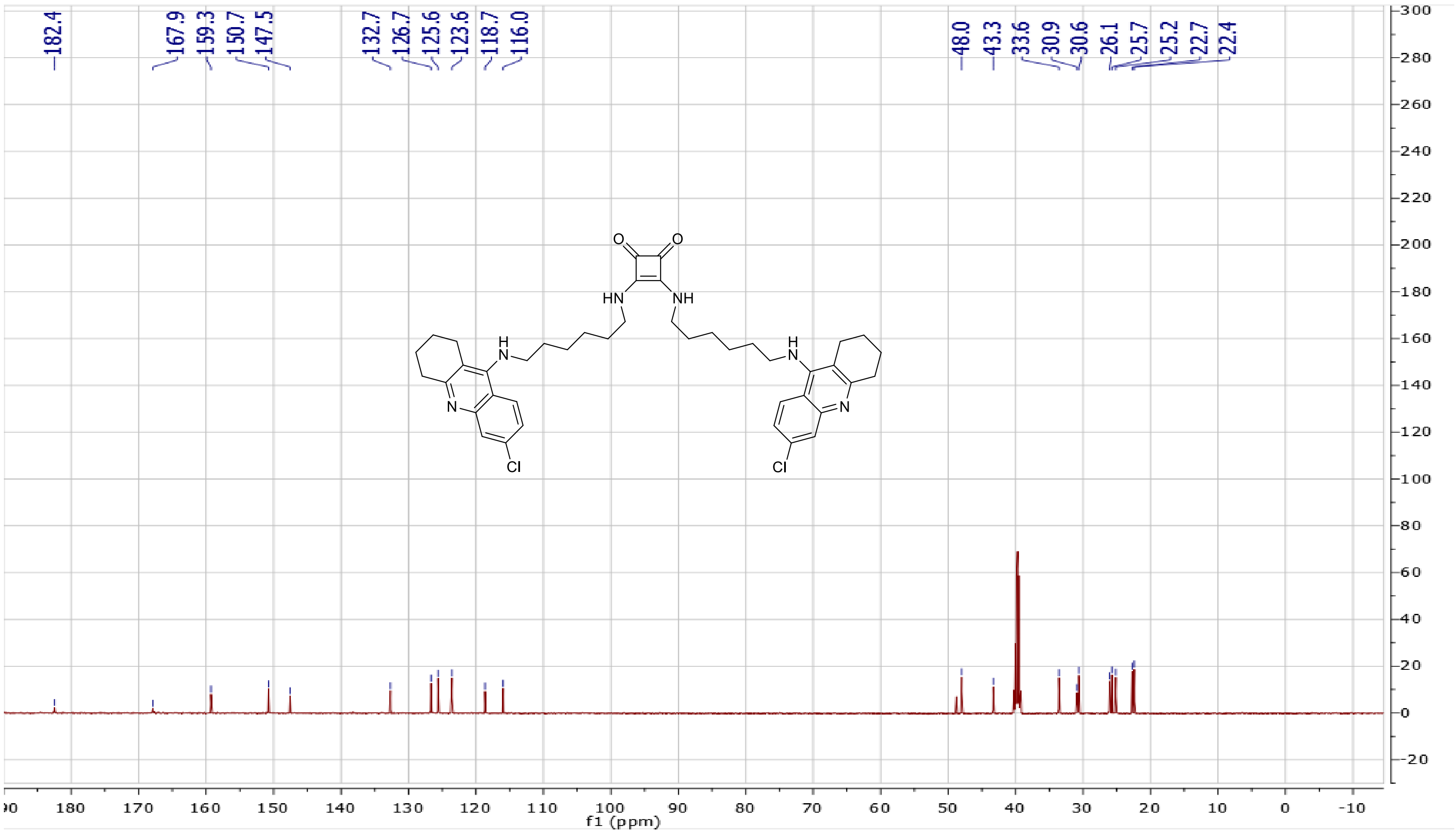

Bis({7-[(6-chloro-1,2,3,4-tetrahydroacridin-9-yl)amino]heptyl}amino)cyclobut-3-ene-1,2-dione (4f) ^{1}H NMR:

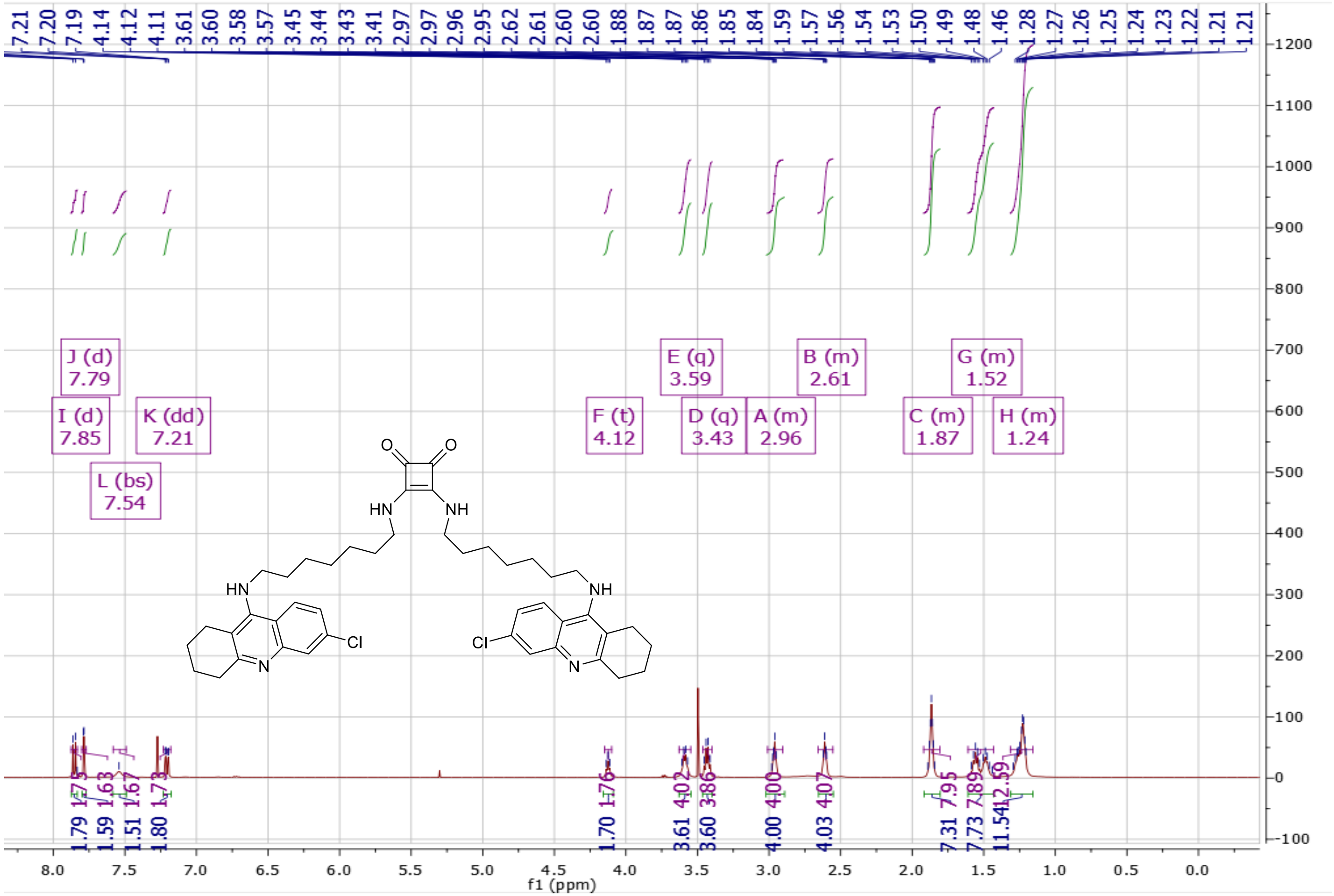

Bis({7-[(6-chloro-1,2,3,4-tetrahydroacridin-9-yl)amino]heptyl}amino)cyclobut-3-ene-1,2-dione (4f) ^{13}C NMR:

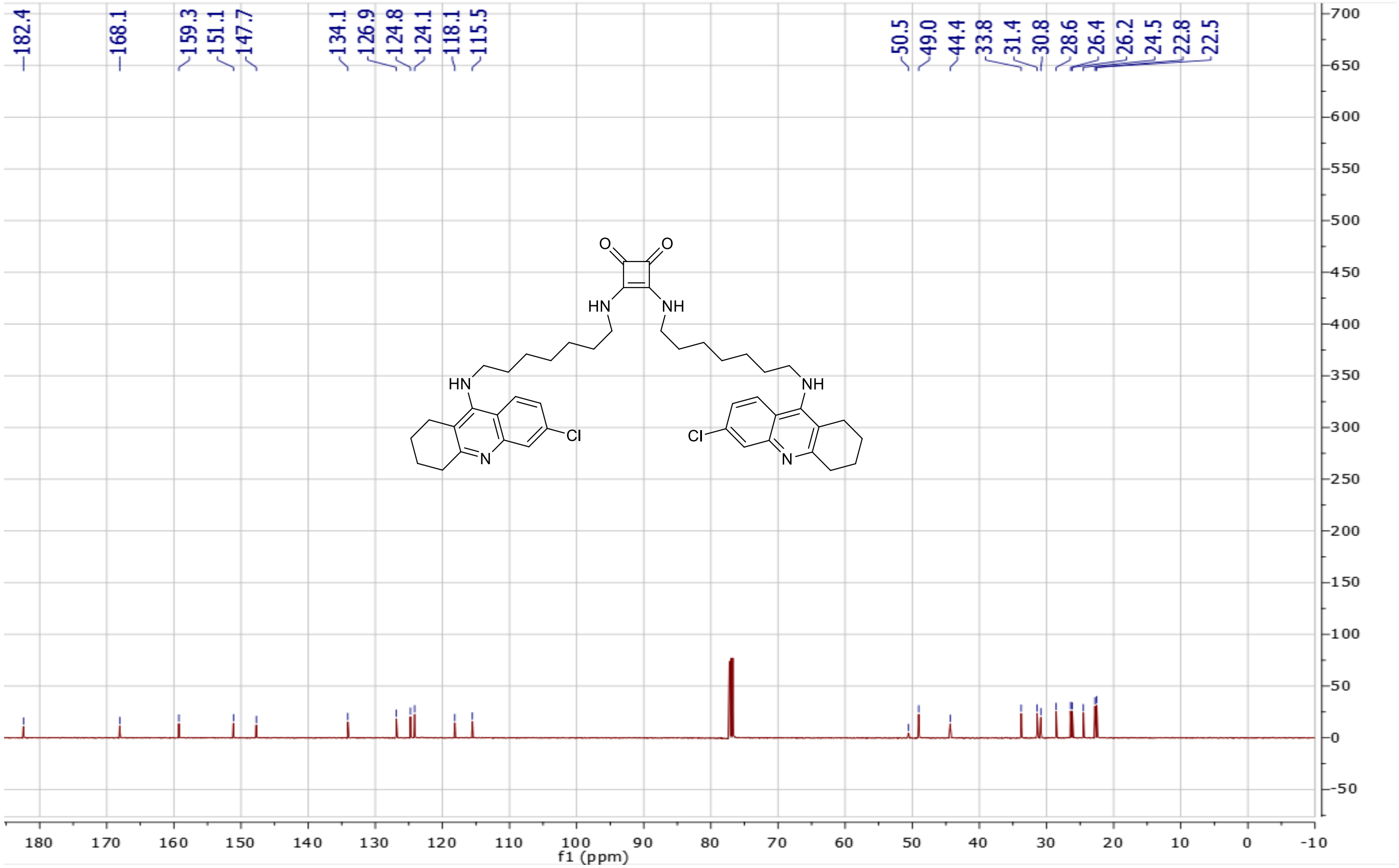

Bis({8-[(6-chloro-1,2,3,4-tetrahydroacridin-9-yl)amino]octyl}amino)cyclobut-3-ene-1,2-dione (4g) ^{1}H NMR:

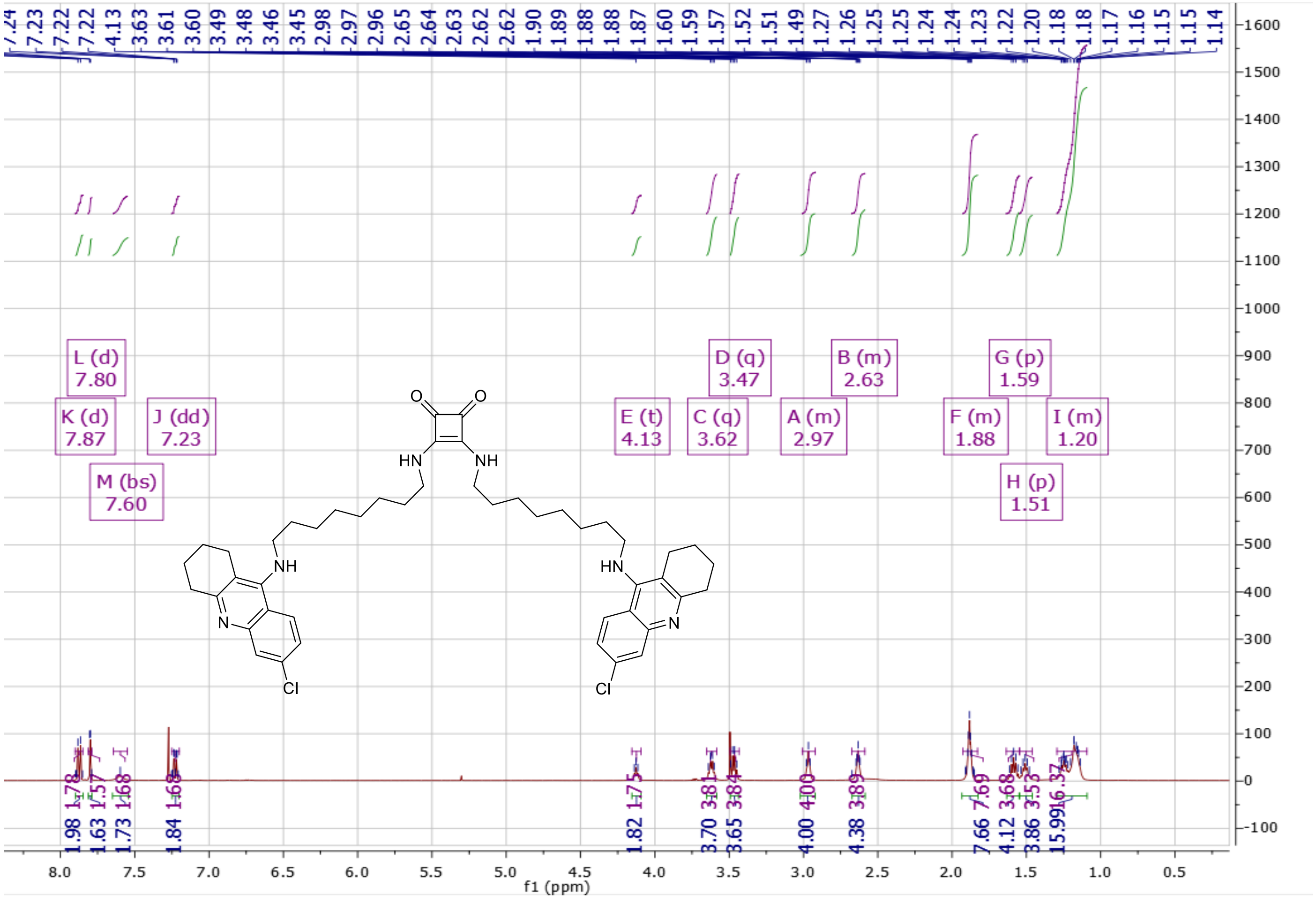

Bis({8-[(6-chloro-1,2,3,4-tetrahydroacridin-9-yl)amino]octyl}amino)cyclobut-3-ene-1,2-dione (4g) ^{13}C NMR:

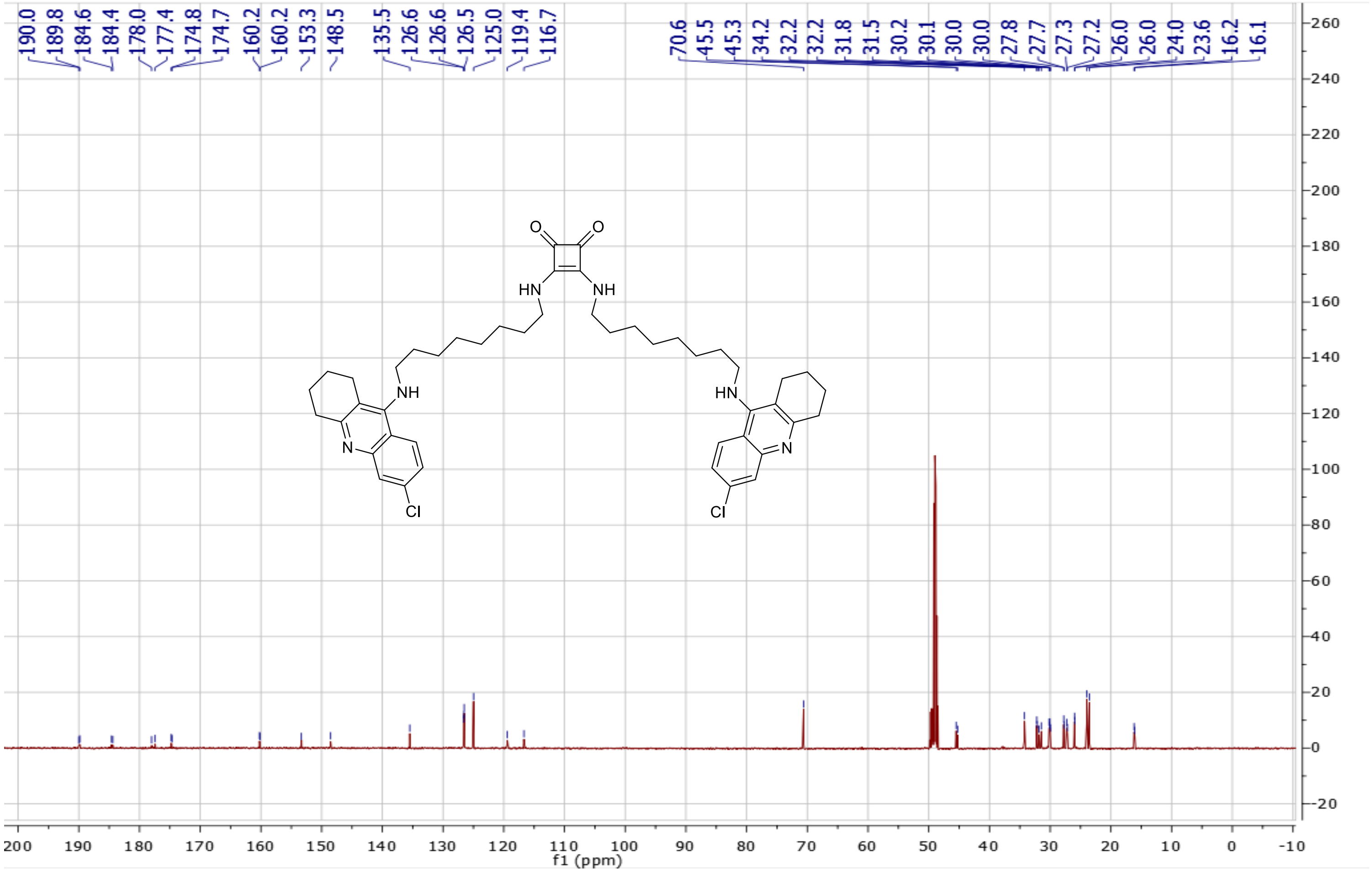

Bis({2-[(7-methoxy-1,2,3,4-tetrahydroacridin-9-yl)amino]ethyl}amino)cyclobut-3-ene-1,2-dione (5a) ^{1}H NMR:

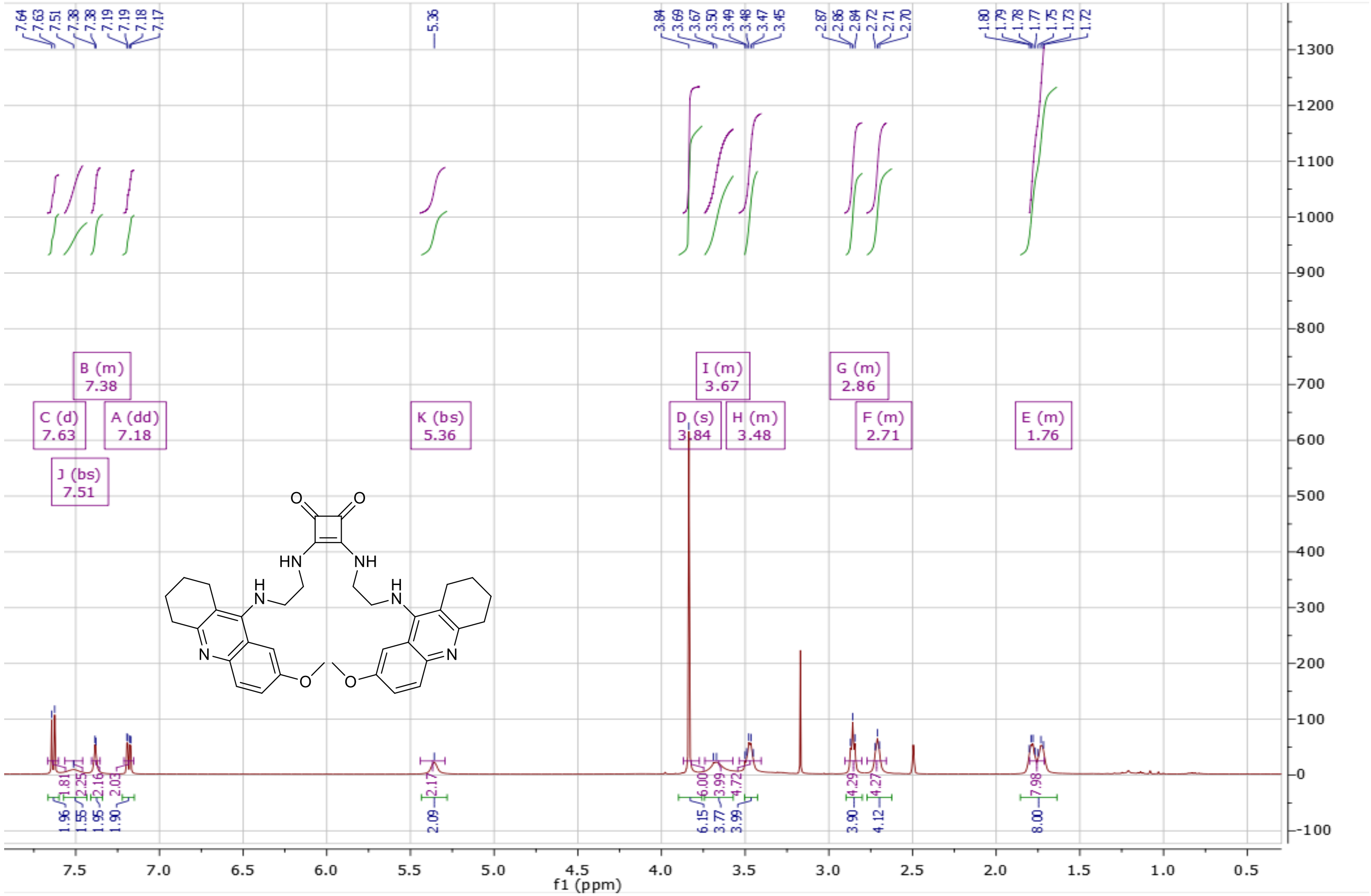

Bis({2-[(7-methoxy-1,2,3,4-tetrahydroacridin-9-yl)amino]ethyl}amino)cyclobut-3-ene-1,2-dione (5a) ^{13}C NMR:

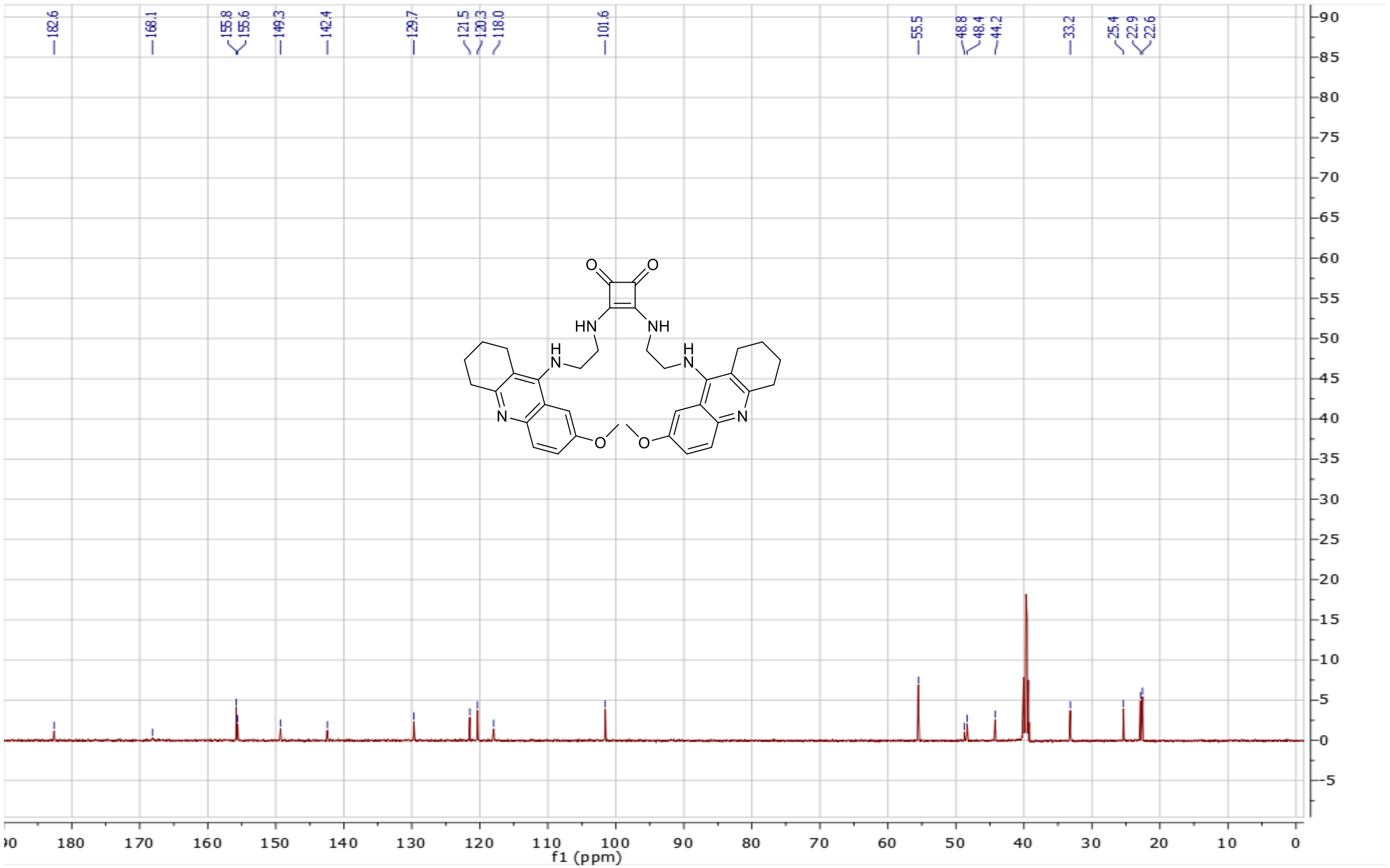

Bis({3-[(7-methoxy-1,2,3,4-tetrahydroacridin-9-yl)amino]propyl}amino)cyclobut-3-ene-1,2-dione (5b) ^{1}H NMR:

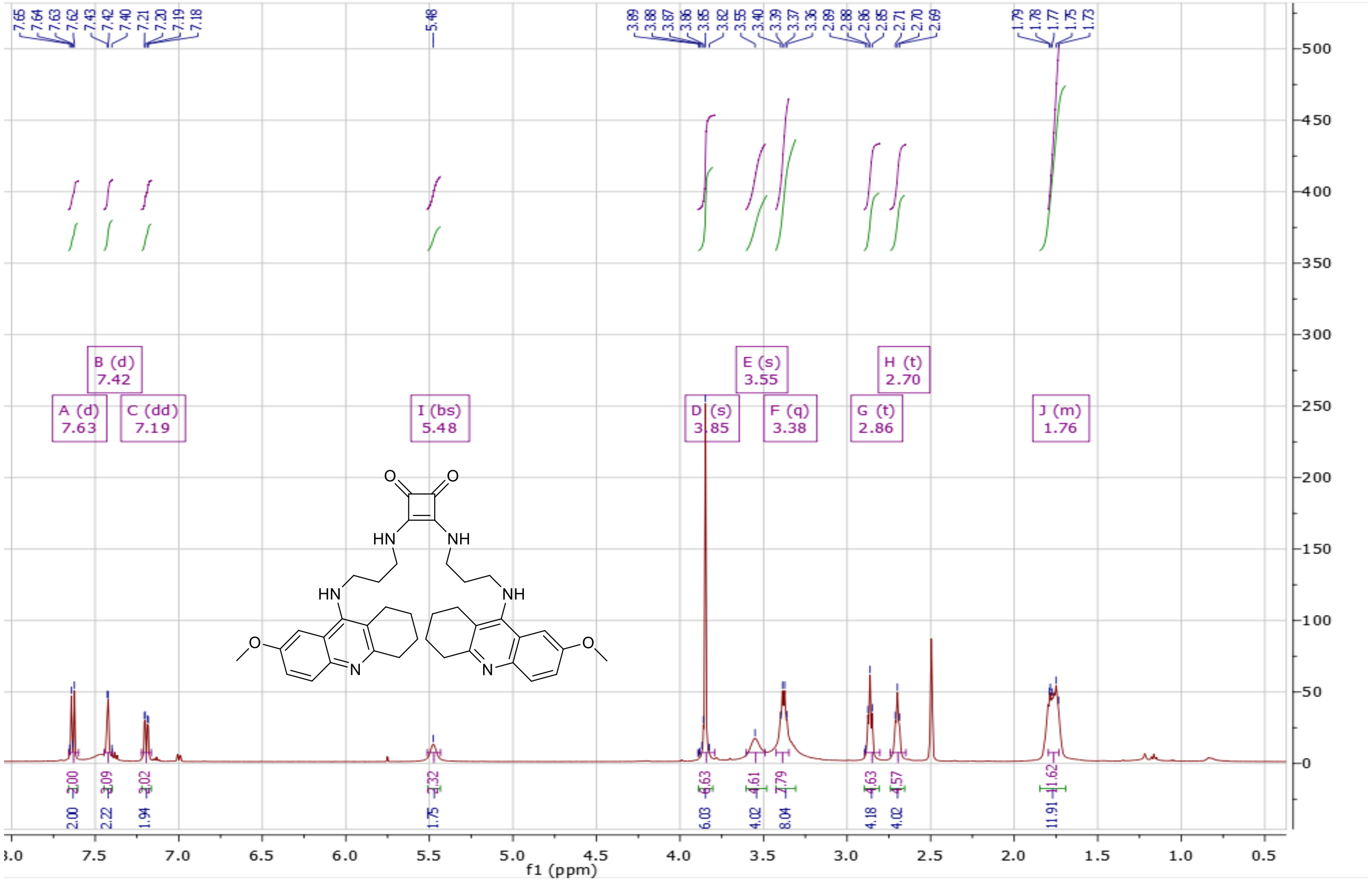

Bis({3-[(7-methoxy-1,2,3,4-tetrahydroacridin-9-yl)amino]propyl}amino)cyclobut-3-ene-1,2-dione (5b) ^{13}C NMR:

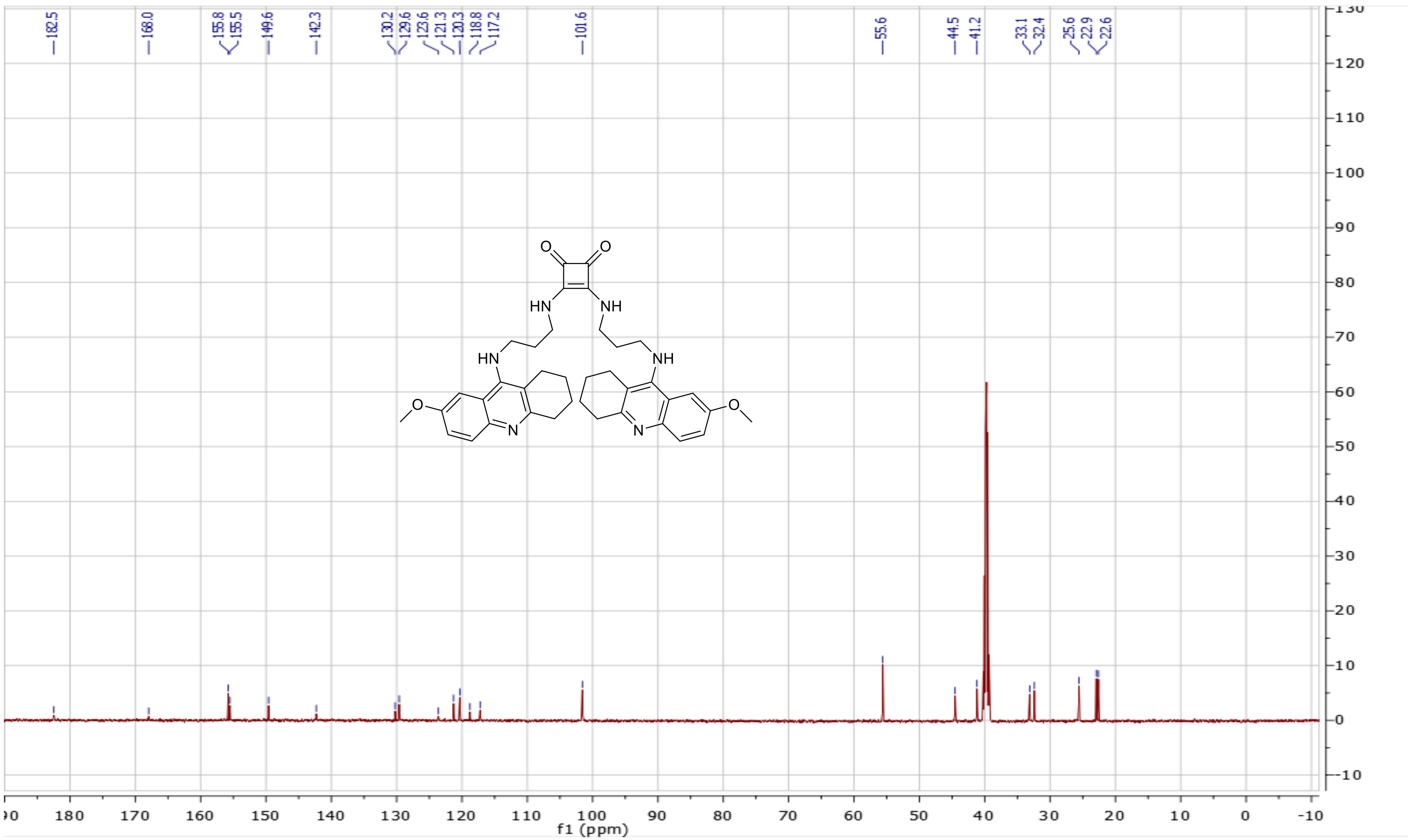

Bis({4-[(7-methoxy-1,2,3,4-tetrahydroacridin-9-yl)amino]butyl}amino)cyclobut-3-ene-1,2-dione (5c) ^{1}H NMR:

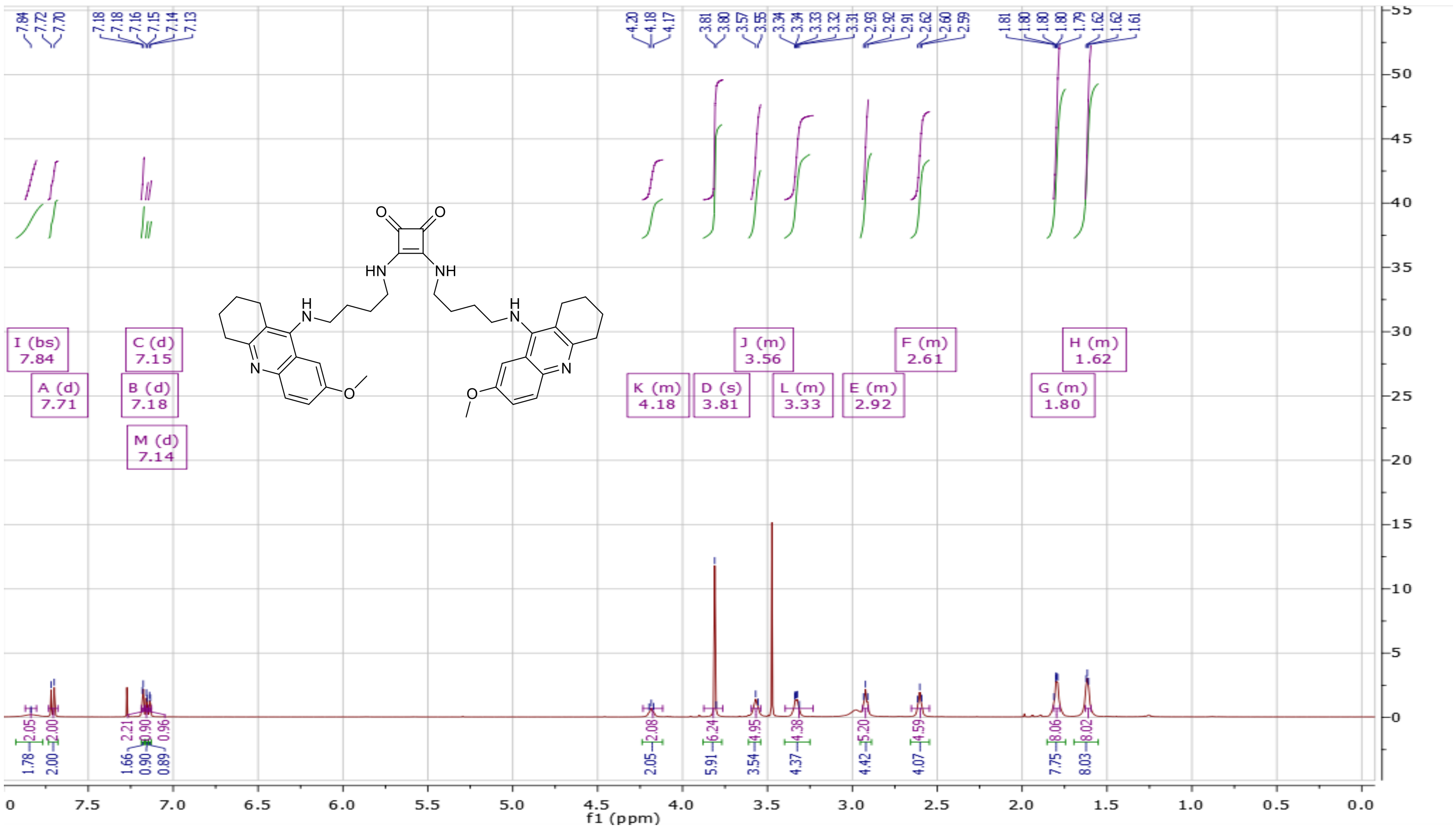

Bis({4-[(7-methoxy-1,2,3,4-tetrahydroacridin-9-yl)amino]butyl}amino)cyclobut-3-ene-1,2-dione (5c) ^{13}C NMR:

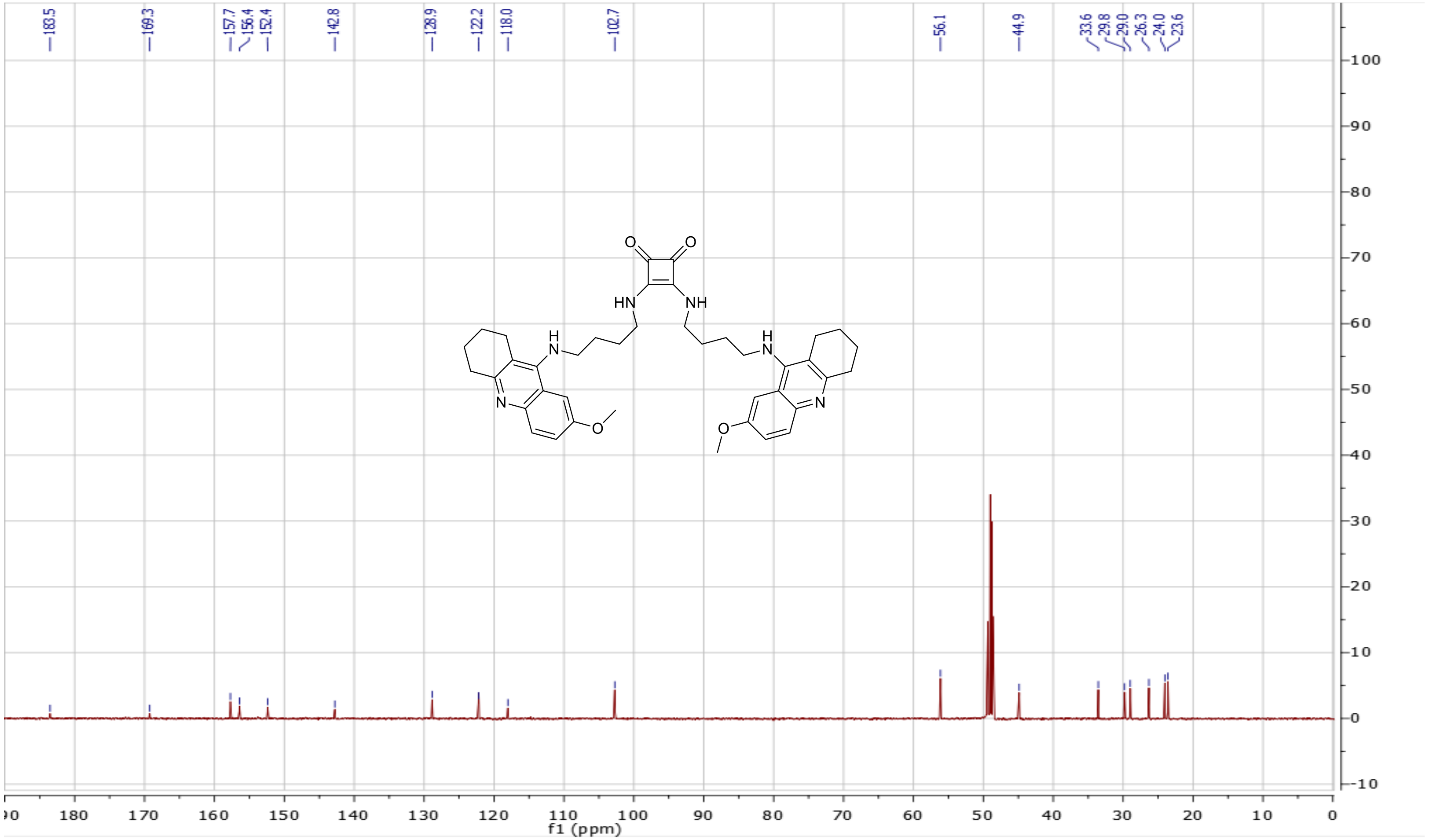

Bis({5-[(7-methoxy-1,2,3,4-tetrahydroacridin-9-yl)amino]pentyl}amino)cyclobut-3-ene-1,2-dione (5d) ^{1}H NMR:

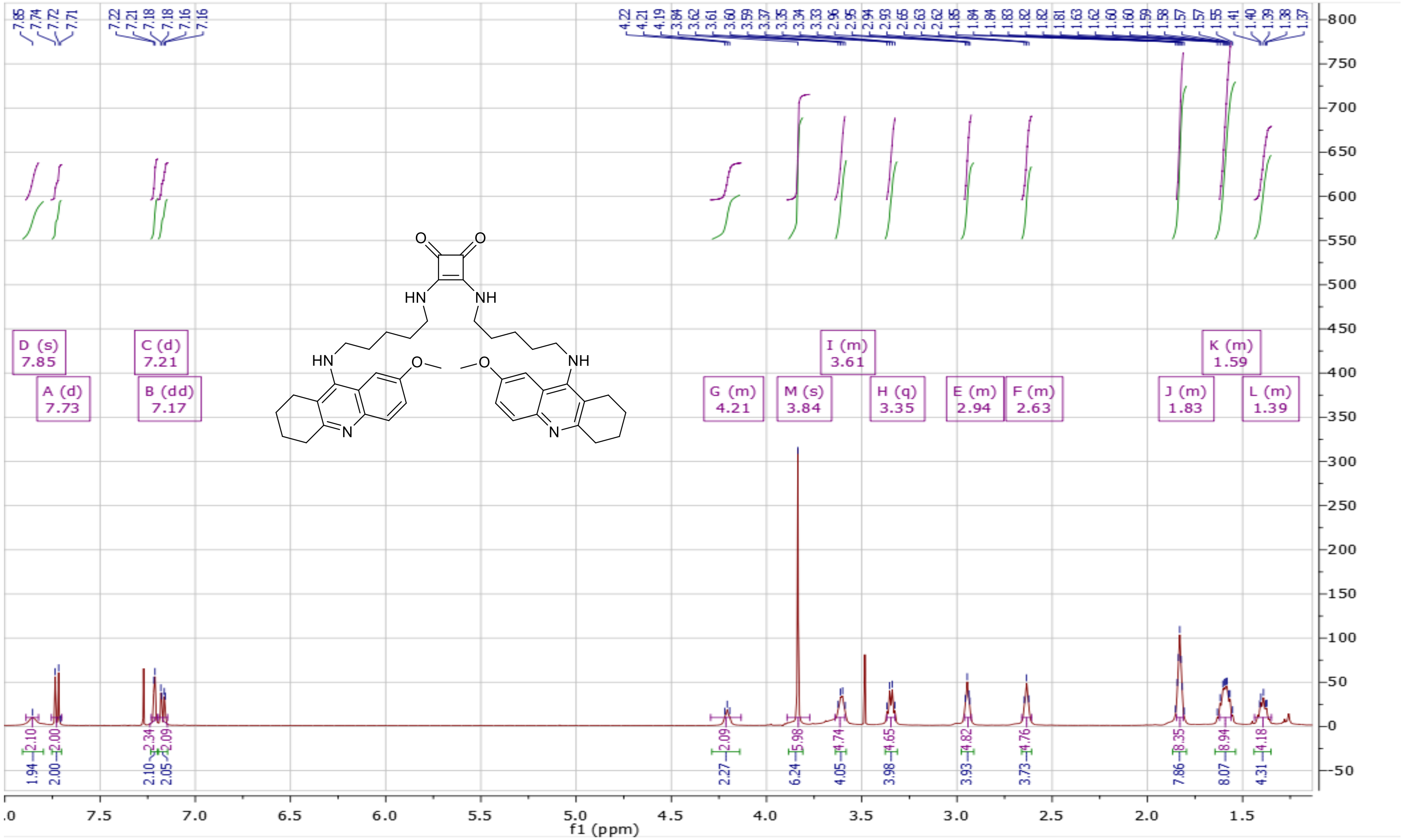

Bis({5-[(7-methoxy-1,2,3,4-tetrahydroacridin-9-yl)amino]pentyl}amino)cyclobut-3-ene-1,2-dione (5d) ^{13}C NMR:

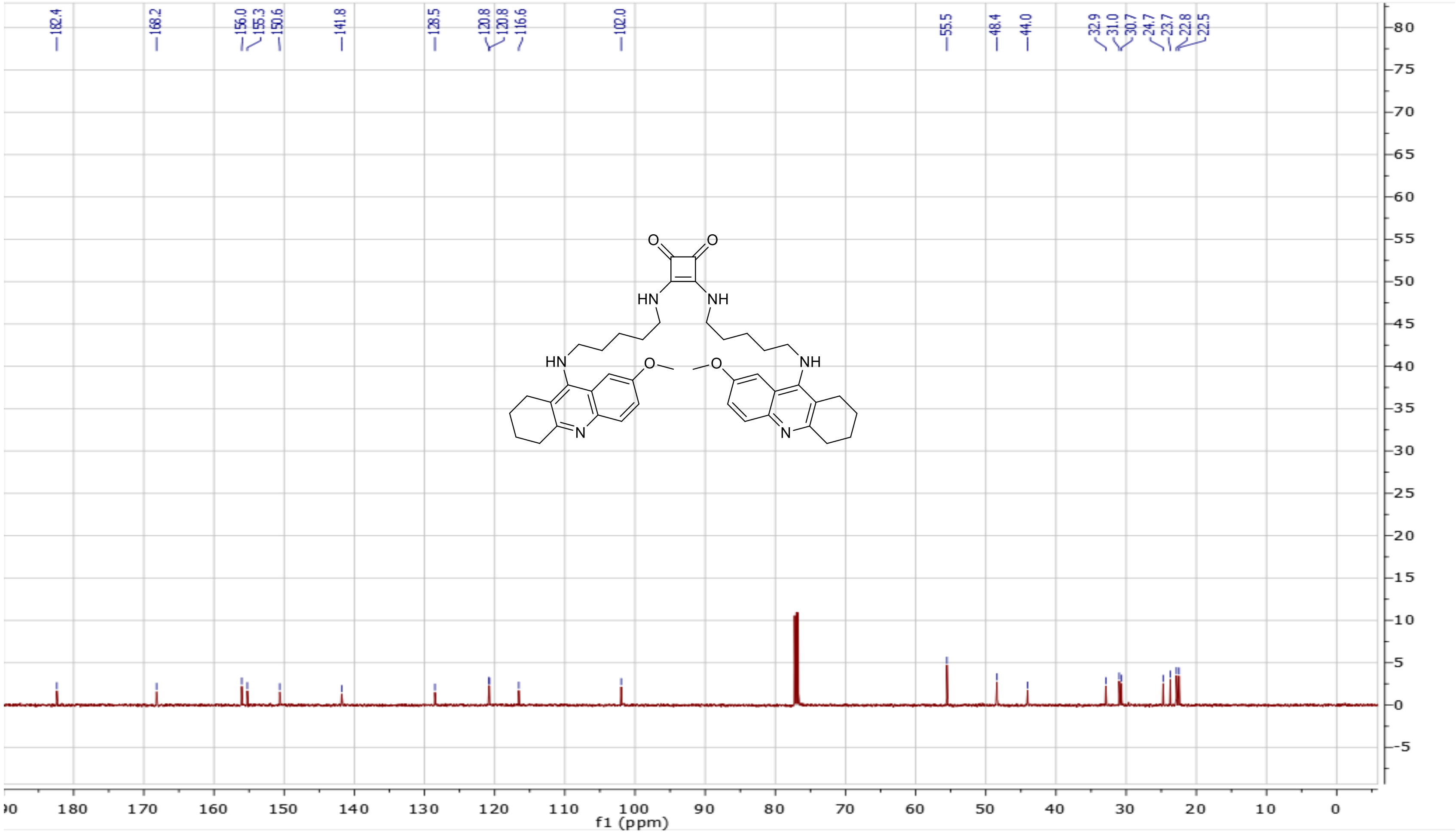

Bis({6-[(7-methoxy-1,2,3,4-tetrahydroacridin-9-yl)amino]hexyl}amino)cyclobut-3-ene-1,2-dione (5e) ^{1}H NMR:

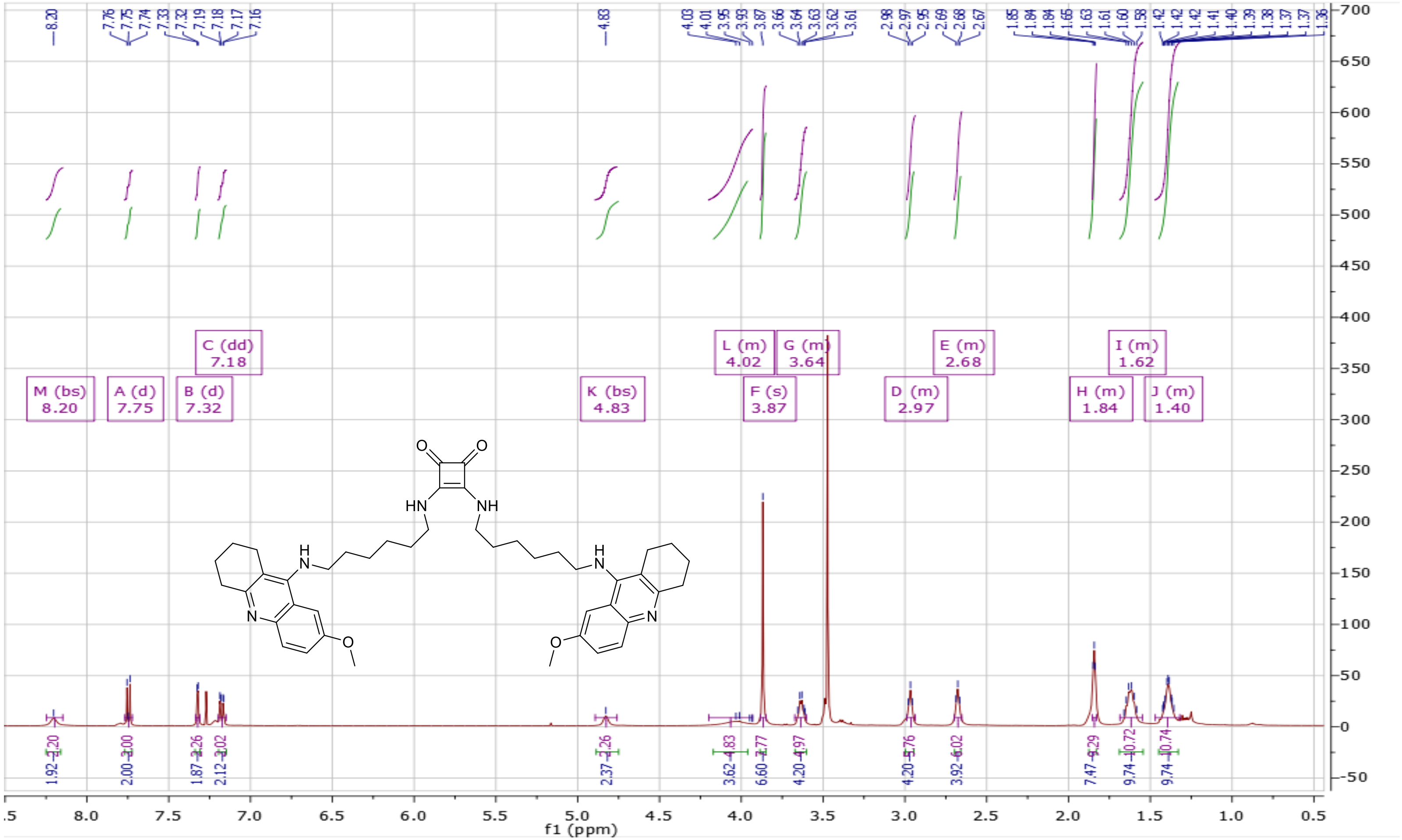

Bis({6-[(7-methoxy-1,2,3,4-tetrahydroacridin-9-yl)amino]hexyl}amino)cyclobut-3-ene-1,2-dione (5e) ^{13}C NMR:

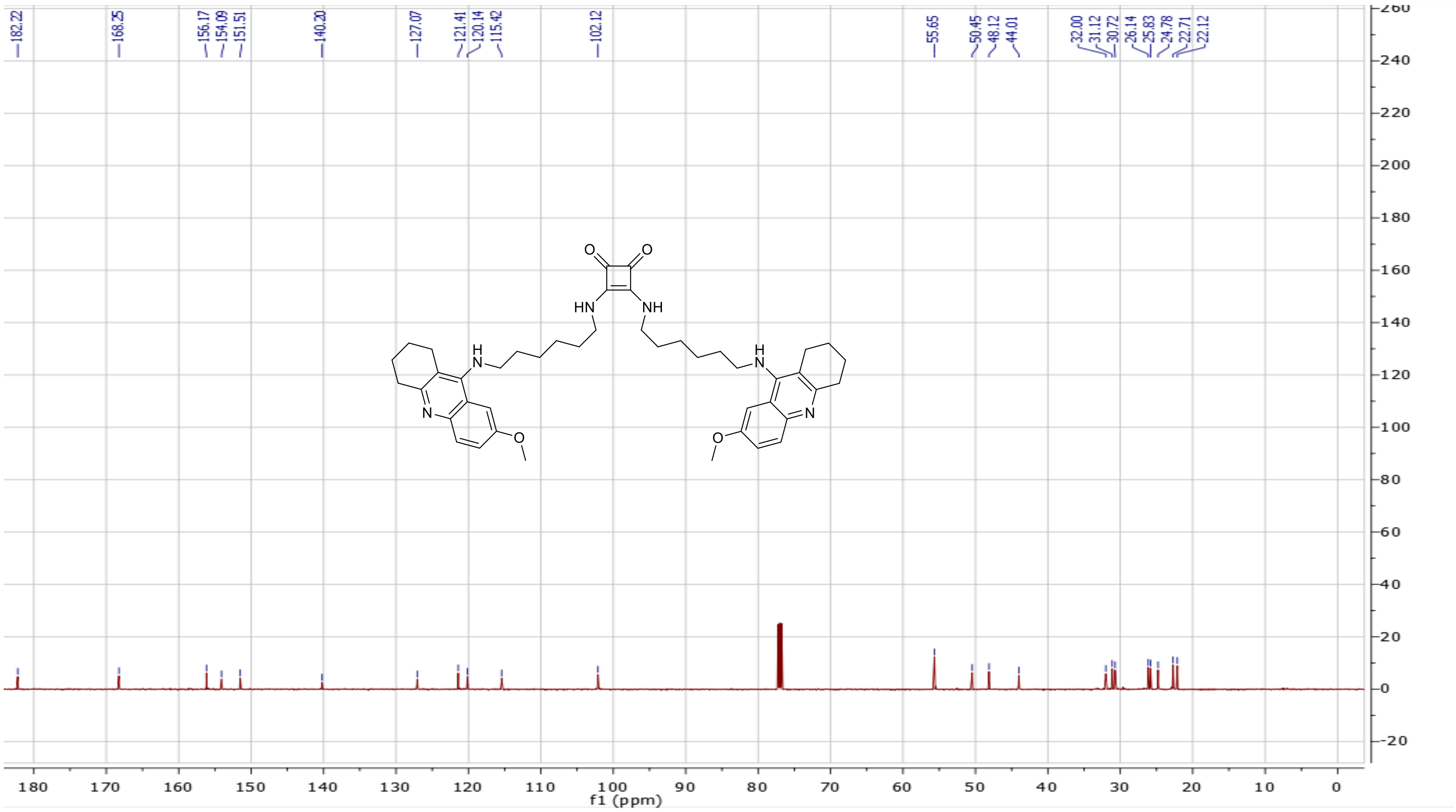

Bis({7-[(7-methoxy-1,2,3,4-tetrahydroacridin-9-yl)amino]heptyl}amino)cyclobut-3-ene-1,2-dione (5f) ^{1}H NMR:

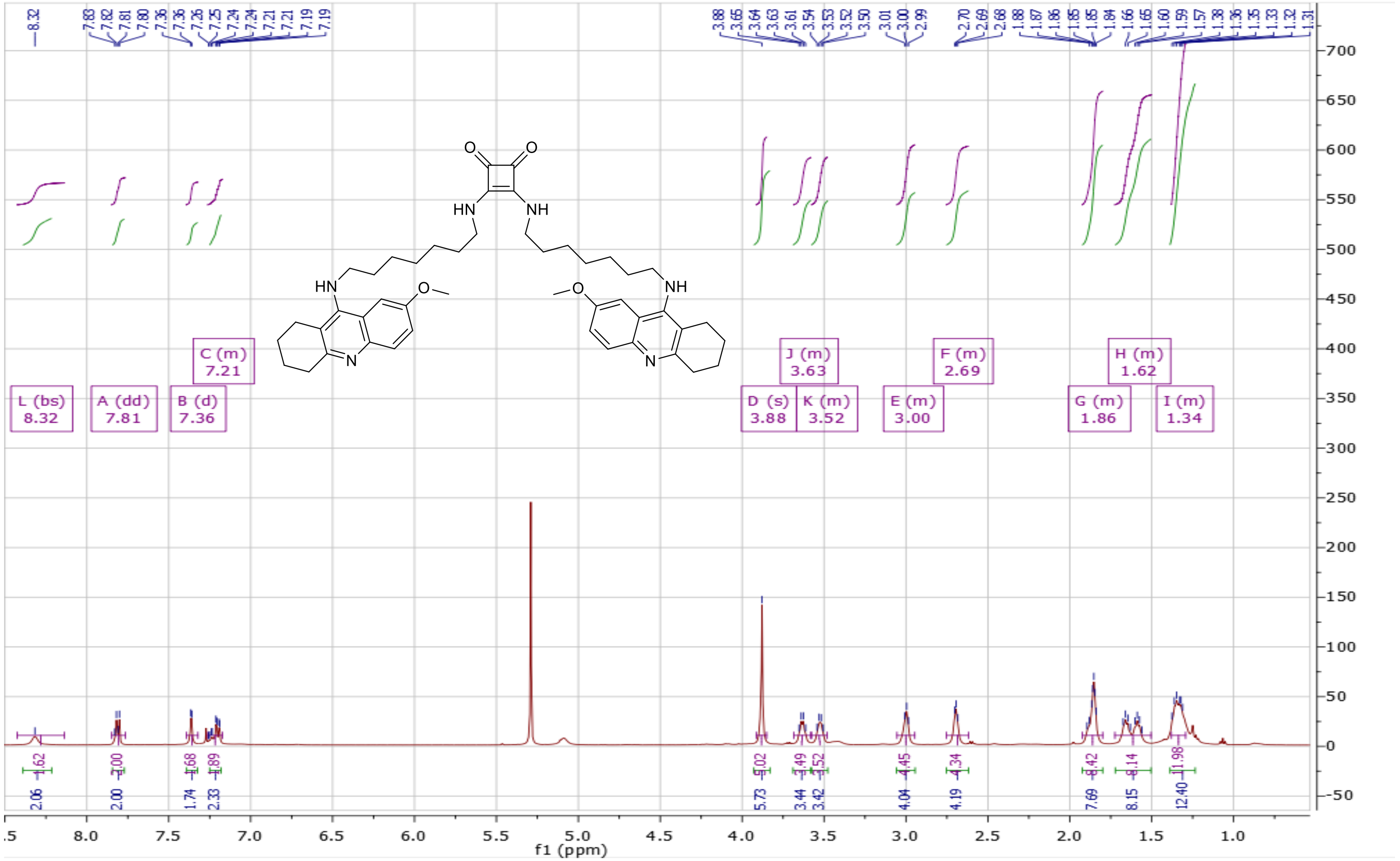

Bis({7-[(7-methoxy-1,2,3,4-tetrahydroacridin-9-yl)amino]heptyl}amino)cyclobut-3-ene-1,2-dione (5f) ^{13}C NMR:

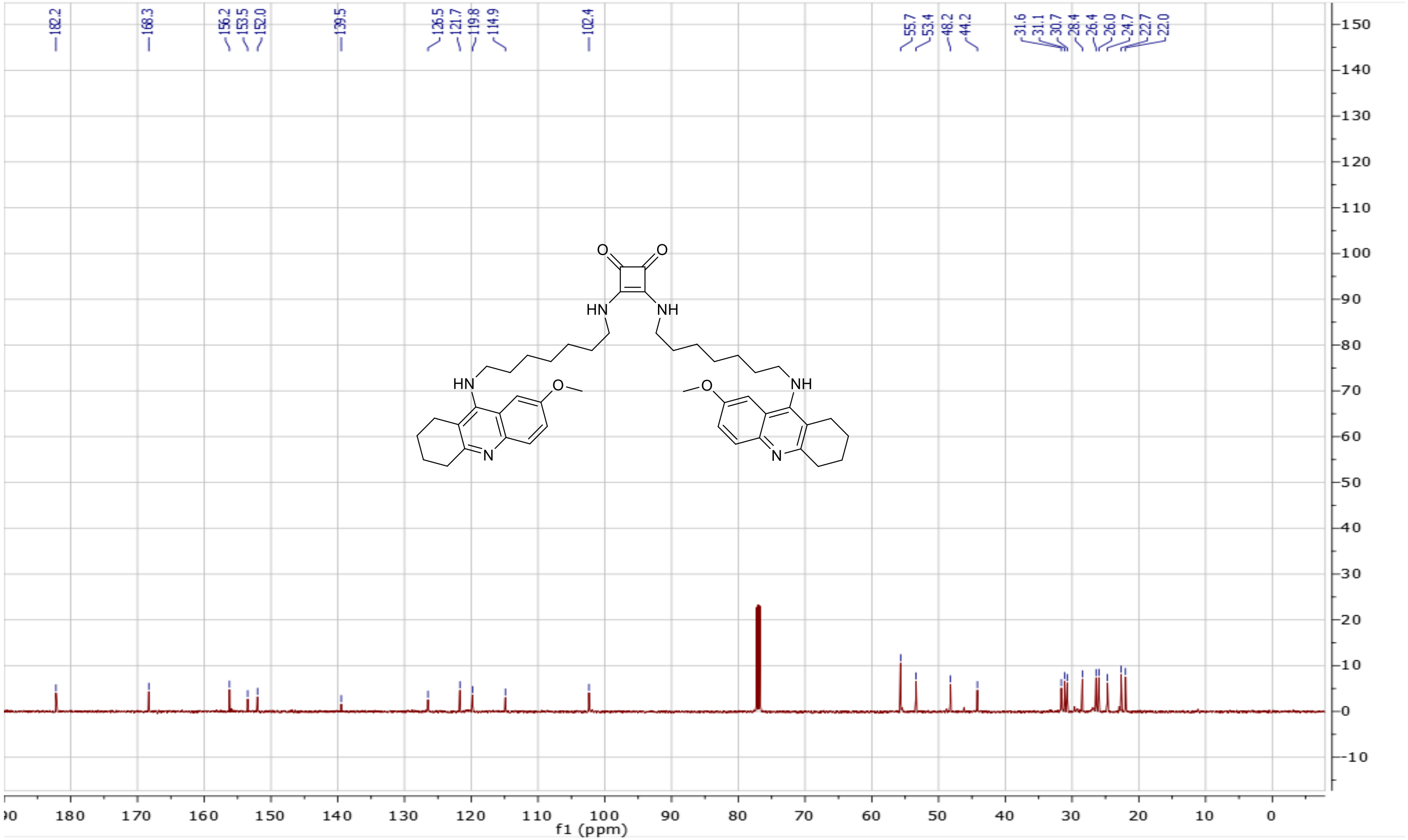

Bis({8-[(7-methoxy-1,2,3,4-tetrahydroacridin-9-yl)amino]octyl}amino)cyclobut-3-ene-1,2-dione (5g) ^{1}H NMR:

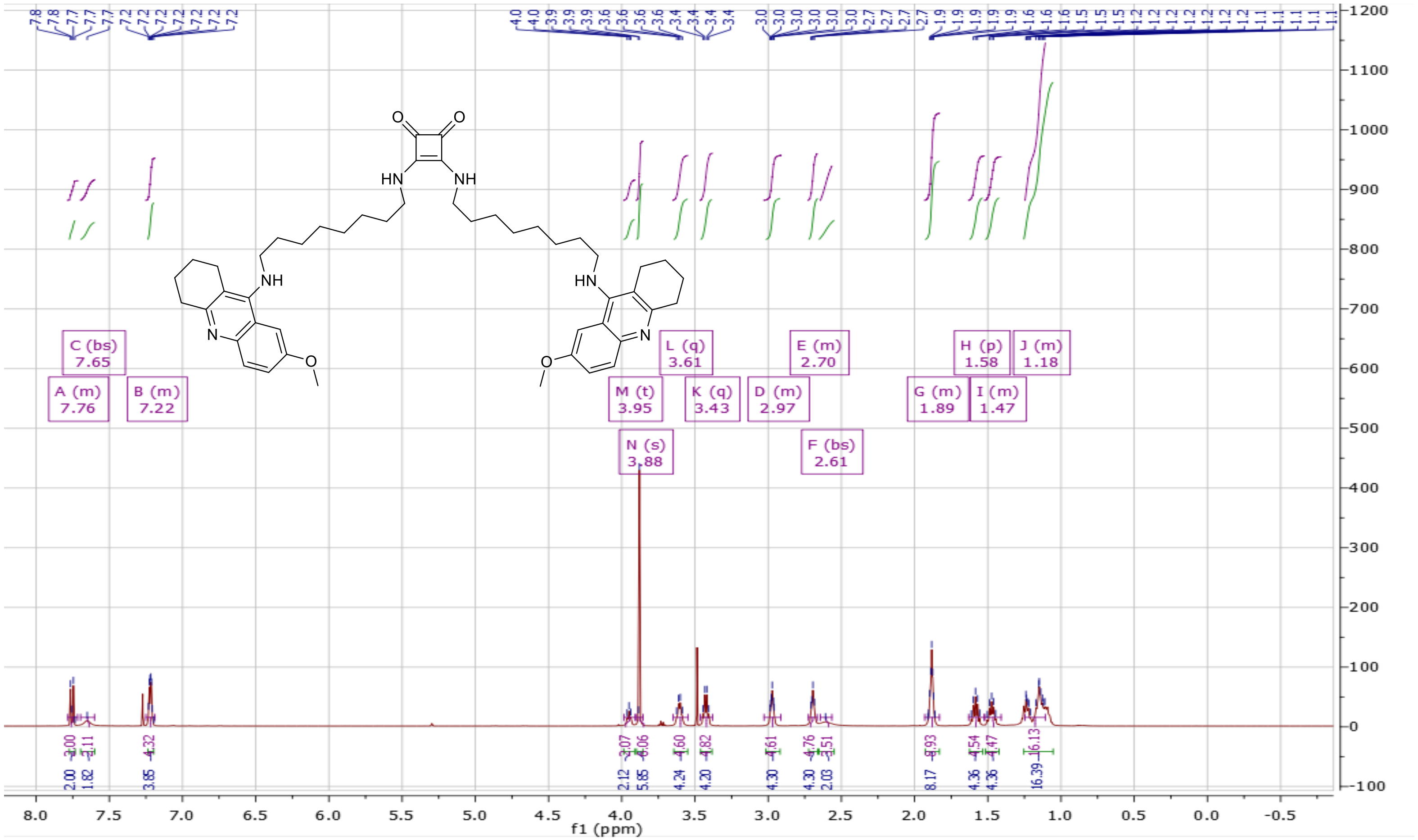

Bis({8-[(7-methoxy-1,2,3,4-tetrahydroacridin-9-yl)amino]octyl}amino)cyclobut-3-ene-1,2-dione (5g) ^{13}C NMR:

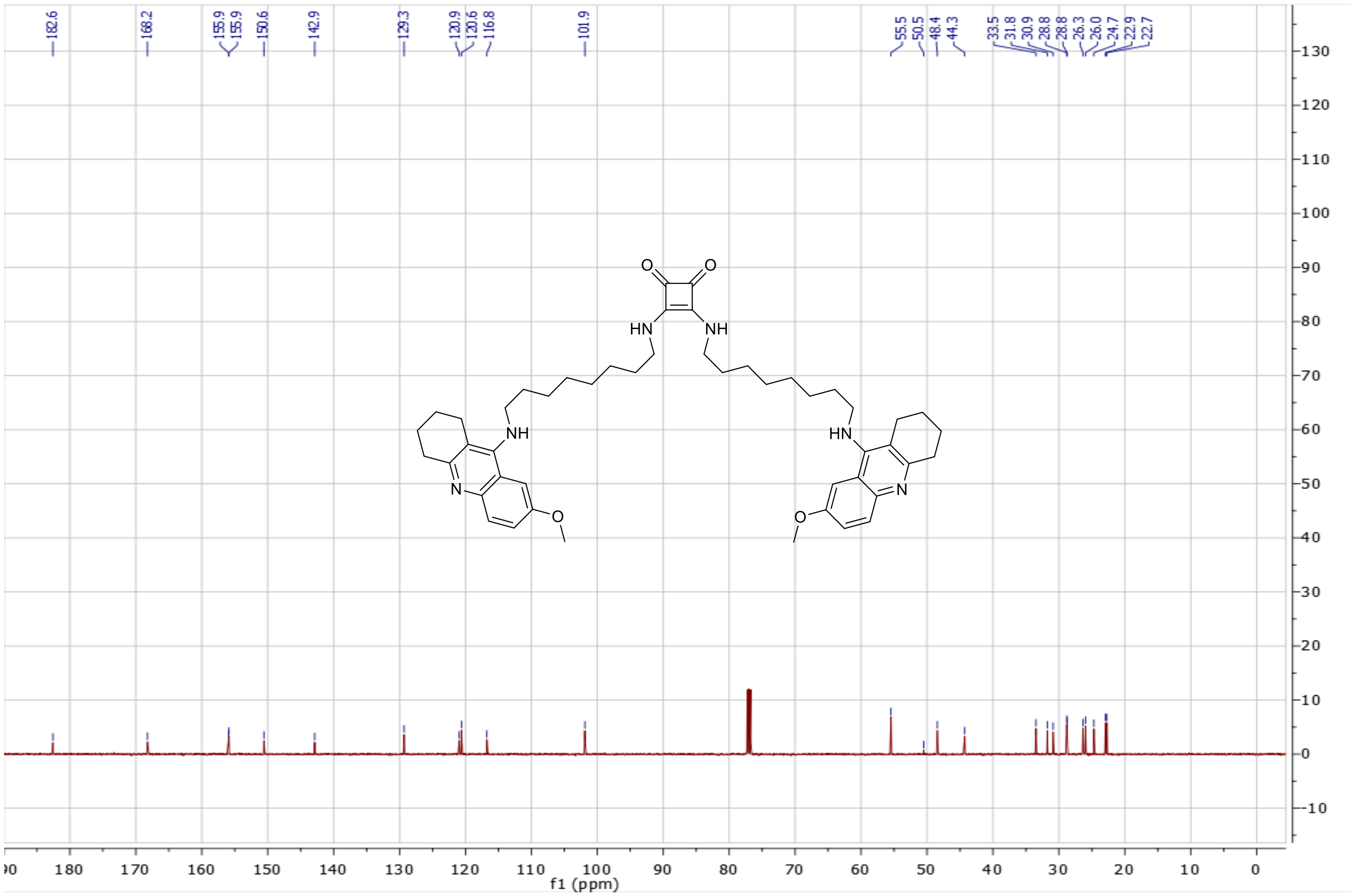

Supplement: Supplementary file 1 [file biomolecules-09-00379-s001.pdf]
